# Expanding the Utility of Bioinformatic Data for the Full Stereostructural Assignments of Marinolides A and B, 24- and 26-Membered Macrolactones Produced by a Chemically Exceptional Marine-Derived Bacterium

**DOI:** 10.3390/md21060367

**Published:** 2023-06-20

**Authors:** Min Cheol Kim, Jaclyn M. Winter, Reiko Cullum, Alexander J. Smith, William Fenical

**Affiliations:** 1 Center for Marine Biotechnology and Biomedicine, Scripps Institution of Oceanography, Skaggs School of Pharmacy and Pharmaceutical Sciences, Moores Comprehensive Cancer Center, University of California, La Jolla, San Diego, CA 92093, USA; 2 Department of Pharmacology and Toxicology, University of Utah, Salt Lake City, UT 84112, USA

**Keywords:** macrolactone, natural product structure elucidation, bioinformatics, genome mining, modular type I polyketide synthase

## Abstract

Marinolides A and B, two new 24- and 26-membered bacterial macrolactones, were isolated from the marine-derived actinobacterium AJS-327 and their stereostructures initially assigned by bioinformatic data analysis. Macrolactones typically possess complex stereochemistry, the assignments of which have been one of the most difficult undertakings in natural products chemistry, and in most cases, the use of X-ray diffraction methods and total synthesis have been the major methods of assigning their absolute configurations. More recently, however, it has become apparent that the integration of bioinformatic data is growing in utility to assign absolute configurations. Genome mining and bioinformatic analysis identified the 97 kb *mld* biosynthetic cluster harboring seven type I polyketide synthases. A detailed bioinformatic investigation of the ketoreductase and enoylreductase domains within the multimodular polyketide synthases, coupled with NMR and X-ray diffraction data, allowed for the absolute configurations of marinolides A and B to be determined. While using bioinformatics to assign the relative and absolute configurations of natural products has high potential, this method must be coupled with full NMR-based analysis to both confirm bioinformatic assignments as well as any additional modifications that occur during biosynthesis.

## 1. Introduction

**Figure 1:**
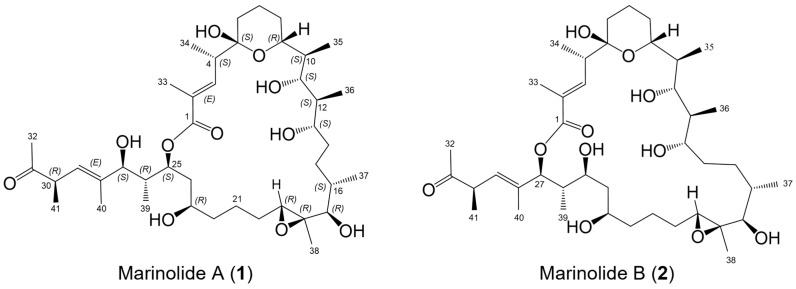
Structures of marinolides A (**1**) and B (**2**) and absolute configurations of stereogenic centers assigned by bioinformatic prediction and subsequently confirmed by NMR-based methods and an X-ray crystallographic structure of **1a**, a ketal derivative of **1**.

The macrolactones, exemplified by the clinically relevant antibiotic erythromycin [1,2,3], are well-known bacterial polyketide metabolites discovered first in the 1950s. Although primarily isolated from bacteria and fungi, numerous macrolactones have been more recently isolated from a variety of marine organisms, including sponges and cyanobacteria [4,5,6]. In bacteria, these metabolites are typically assembled by modular type I polyketide synthases and often possess complex stereostructures. Originally defined as medium-size rings from 14 to 16 carbons in size, more recent macrolactones are recognized to contain very large rings, including the 51-membered stambomycins [7], the 52-membered gargantulides [8,9], and the 60-membered quinolidomicin [10]. Unless they are crystalline and definable by X-ray methods, the confident stereostructural assignments of these macrolides can represent a monumental task. Consequentially, many remain unassigned. Recently, we and others have begun to probe the utility of incorporating bioinformatic analysis, focusing on specific enzymatic domains within modular polyketide synthases that install stereocenters, in assigning the full absolute configurations of polyketide metabolites [9,11,12,13,14,15,16,17]. In this paper, we extend this work to provide the full stereostructures of two new 24- and 26-membered macrolactones, marinolides A (**1**) and B (**2**) (Figure 1).

## 2. Results and Discussion

### 2.1. Marinolides A and B

In recent publications, we demonstrated the biosynthetic potential of a previously unknown marine-derived actinobacterium, our strain AJS-327, that is likely assignable to a new genus within the family Streptomycetaceae [11,12,18]. Annotation of the full genome of this strain using antiSMASH [19] and our own biosynthetic hooks using BLAST^+^ [20] revealed a significant number of type I polyketide biosynthetic clusters, including the recently published gene cluster for the linear polyether-containing polyketide ionostatin [11]. Continued chemical isolation studies, coupled with extensive interpretation of polyketide type I biosynthetic gene clusters in the genome of strain AJS-327, has now facilitated the isolation and full structure elucidation of two new macrolactones, marinolides A and B (**1**, **2**). The marinolides are related by their site of lactonization to produce 24- and 26-membered macrolactones, each possessing a tetrahydropyran hemiketal, five secondary hydroxy groups, an epoxide, and a ketone functionality (Figure 1). Given the presence of 16 chiral centers, which could generate more than 65,000 possible isomers, we again turned to assessing the stereospecificities installed by specific domains encoded in the marinolide polyketide synthases to predict and later confirm the full stereostructures of these macrolactones. 

### 2.2. Marinolide Structure Prediction through Biosynthetic Gene Cluster Analysis

Early HR-LC-MS analysis of the organic extract of the cultured strain AJS-327 illustrated the presence of two molecules, each with the molecular formula C_41_H_70_O_11_, which suggested that the compounds were type I polyketides. With type I polyketide synthase (PKS) systems, the multifunctional enzymes are organized into modules, where each module is typically responsible for one step of chain elongation, and each module encodes a suite of catalytic domains that carry out specific enzymatic reactions [21]. The minimal set of catalytic domains within a module consist of a β-ketoacyl synthase (KS) that catalyzes decarboxylative Claisen-like condensation reactions for C–C bond formation between activated acyl extender units, an acyl carrier protein (ACP) that is post-translationally modified with a prosthetic phosphopantetheine group and used to tether the growing acyl chain, and an acyltransferase (AT) that selects and transfers starter and extender units onto the ACP. In addition to the minimal set of catalytic domains, reductive domains, such as a ketoreductase (KR), dehydratase (DH), and enoylreductase (ER), may be present in a module and can perform varying degrees of reduction in the β-ketothioester-ACP-tethered intermediate. 

**Figure 2:**
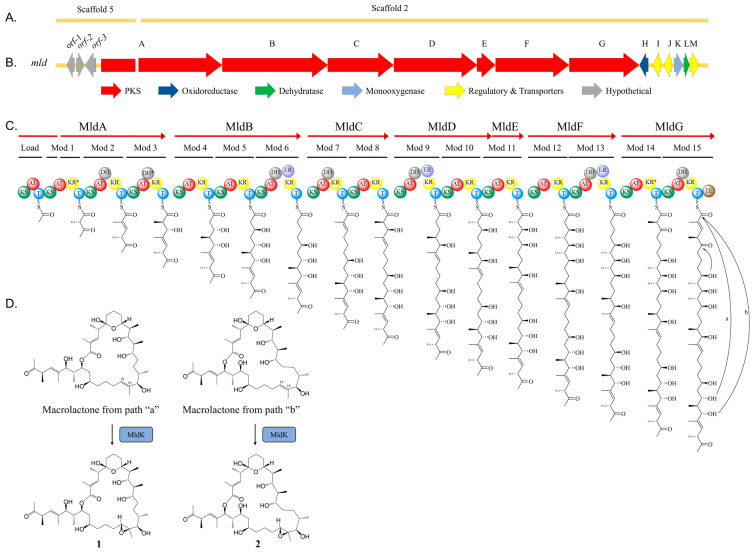
Proposed biosynthesis of marinolides A (**1**) and B (**2**): (**A**) The marinolide biosynthetic gene cluster (*mld*) is located on scaffolds 2 and 5 in the genome of strain AJS-327. (**B**) Organization of the *mld* biosynthetic gene cluster (accession number OL257848). The arrows represent open reading frames, point in the direction of transcription, and are color-coded based on a proposed biosynthetic function. All domains of the loading module in MldA as well as the KS and partial AT domain of module 1 are encoded on scaffold 5, whereas a partial AT domain and the remaining *mld* cluster are found on scaffold 2. (**C**) Biosynthesis of the linear polyketide backbone. The PKS domain abbreviations identified in MldA-MldG are ketosynthase (KS), acyltransferase (AT), dehydratase (DH), enoylreductase (ER), ketoreductase (KR), acyl carrier protein (T), and thioesterase (TE). An asterisk indicates an inactive domain. (**D**) Installation of the epoxide between C-18 and C-19 is predicted to be catalyzed by the flavin monooxygenase MldK after release of the polyketide by the TE domain. The absolute configurations of 1 and 2 were validated using spectroscopic methods and an X-ray structure of the ketal derivative of 1.

**Figure 3:**
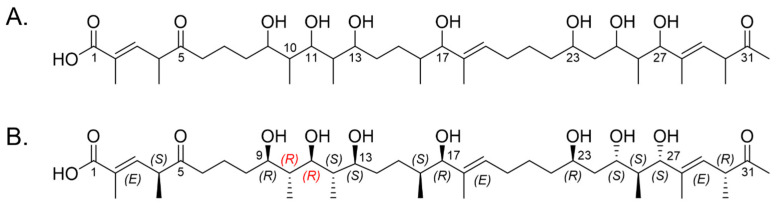
Bioinformatic-predicted structures: (**A**) Predicted linear backbone of the marinolides using domain organization encoded in the *mld* PKS genes, and (**B**) predicted absolute configurations based upon detailed analysis of the KR and ER domain specificities. According to the Cahn–Ingold–Prelog convention, the configurations of C-10 and C-11 (highlighted in red) are ***R*** in the linear representation but converted to ***S*** after formation of the hemiketal ring in **1** and **2**.

To identify the biosynthetic machinery responsible for assembling **1** and **2**, the 6.5 Mbp genome of actinobacterium AJS-327 was scanned for type I polyketide biosynthetic clusters. Out of the 12 PKS biosynthetic gene clusters identified, only two contained the sufficient number of modules required to assemble the hexadecaketide backbones found in **1** and **2**; the previously reported *ion* biosynthetic cluster responsible for ionostatin production [11] and a split PKS biosynthetic cluster spanning across scaffolds 2 and 5 of the assembled genome (Figure 2A). This ~97 kb split cluster, hereby named the *mld* cluster (Genbank accession number OL257848), harbors seven type I PKS genes (*mld*A-G) coding for 16 modules, a flavin monooxygenase (*mld*K), a FAD-dependent oxidoreductase (*mld*H), a short-chain dehydrogenase (*mld*L), and an MFS transporter (*mld*M) (Figure 2B and Figure S33 and Table S4). A more detailed analysis of the *mld* cluster showed that while most of the genes are located on scaffold 2, the first four domains of the PKS *mld*A and a partial AT sequence from module 1 are encoded on scaffold 5 (Figure 2C). An incomplete AT sequence was also annotated in module 11, suggesting that MldD and MldE could, in fact, be one large PKS encoding modules 9−11. Unfortunately, efforts to close the sequencing gaps in modules 1 and 11 using PacBio sequencing, Illumina sequencing, and PCR failed. However, even with the two incomplete AT sequences in modules 1 and 11, key residues and short sequence motifs involved in the starter unit and extender unit selection were present, allowing for the prediction of malonyl-Coenzyme A (CoA) or methyl-malonyl-CoA for all annotated AT domains in the *mld* cluster [22,23,24,25,26]. Bioinformatic analysis of all 16 AT domains revealed that, in addition to the loading module, modules 4, 5, 6, 9, 12, and 13 contain the HAFH motif suggesting a selectivity for malonyl-CoA, whereas modules 1, 2, 3, 7, 8, 10, 11, 14, and 15 contain the YASH motif indicating a preference for methyl-malonyl-CoA (Figure S34). From the annotated AT domains, all 41 carbons in **1** and **2** were therefore accounted for via the incorporation of acetate or propionate from malonyl-CoA or methyl-malonyl-CoA, respectively. This allowed for the full linear and planar structure of the marinolide precursor to be assigned (Figure 3A).

With the planar structure in hand, a detailed bioinformatic investigation of the KR, DH, and ER domains across the seven *mld* PKS genes assisted with assigning the absolute configurations for 13 of the 16 stereogenic centers in **1** and **2** (Figure 3B and Figures S35–S37). In polyketide biosynthesis, KR domains carry out the diastereospecific reduction in ACP-tethered 3-ketoacyl intermediates and are responsible for installing the majority of the stereogenic centers found in polyketide natural products [21,27,28,29,30,31,32]. Based on key residues in the active site, KR domains can be classified into six subtypes: A1, A2, B1, B2, C1, and C2 [27,29,31]. While the C1 and C2 subtypes are redox-inactive, the A1 and A2 subtypes or B1 and B2 subtypes catalyze the reduction of 3-ketoacyl-ACP to either (3*S*)-3-hydroxyacyl-ACP or (3*R*)-3-hydroxyacyl-ACP, respectively. If methyl-malonyl-CoA is loaded onto the module, the A2 and B2 subtypes can also epimerize the methyl substituent and convert (2*R*)-2-methyl-3-ketoacyl-ACP to (2*S*,3*S*)-2-methyl-3-hydroxyacyl-ACP or (2*S*,3*R*)-2-methyl-3-hydroxyacyl-ACP, respectively. Interestingly, even though they are redox-inactive, the C2 subtypes have also been shown to convert (*2R*)*-*2-methyl-3-ketoacyl-ACP to (2*S*)-2-methyl-3-ketoacyl-ACP [33]. By aligning the KR domains from the *mld* PKSs to characterized bacterial KR sequences, we were able to assign their respective subtypes and predict the absolute configurations of the C-9, C-11, C-13, C-17, C-23, C-25, and C-27 hydroxyl groups in the linear backbone of **1** and **2**, as well as the methyl substituents at C-4, C-10, C-12, C-16, C-26, and C-30 (Figure 2C, Figure 3B and Figure S35). KR domains from modules 1 and 14 were predicted to be redox-inactive and belonged to the C2 and C1 subtypes, respectively. Sequences belonging to the A1 subtype included KR domains from modules 5, 8, and 12, KR domains belonging to the A2 subtype were encoded in modules 10 and 11, and KR domains belonging to the B1 subtype were identified in modules 2, 3, 4, 6, 7, 9, 13, and 15 (Figure S35).

After assigning the modifications installed by the 15 ketoreductases, we turned our attention to the DH domains in the *mld* PKS genes. In polyketide biosynthesis, DH domains, if present, catalyze the formation of a 2,3-enoyl-ACP intermediate by abstracting the α-proton and eliminating water. Aligning conserved residues from the seven *mld* dehydratases revealed that while modules 2, 6, 7, 9, 13, and 15 appeared to be active [34], the DH domain in module 3 was missing the catalytic aspartic acid residue and therefore predicted to be inactive (Figure 2C and Figure S36). Thus, this results in the (2*R*, 3*S*)-2-methyl-3-hydroxylacyl tetraketide instead of the 2-methyl-2,3-enoylacyl intermediate. Finally, following analysis of the DH domains, we interrogated the ER domains within modules 6, 9, and 13 to predict whether a fully reduced polyketide intermediate would be formed. As observed with ketoreducases, ER domains can also control the ***R*** or ***S*** configuration of the 2-methyl substituent as they catalyze the stereospecific reduction in 2,3-enoyl-ACP-tethered intermediates when methyl-malonyl-CoA is selected as a building block [35,36]. Analysis of the ER domains identified in modules 6, 9, and 13 appeared to be functional and contained the conserved tyrosine residue at position 44, which would catalyze the formation of a (*2S)*-2-methyl-acyl product (Figure S37). However, the AT prediction for each of the corresponding modules indicates a preference for malonyl-CoA. Thus, there would be no ER-catalyzed epimerization activity on the ACP tethered intermediates. Remarkably, using the KR and ER domain specificities from the *mld* PKSs, 13 of the final 16 stereogenic centers in **1** and **2** were predicted reliably and were in agreement with the subsequent X-ray data derived from derivative **1a** and final NMR-based structures. As the installation of the epoxide moiety and formation of the tetrahydropyran hemiketal were not deduced from the biosynthetic machinery, the stereocenters at C-5, C-18, C-19 were not initially assigned by bioinformatic analysis. 

Based on the bioinformatic investigation of the Mld PKS genes and tailoring enzymes identified in the *mld* biosynthetic cluster, we can propose a biosynthetic pathway for **1** and **2** (Figure 2). The hexadecaketide backbone is biosynthesized by MldA–G in an assembly-line-like manner, and the release of the polyketide product from MldG is facilitated by the thioesterase (TE) domain via macrolactonization with either the C-25 or C-27 hydroxy group to afford the 24- or 26-membered macrolactone, respectively (Figure 2C). After release from MldG, it is speculated that MldK, a putative flavin monooxygenase [37], oxidizes the olefin between C-18 and C-19 into the epoxide (Figure 2D). However, before the hexadecadetide is released from MldG, it is proposed that formation of the tetrahydropyran ring occurs while the polyketide is tethered to an ACP, presumably on module 14 or 15. In salinomycin biosynthesis, the installation of pyran ring A also occurs on a PKS-bound intermediate and SalBIII, a pyran synthase, was shown to catalyze this intramolecular cyclization reaction [38]. Unfortunately, we were unable to identify any *sal*BIII homologs in the *mld* cluster, and alignment of SalBIII with MldH, MldL, and MldK revealed extremely low sequence similarities of 12%, 14%, and 13%, respectively. As several types of enzymatic reactions that form oxygen heterocycles have been characterized, e.g., oxa-Michael additions and intramolecular epoxide ring openings [39,40,41], the tetrahydropyran ring in marinolide A and B is most likely formed through a nucleophilic addition reaction between the C-5 ketone and C-9 alcohol. Based on the additional genes present in the *mld* cluster, the putative FAD oxidoreductase *mld*H, the short-chain dehydrogenase *mld*L, and/or the flavin monooxygenase *mld*K could be responsible for this reaction. As observed with ambruticin biosynthesis [42], we cannot rule out the possibility that a dual functioning dehydratase could also catalyze formation of the pyran ring. 

### 2.3. Isolation of Marinolides A and B (1, 2)

With the LC-MS data for **1** and **2** in hand as a guide, we processed a large-scale cultivation (20 L) of AJS-327 using a seawater-based A1 medium. Extraction using solid-phase resin (Sephadex XAD-7) methods, followed by acetone extraction of the resin gave the organic extract (3.5 g) that was first fractionated using standard silica gel methods. Reversed phase LC-MS analysis showed that fraction 7 contained the two metabolites assigned as marinolides A and B (**1**, **2**). Semi-preparative C-18 reversed-phase purification generated purified samples of each macrolactone.

### 2.4. Confirmation of the Structures of Marinolides A and B by Spectroscopic Methods

Marinolide A (**1**) was isolated as a non-crystalline gum, and its molecular formula was assigned as C_41_H_70_O_11_ based on a pseudomolecular sodium adduct ion at *m*/*z* 761.4823 [M + Na]^+^, and by analysis of HR-ESI-TOFMS data coupled with ^13^C NMR data. A broad IR absorption band at 1704 cm^−1^ suggested the presence of an ester group and a ketone, an assignment supported by the presence of two carbonyl signals at *δ*_C_ 210.5 and 168.7 in the ^13^C NMR spectrum. The 1D and 2D NMR data (Figures S1–S15) confirmed the presence of 41 carbons, including the ketone and ester carbonyl carbons, four quaternary carbons, two olefinic methine carbons, eight oxymethine carbons, six aliphatic methine carbons, nine methylene carbons, and ten methyl carbons. These carbon signals accounted for four of the seven degrees of unsaturation deduced from the molecular formula, indicating that marinolide A (**1**) was composed of three rings (Table S1). 

**Figure 4:**
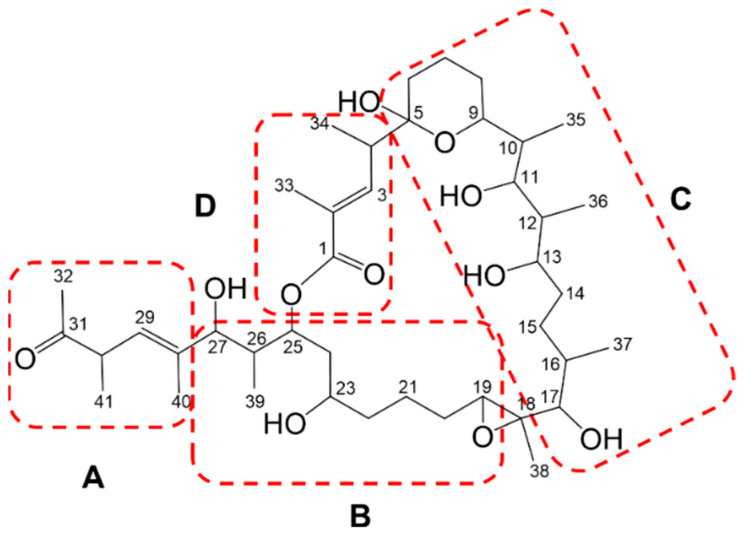
Substructure assignments and carbon numbering scheme for marinolide A (**1**). Substructure A consists of carbons C-28 to C-32, substructure B consists of carbons C-19 to C-27, substructure C consists of carbons C-5 to C-17, and substructure D consists of carbons C-1 to C-4.

The ^1^H and HSQC NMR spectra of **1** displayed obvious signals for the two olefinic protons at *δ*_H_ 7.16 and 5.48, eight oxymethine protons between *δ*_H_ 5.80 and 2.85, six methine protons between *δ*_H_ 3.18 and 1.47, nine methylene groups between *δ*_H_ 2.15 and 1.01, six secondary methyl groups at *δ*_H_ 1.22, 1.05, 0.96, 0.88, 0.83, and 0.55, two tertiary methyl groups at *δ*_H_ 1.86 and 1.37, and two olefinic methyl groups at *δ*_H_ 1.99 and 1.67. Detailed analysis of the ^13^C and HSQC NMR spectra of **1** allowed all protons to be assigned to their respective carbons. The 1D and 2D NMR spectra data, including correlations from COSY and TOCSY experiments, showed four continuous spin systems assigned as substructures A–D (Figure 4). However, many methylene and methine signals were overlapping between *δ*_H_ 1.0 and 2.0 and difficult to assign. An HSQC-TOCSY NMR experiment was then utilized to clarify the overlapping and clustering spin systems that were difficult to distinguish in each substructure (Figure S10 and Table S2). Consequently, the four spin systems were well established by COSY, TOCSY, and HSQC-TOCSY NMR techniques. Comprehensive analysis of 1D and 2D NMR spectra led to the construction of substructure A (C-28 to C-32) (Figure 4). The methyl ketone group (C-31–C-32) and methyl group (C-40) were placed at C-30 (*δ*_C_ 46.3) and C-28 (*δ*_C_ 139.8) by analysis of HMBC NMR data. Substructure B (C-19 to C-27) was assigned by analysis of COSY, TOCSY, HSQC-TOCSY, and HMBC data. Based on their ^13^C chemical shifts, *δ*_C_ 63.9, 68.2, 76.1, and 77.9, C-19, C-23, C-25, and C-27 were assigned as oxygenated carbons. Interpretation of 2D NMR data also led to the assignment of substructure C (C-5 to C-17), which possesses a tetrahydropyran hemiketal. COSY and HMBC NMR data showed the three methyl groups, C-35 (*δ*_H_ 0.88, d, *J* = 7.0), C-36 (*δ*_H_ 0.55, d, *J* = 6.7), and C-37 (*δ*_H_ 1.22, d, *J* = 6.3) were positioned at C-10 (*δ*_C_ 38.1), C-12 (*δ*_C_ 40.5), and C-16 (*δ*_C_ 36.5), respectively. Finally, substructure D (C-1 to C-4) was assigned by analogous interpretation of 2D NMR data. HMBC NMR correlations of the H-3 olefinic proton (*δ*_H_ 7.19) to the C-1 carbonyl carbon (*δ*_C_ 168.7), to C-33 (*δ*_C_ 13.3), and to C-34 (*δ*_C_ 14.4), as well as correlations of H_3_-33 (*δ*_H_ 1.99) to C-1, C-2 (*δ*_C_ 130.6), and C-3 (*δ*_C_ 141.9) demonstrated that substructure D contained the lactone functional group. 

Subsequently, HMBC correlations from the olefinic methyl group (H_3_-40) (*δ*_H_ 1.67) to C-27 (*δ*_C_ 77.9), C-28 (*δ*_C_139.8), and C-29 (*δ*_C_126.6) established the linkage of substructures A and B. Further, HMBC NMR correlations of the oxygenated methine proton H-19 (2.91, dd, *J* = 9.9, 3.1) to C-18 (*δ*_C_ 63.8) and C-20 (*δ*_C_ 29.9), the methyl group H_3_-38 (1.37, s) to C-17 (*δ*_C_ 83.0), C-18 and C-19 (*δ*_C_ 63.9), and of the oxygenated proton H-17 (2.85, d, *J* = 10.3) to C-16 (*δ*_C_ 36.5), C-18, C-37 (*δ*_C_ 16.7), and C-38 (*δ*_C_ 10.8) indicated connectivity between substructures B and C and strongly suggested the presence of an epoxide ring at C-18-19. In addition, an HMBC correlation was observed between H-4 in substructure D and a quaternary carbon C-5 (*δ*_C_ 99.1), which is a characteristic value for a hemiketal carbon, indicated connectivity between substructures C and D. Substructures B and D were linked through HMBC correlations between H-25 and C-1 in the HMBC spectrum obtained in CD_3_OD (Figure S15). Consequently, the planar structure of marinolide A (**1**) was confirmed as a 24-membered macrolactone featuring a tetrahydropyran hemiketal and epoxide rings. Comparison of the planar NMR-based structure of **1** was a perfect match to the predicted structure as assigned by gene cluster analysis.

### 2.5. Relative Configurations of Marinolides by Spectroscopic Methods

**Figure 5:**
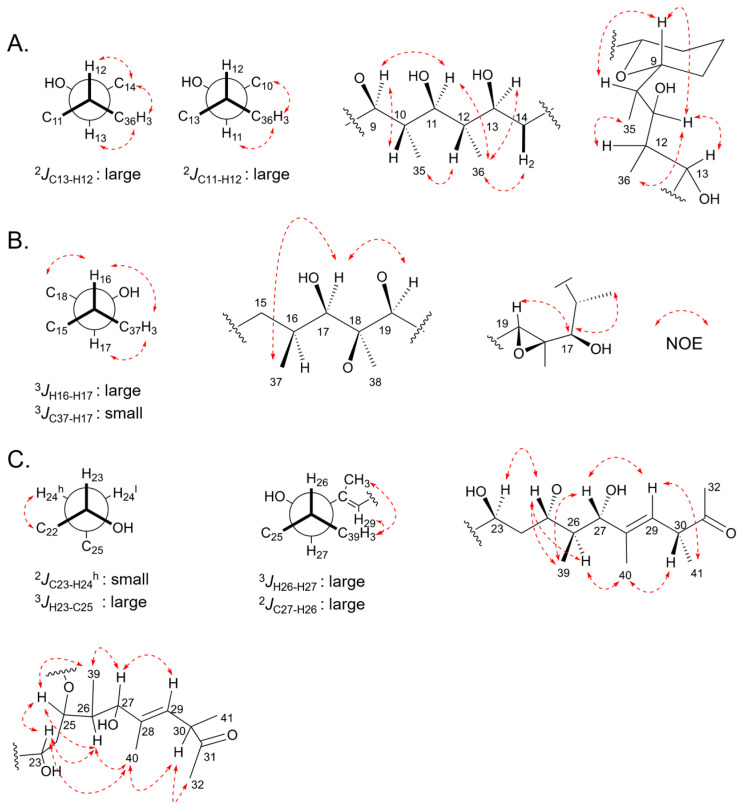
NMR relative configuration assignments for components of marinolide A (**1**) on the basis of NOE results, *J*_H-H_ and *J*_C-H_ coupling constant analyses: (**A**) configuration from C-9 to C-14; (**B**) C-16 to C-19; (**C**) C-23 to C-32.

Analysis of NMR data, in particular the number of methine carbons present, indicated marinolide A possessed 16 asymmetric centers. Although the configurations of most of these centers were predicted via bioinformatic analysis of the reductive domains in the polyketide synthases MldA–G, confirmation of the relative configurations of **1** were approached using *J*-based configuration analysis using HETLOC and ROESY NMR analysis [43]. The two double bonds were confirmed to be in *E* configurations by the observation of NOE correlations between H-4 and H_3_-33 and between H-30 and H_3_-40 (Table S3). The relative configuration from C-11 to C-12 was confirmed by assignment of the vicinal methine carbons. A large heteronuclear coupling constant between C-13/H-12 (8.9 Hz) indicated that H-12 was *gauche* to the electronegative oxygen substituent at C-11. Additionally, the NOE correlations of H_3_-36/H-13, H_3_-36/H-14, and H-12/H-14 were observed; otherwise H-11/H-12 was not detected. These data are consistent with an *erythro* rotamer system. Additionally, C-11 to H-12 (9.2 Hz) has a large coupling constant with NOE correlations of H_3_-36 to H-10 and H_3_-36 to H-11, indicative of the *erythro* rotamer. Based on these data, we assigned the C-10,11,12,13 configuration as *anti*/*anti*/*anti.* In addition, NOE correlations of H-9 to H-10 and H-9 to H-11 further indicated the C-9,10,11 configuration was *anti*/*anti* (Figure 5A). The relative configurations of C-16 to C-19, composed of a methyl group bearing an epoxide ring, a secondary methyl group, and a hydroxy group were assigned as C-16/17 *syn*, and the epoxide as *trans*. The C-16/C-17 configuration was based on a large ^3^*J*_H,H_ of H-16 with H-17, and no NOE correlation between H_3_-37 and H_3_-38. Additionally, NOE NMR correlations were not observed between H-19 and H_3_-38. However, NOE interactions between H-16/H_3_-38 and H-17/H-19 were detected (Figure 5B). Finally, the relative configurations at C-23 to C-30 were confirmed as in Figure 5C. The C-23/C-24 configuration was based on a small ^3^*J*_H,H_ of H-23 with H-24 (0.0 Hz), a large ^2^*J*_H,C_ of C-24 with H-23 (9.5 Hz). These *J* values supported only one rotameric system. The configuration at C-26/C-27 was assigned on the basis of a large ^3^*J*_H,H_ of H-26 with H-27 (9.3 Hz) and a large ^2^*J*_H,C_ coupling constant of C-27 with H-26 (6.5 Hz). Additionally, NOE correlations of H-23/H-25, H-23/H_3_-40, H-25/H-26, H-25/H_3_-39, H-26/H_3_-40, H-27/H_3_-39, H-27/H-29, H-29/H_3_-41, H-30/H_3_-32, H-30/H_3_-41 were observed.

### 2.6. X-ray Structure of a Bis-Acetonide Derivative (1a) of Marinolide A

**Figure 6:**
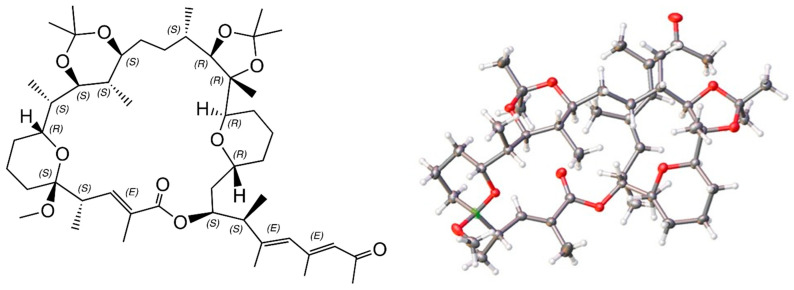
ChemDraw and ORTEP X-ray structure drawing of ketal derivative **1a.** The absolute stereochemistry was conclusively assigned in the X-ray experiment (Flack = 0.06(5)). In the ORTEP structure, oxygen atoms are highlighted in red, hydrogen atoms in white, and carbon atoms in gray. Note that the side chain in the ORTEP drawing is folded under the main ring. The side chain in the ChemDraw structure has been extended outward to better illustrate its configuration.

To potentially create a usable derivative of **1**, we obtained a crystalline *bis*-acetonide product from **1** by treatment with dimethoxy propane and *p*-tosylic acid. Based upon the crystal structure of **1a**, we observed several unexpected results, i.e., dehydration yielding a new double bond, a new tetrahydropyran ring, and one new methoxy group. Most of the expected components of marinolide A (**1**) remained. The ORTEP X-ray drawing, which provided the absolute stereostructure for **1a** is shown in Figure 6. Importantly, the relevant asymmetric centers in **1a** perfectly matched bioinformatic predictions.

Marinolide B (**2**) was also isolated as a white gum, and the HR-ESI-TOFMS data indicated the same molecular formula as **1**. Additionally, the ^1^H and ^13^C NMR spectral data of **2** were very close to those of **1** (Table S1). HMBC NMR correlations from H-27 to C-1 and other analysis of 2D NMR spectral data for **2** revealed that the planar structure of **2** was a 26-membered macrolactone, produced by cyclization between C-1 and C-27 instead of C-1 and C-25 to form the lactone bond in **1** (Figure 2C). The relative configurations of **2** were also determined by *J*-based configuration analysis (HETLOC) and ROESY NMR analysis. On the basis of bioinformatic and X-ray data from **1a**, the absolute configuration of **2** was also assigned as 4*S*, 5*S*, 9*R*, 10*S*, 11*S*, 12*S*, 13*S*, 16*S*, 17*R*, 18*R*, 19*R*, 23*R*, 25*S*, 26*S*, 27*S*, 30*R*.

### 2.7. In Solution Conformation of Marinolide A (1)

**Figure 7:**
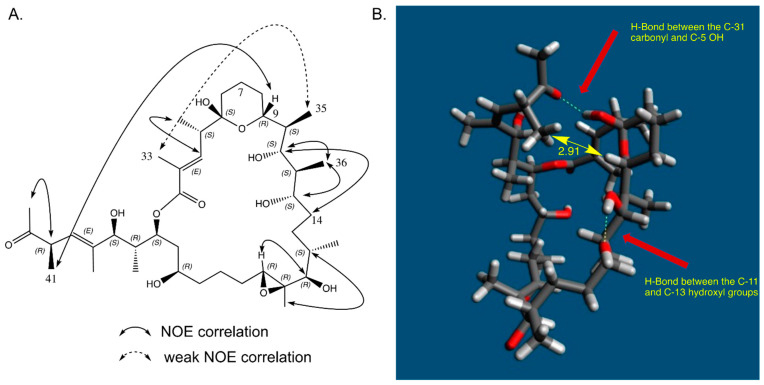
ROESY NMR correlations for marinolide A in C_6_D_6_ solution (see Table S3): (**A**) Particularly noteworthy are NOE correlations between the methyl protons at the C-41 and the C-9 proton, as well as a weaker correlation from methyl C-33 to methyl C-35, indicative of the side chain folded under the ring in the benzene-*d*_6_ solution. (**B**) Modeling of the NOE data using Avagadro2 software shows that the C-41 methyl group and the proton on C-9 are likely 2.91 Angstroms apart and also shows likely hydrogen bonds between the C-5 OH and the C-31 carbonyl as well as the OH groups on C-11 and C-13.

As part of ROESY experiments in benzene-*d*_6_ (Table S3.), we noticed unusually strong correlations between protons that were quite distant from one another. Evaluation of these NOE correlations indicated that the side chain in **1** from C-26 to C-32 was folded beneath the 24-membered ring in non-polar solvents (Figure 7A). Based upon an inspection of molecular models, and applying the modeling software Avogadro2, it appears that this folded conformation is likely stabilized by a hydrogen bond between the C-5 hydroxy proton and the C-31 carbonyl (Figure 7B).

## 3. Materials and Methods

### 3.1. General Experimental Procedures

Optical rotations were recorded using a JASCO P-2000 polarimeter with a 1 cm cell. UV spectra were obtained with a Beckman Coulter DU800 spectrophotometer with a path length of 1 cm. IR spectra were recorded on a Perkin-Elmer 1600 FT-IR spectrometer. The 1D and 2D NMR spectroscopic data were obtained on a JEOL 500 NMR spectrometer. X-ray data for **1a** were obtained using a Bruker Smart APEX II CCD diffractometer equipped with Cu K_α_ radiation (λ = 1.54178 Å). The chemical shift values are reported in ppm units and coupling constants are reported in Hz. NMR chemical shifts were referenced to the residual solvent peaks (*δ*_H_ 7.16 and *δ*_C_ 128.0 for benzend-*d*_6_). High-resolution ESI-TOF mass spectral data were measured on an Agilent 6530 Accurate-Mass Q-TOF LCMS spectrometer coupled to an Agilent 1280 LC system with a Phenomenex Luna C18 column (4.6 × 100 mm, 5 µm, flow rate 0.7 mL/min). Preparative HPLC separations were performed using a Shimadzu SCL-10A chromatograph with a Shimadzu SPD-10A UV/Vis detector and a reversed-phase C18 column (Phenomenex Luna, 10.0 × 250 mm, 10 µm) at a flow rate of 3.0 mL/min.

### 3.2. Isolation and Identification of Strain AJS-327

Strain AJS-327 was isolated from a detached sponge fragment (unidentified) collected in December 2016 on the beach 200 m south of Scripps Institution of Oceanography Pier in La Jolla, CA. The sponge sample was collected in a sterile 50 mL tube and transported within one hour to the laboratory, where it was cut into small fragments with sterile scissors, and fragments were streaked onto seawater A1 medium-based agar plates (10 g of soluble potato starch, 4 g of yeast extract, 2 g of peptone, 750 mL of naturally sourced and filtered seawater, 250 mL of distilled H_2_O, and 18 g of agar). Strain AJS-327 was isolated using the sterile loop method and subcultured using the same agar medium. The strain was identified by partial 16S rDNA sequence analysis (1380 bp) using NCBI BLASTn search (GenBank accession no. MK817028). The closest matching type strains all illustrated 96% identity. The poor matches strongly indicate that our strain represents a novel lineage, likely a new genus within the actinomycete family *Streptomycetaceae*.

### 3.3. Cultivation, and Extraction of Strain AJS-327

Strain AJS-327 was initially cultured in a 1L volume using a seawater-based A1 medium (10 g of starch, 4 g of yeast, 2 g of peptone, 300 mL of deionized water, and 700 mL of seawater), shaking at 180 rpm for 4 days at 27 °C. The 1 L culture medium was used to inoculate 20 × 2.8 L Thomson Ultra-Yield flasks each containing 1 L of seawater-based A1 medium and shaken at 180 rpm at 27 °C. After 7 days of cultivation, 20 g of Amberlite XAD-7 resin were added to each flask, and shaking was continued at 180 rpm for 4 h. The resin was collected by filtration through cheesecloth and washed with deionized water. The washed resin was extracted with acetone, and the solvent was removed under vacuum. The remaining solution was then extracted with ethyl acetate, and the ethyl acetate layer was collected and evaporated under reduced pressure to yield 3.5 g of organic extract.

### 3.4. Isolation of Marinolides A (1) and B (2)

The organic extract (3.5 g) was subjected to silica vacuum flash chromatography, using a step gradient with a solvent gradient of *n*-hexane, EtOAc, and MeOH (1:0:0, 10:1:0, 5:1:0, 2.5:1:0, 1:1:0, 0:1:0, 0:20:1, 0:7:1, 0:2:1, 0:0:1; each 400 mL) to yield 10 fractions. Fraction 7 (760 mg) was re-fractionated by C18 vacuum flash chromatography elution with H_2_O and MeOH (20%, 40%, 60%, 70%, 80%, 90%, and 100%) to afford seven sub-fractions. The 70% MeOH fraction (190 mg) that contained the marinolides A (**1**) and B (**2**) was concentrated under vacuum and further purified via reversed-phase semi-prep HPLC using an elution gradient from 60% to 100% MeOH-H_2_O over 30 min to yield compounds **1** and **2**. Marinolides A and B (**1**, **2**) were further purified via reversed-phase semi-prep HPLC (Phenomenex Luna C-18 column, 250 × 10, 10 µm; 3 mL/min; 73% isocratic MeOH-H_2_O over 40 min; UV detection at 280 nm) to yield **1** (11 mg, *t*_R_ 30.2 min) and **2** (4.5 mg, *t*_R_ 35.7 min).

*Marinolide A (**1**)*: white gum, ${\left[\alpha \right]}_{D}^{20}$ − 9.8 (*c* 0.75, CH_3_OH); IR (ZnSe) *ν*_max_ 3438, 2965, 2361, 1704 (broad), 1570, 1422, 1032 cm^−1^; UV (MeOH) *λ*_max_ (log ε) 214 (4.23), 282 (2.76) nm; ^1^H and ^13^C NMR data, see Tables S1–S3; HR-ESI-TOFMS: [M + Na]^+^ *m*/*z* 761.4823 (calcd. for C_41_H_70_O_11_Na, 761.4816).

*Marinolide B (**2**)*: white gum, ${\left[\alpha \right]}_{D}^{20}$ − 27.8 (*c* 1.0, CH_3_OH); IR (ZnSe) *ν*_max_ 3456, 2972, 2362, 1710, 1451, 1382, 1263, 1032 cm^−1^; UV (MeOH) *λ*_max_ (log ε) 215 (4.12), 282 (2.90) nm; ^1^H and ^13^C NMR data, see Table S1; HR-ESI-TOFMS: [M + Na]^+^ *m*/*z* 761.4852 (calcd. for C_41_H_70_O_11_Na, 761.4816).

### 3.5. Preparation of Bis-Acetonide 1a

Marinolide A (**1**, 5 mg) was dissolved in 2,2-dimethoxypropane (2 mL), methanol (1 mL), and *p*-TsOH (3 mg) was added and stirred at 4 °C for 1 h, then stirred at room temperature for 11 h. The reactants were quenched with saturated aqueous NaHCO_3_ (1 mL), and the reaction mixture was extracted with CH_2_Cl_2_ (3 mL × 3). The residue was purified by C18 reversed-phase HPLC 100% MeOH to provided compound **1a** (2 mg, *t*_R_ 14.5 min). ^1^H NMR (500 MHz, CD_3_OD): *δ* 0.59 (d, *J* = 6.6 Hz, 3H), 0.81 (d, *J* = 7.4 Hz, 3H), 0.91 (d, *J* = 7.3 Hz, 3H), 0.95 (d, *J* = 6.6 Hz, 3H), 1.02 (d, *J* = 6.9 Hz, 3H), 1.04 (s, 3H), 1.20 (s, 3H), 1.25 (s, 3H), 1.25 (s, 3H), 1.30 (s, 3H), 1.46 (s, 3H), 1.78–1.18 (m, 16H), 1.87 (m, 1H), 1.88 (d, *J* = 1.3 Hz, 3H), 1.92 (d, *J* = 1.3 Hz, 3H), 1.98 (m, 1H), 1.99 (s, 3H), 2.33 (s, 3H), 3.05 (m, 1H), 3.09 (m, 1H), 3.18 (s, 3H), 3.34 (m, 1H), 3.44 (m, 1H), 3.50 (m, 1H), 3.52 (m, 1H), 3.78, (d, *J* = 10.5 Hz, 1H), 3.96 (br d, *J* = 11.7, 5.8, 1H), 4.63 (m, 1H), 5.63 (d, *J* = 9.8 Hz, 1H) 6.85, (dd, *J* = 9.7, 1.4 Hz, 1H), 7.10 (1H, s). ^13^C NMR (125 MHz, CD_3_OD): *δ* 8.4, 11.1, 11.7, 12.0, 12.5, 14.7, 15.9, 16.3, 17.3, 18.2, 18.5, 19.3, 24.5, 25.0, 25.2, 25.7, 27.5, 28.0, 28.3, 29.3, 31.8, 31.9, 32.5, 33.6, 34.7, 36.2, 36.7, 36.8, 45.6, 69.7, 71.7, 74.5, 74.9, 75.1, 77.2, 83.8, 85.2, 97.5, 101.3, 105.9, 127.1, 134.2, 135.2, 136.1, 144.5, 144.9, 167.9, 201.7. ESI-MS: [M + Na]^+^ *m*/*z* 837.5.

### 3.6. Cytotoxicity Bioassay Methods

Human glioblastoma U87 and ovarian cancer SKOV3 cell lines were grown in DMEM (Dulbeccos Modified Eagle Medium; Thermo Fisher Scientific, Waltham, MA, USA, cell culture, supplemented with 10% fetal bovine serum (HyClone) and 1% penicillin/streptomycin/l-glutamine, nonessential amino acids, sodium pyruvate, and HEPES. For LD_50_ determination, cells were plated in low density (3000 cells per well, 100 uL of DMEM medium per well) and treated across a titration of marinolide A (**1**) (*w*/*v*) in biologic triplicate. An 8 × 12 96-well plate was used, with each biologic replicate occupying eight wells on one column. The concentration of marinolide A (**1**) in the eight wells followed a 10-fold serial dilution, starting from 0.5 mg/mL. Based on the molecular weight of marinolide A, the concentration was then converted to molar units. Next, the DMEM medium was removed and replaced with 100 μL of test solution. After 48 h treatment, cell viability was determined by following the alamarBlue (Thermo) cell viability protocol. A 10 μL aliquot of alamarBlue solution was added into each well and the plates were placed back into the incubator. Plates were observed every 30 min until the wells showed differential color gradients, which typically occurred after 90 min of incubation. Using a microplate reader, the intensity of fluorescence at 590 nm was converted to numeric values. The numeric values of wells were divided by the mean value of wells that were treated with the least concentrated marinolide A solution from the biologic triplicate. The quotients were considered as the percentage survival of cells. Marinolide A (**1**) showed weak inhibition against SK-OV-3 drug-resistant ovarian cancer with LD_50_ = 0.079 mM, and against U87 glioblastoma/astrocytoma with an LD_50_ = 0.078 mM.

## 4. Conclusions

In this study, two new 24- and 26-membered bacterial macrolactones, marinolides A and B (**1, 2**), have been reported from the cultivation of a taxonomically unique marine bacterial isolate. The full stereostructures of these new molecules were assigned by comprehensive use of genomic and bioinformatic data coupled with confirmation using extensive NMR analysis and data from a crystalline derivative (**1a**) of marinolide A. As in the past, and here as well, we are careful not to over interpret bioinformatic predictions and to confirm predictions by comprehensive NMR analyses. It is quite remarkable, however, that 13 methine stereogenic centers and 3 olefinic bonds were correctly assigned based entirely upon bioinformatic analysis. While bioactivity was not the focus of this research, we did find that marinolide A (**1**) showed weak activity against SK-OV-3 drug-resistant ovarian cancer with LD_50_ = 0.079 mM, and against U87 glioblastoma/astrocytoma with an LD_50_ = 0.078 mM. Unlike many macrolides, marinolides A and B did not show appreciable antibacterial activities even at relatively high concentrations.

## Data Availability

The marinolide cluster is deposited under the GenBank accession number OL257848. The whole genome shotgun project of AJS-327 has been deposited at DDBJ/ENA/GenBank under the BioProject accession number PRJNA525102. Crystal structure data for the marinolide A derivative **1a** is available in the CCDC/FIZ Karlsruhe depository under deposition number 2033847.

## Supplementary Materials

The following supporting information can be downloaded at: https://www.mdpi.com/article/10.3390/md21060367/s1: Full NMR spectral data, high resolution mass spectrometry data, X-ray crystal experimental data, and phylogenetic analyses of the marinolide polyketide synthase domains.
