# Supplementary material for: Expanding the Utility of Bioinformatic Data for the Full Stereostructural Assignments of Marinolides A and B, 24- and 26-Membered Macrolactones Produced by a Chemically Exceptional Marine-Derived Bacterium
Source: Mar Drugs. 2023 Jun 20;21(6):367. doi: 10.3390/md21060367 (PMC10301374; doi:10.3390/md21060367)
Supplement: Supplementary file 1 [file marinedrugs-21-00367-s001.zip › marinedrugs-2459613-supplementary.pdf]

Supporting Information

Expanding the Utility of Bioinformatic Data for the Full Stereostructural Assignments of Marinolides A and B, 24- and 26-Membered Macrolactones Produced by a Chemically Exceptional Marine-Derived Bacterium

Min Cheol Kim,[1] Jaclyn M. Winter,[2*] Reiko Cullum,[1] Alexander J. Smith,[1] William Fenical[1,3,4*]

[1]Center for Marine Biotechnology and Biomedicine, Scripps Institution of Oceanography, University of California, San Diego, La Jolla, CA 92093, United States

[2]Department of Pharmacology and Toxicology, University of Utah, Salt Lake City, Utah 84112, United States

[3]Skaggs School of Pharmacy and Pharmaceutical Sciences, University of California, San Diego, La Jolla, CA 92093, United States

[4]Moores Comprehensive Cancer Center, University of California, San Diego, La Jolla, CA 92093, United States

[*]To whom correspondence may be addressed. E-mail: wfenical@ucsd.edu and jaclyn.winter@utah.edu

Table of Contents

Supplementary Tables

Table S1. NMR spectroscopic data for marinolides A (**1**) and B (**2**) **S35**
Table S2. 2D COSY and/or TOCSY, HMBC, and HSQC-TOCSY NMR data for marinolide A (**1**) **S37**
Table S3. Tabulated 2D ROESY data for marinolide A (**1**) **S39**
Table S4. Annotation of the *mld* biosynthetic cluster from Actinomycete AJS-327 **S40**
Table S5. Crystal data and structure refinement for **1a** **S45**
Table S6. Atomic coordinates (x 104) and equivalent isotropic displacement parameters (Å2x 103) for **1a** **S46**
Table S7. Bond lengths [Å] and angles [°] for **1a** **S51**
Table S8. Anisotropic displacement parameters (Å2x 103) for **1a** **S68**
Table S9. Hydrogen coordinates (x 104) and isotropic displacement parameters (Å2x 10 3) for **1a** **S73**

Supplemental Figures [NMR spectra recorded at 500 MHz (^{1}H) and 125 MHz (^{13}C)]

Figure S1. ^{1}H NMR spectrum of marinolide A (**1**) in benzene-d_6 **S3**
Figure S2. ^{13}C NMR spectrum of marinolide A (**1**) in benzene-d_6 **S4**
Figure S3. COSY NMR spectrum of marinolide A (**1**) in benzene-d_6 **S5**
Figure S4. HSQC NMR spectrum of marinolide A (**1**) in benzene-d_6 **S6**
Figure S5. HMBC NMR spectrum of marinolide A (**1**) in benzene-d_6 **S7**
Figure S6. Extended HMBC NMR spectrum of marinolide A (**1**) in benzene-d_6 **S8**
Figure S7. ROESY NMR spectrum of marinolide A (**1**) in benzene-d_6 **S9**
Figure S8. TOCSY NMR spectrum of marinolide A (**1**) in benzene-d_6 **S10**
Figure S9. HETLOC NMR spectrum of marinolide A (**1**) in benzene-d_6 **S11**
Figure S10. HSQC-TOCSY NMR spectrum of marinolide A (**1**) in benzene-d_6 **S12**
Figure S11. ^{1}H NMR spectrum of marinolide A (**1**) in CD_3OD **S13**
Figure S12. ^{13}C NMR spectrum of marinolide A (**1**) in CD_3OD **S14**
Figure S13. COSY NMR spectrum of marinolide A (**1**) in CD_3OD **S15**
Figure S14. HSQC NMR spectrum of marinolide A (**1**) in CD_3OD **S16**
Figure S15. HMBC NMR spectrum of marinolide A (**1**) in CD_3OD **S17**
Figure S16. HR-ESI-TOFMS spectrum of marinolide A (**1**) **S18**
Figure S17. ^{1}H NMR spectrum of marinolide B (**2**) in benzene-d_6 **S19**
Figure S18. ^{13}C NMR spectrum of marinolide B (**2**) in benzene-d_6 **S20**
Figure S19. COSY NMR spectrum of marinolide B (**2**) in benzene-d_6 **S21**
Figure S20. HSQC NMR spectrum of marinolide B (**2**) in benzene-d_6 **S22**
Figure S21. HMBC NMR spectrum of marinolide B (**2**) in benzene-d_6 **S23**
Figure S22. ROESY NMR spectrum of marinolide B (**2**) in benzene-d_6 **S24**
Figure S23. TOCSY NMR spectrum of marinolide B (**2**) in benzene-d_6 **S25**
Figure S24. HETLOC NMR spectrum of marinolide B (**2**) in benzene-d_6 **S26**
Figure S25. HSQC-TOCSY NMR spectrum of marinolide B (**2**) in benzene-d_6 **S27**
Figure S26. HR-ESI-TOFMS spectrum of marinolide B (**2**). **S28**
Figure S27. ^{1}H NMR spectrum of bis-acetonide (**1a**) in benzene-d_6. **S29**
Figure S28. ^{13}C NMR spectrum of bis-acetonide (**1a**) in benzene-d_6. **S30**
Figure S29. COSY NMR spectrum of bis-acetonide (**1a**) in benzene-d_6. **S31**
Figure S30. HSQC NMR spectrum of bis-acetonide (**1a**) in benzene-d_6. **S32**
Figure S31. HMBC NMR spectrum of bis-acetonide (**1a**) in benzene-d_6. **S33**
Figure S32. ROESY NMR spectrum of bis-acetonide (**1a**) in benzene-d_6. **S34**
Figure S33. *mld* biosynthetic cluster from *Streptomyces* sp. AJS-327 **S40**
Figure S34. Sequence alignment of key acyltransferase domain residues in the *mld* polyketide synthases **S41**
Figure S35. Sequence alignment of key ketoreductase domain residues in the *mld* polyketide synthases **S42**
Figure S36. Sequence alignment of key dehydratase domain residues in the *mld* polyketide synthases **S43**
Figure S37. Sequence alignment of key enoyl reductase domain residues in the *mld* polyketide synthases **S43**
Figure S38. Sequence alignment of key ketosynthase domain residues in the *mld* polyketide synthases **S44**
CIF File information for the bis-acetonide X-Ray structure **S80**
Supplemental References **S80**

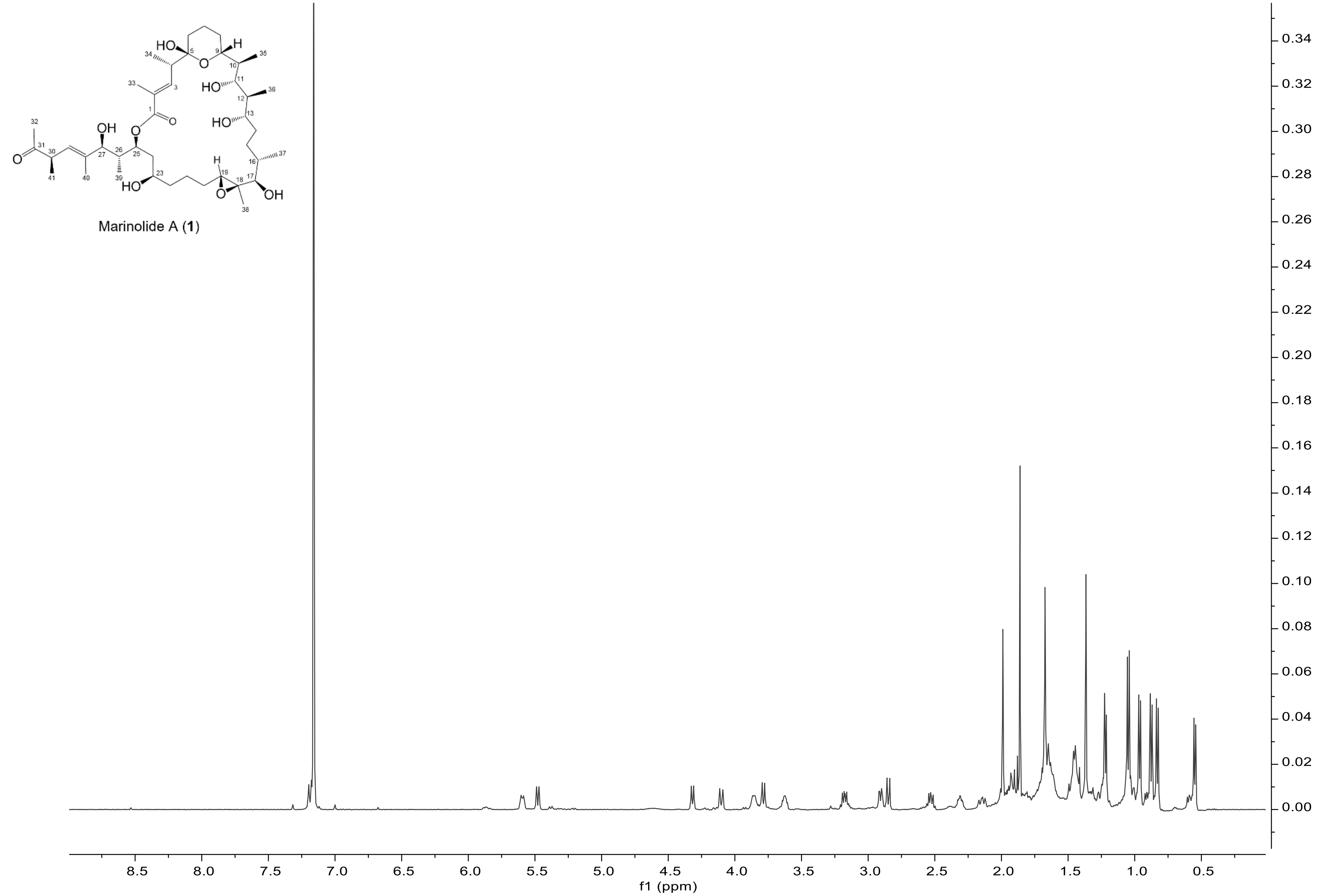

Figure S1. ^{1}H NMR spectrum of marinolide A (**1**) in benzene-d_6.

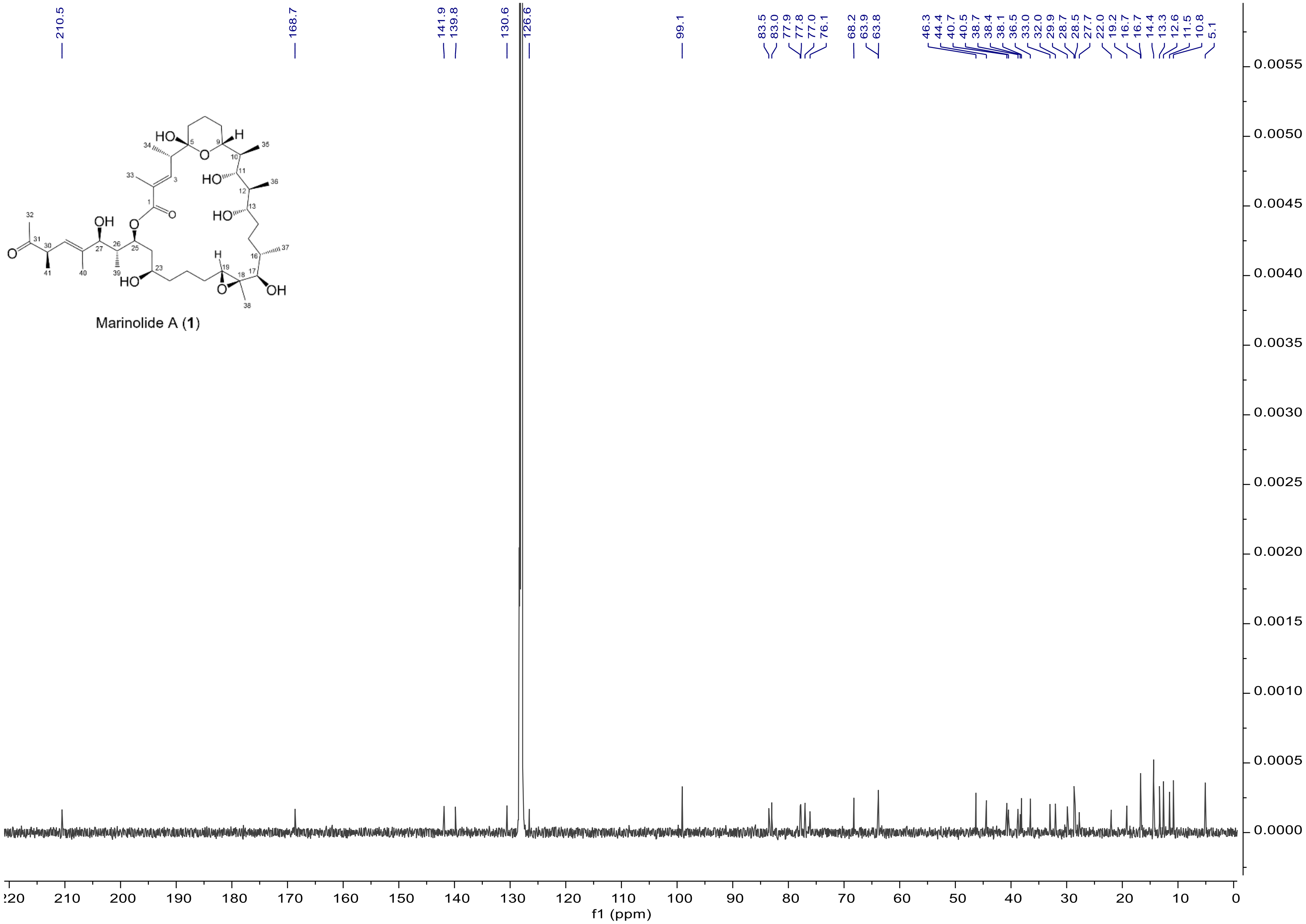

Figure S2. ^{13}C NMR spectrum of marinolide A (**1**) in benzene-d_6.

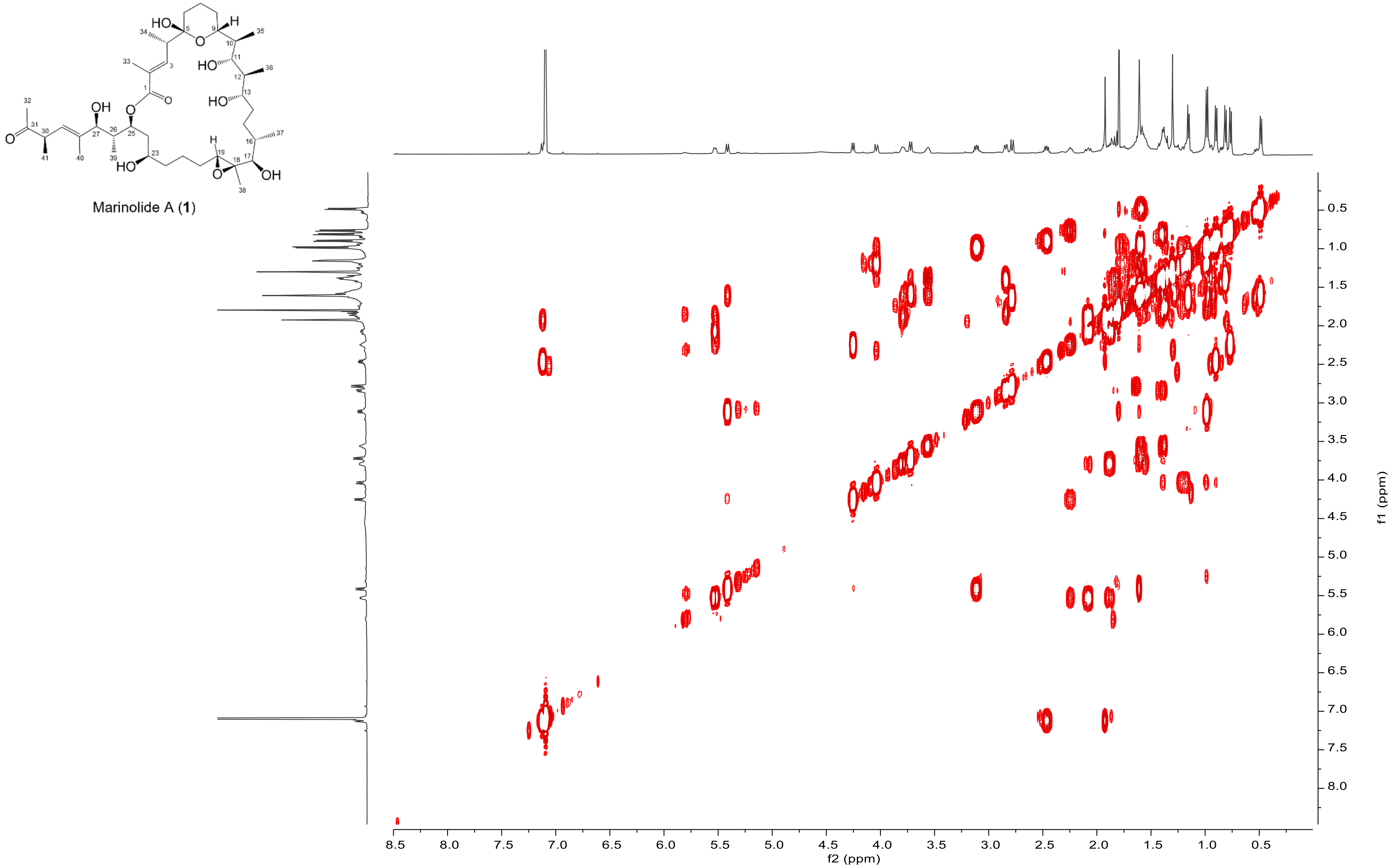

Figure S3. COSY NMR spectrum of marinolide A (**1**) in benzene-d_6.

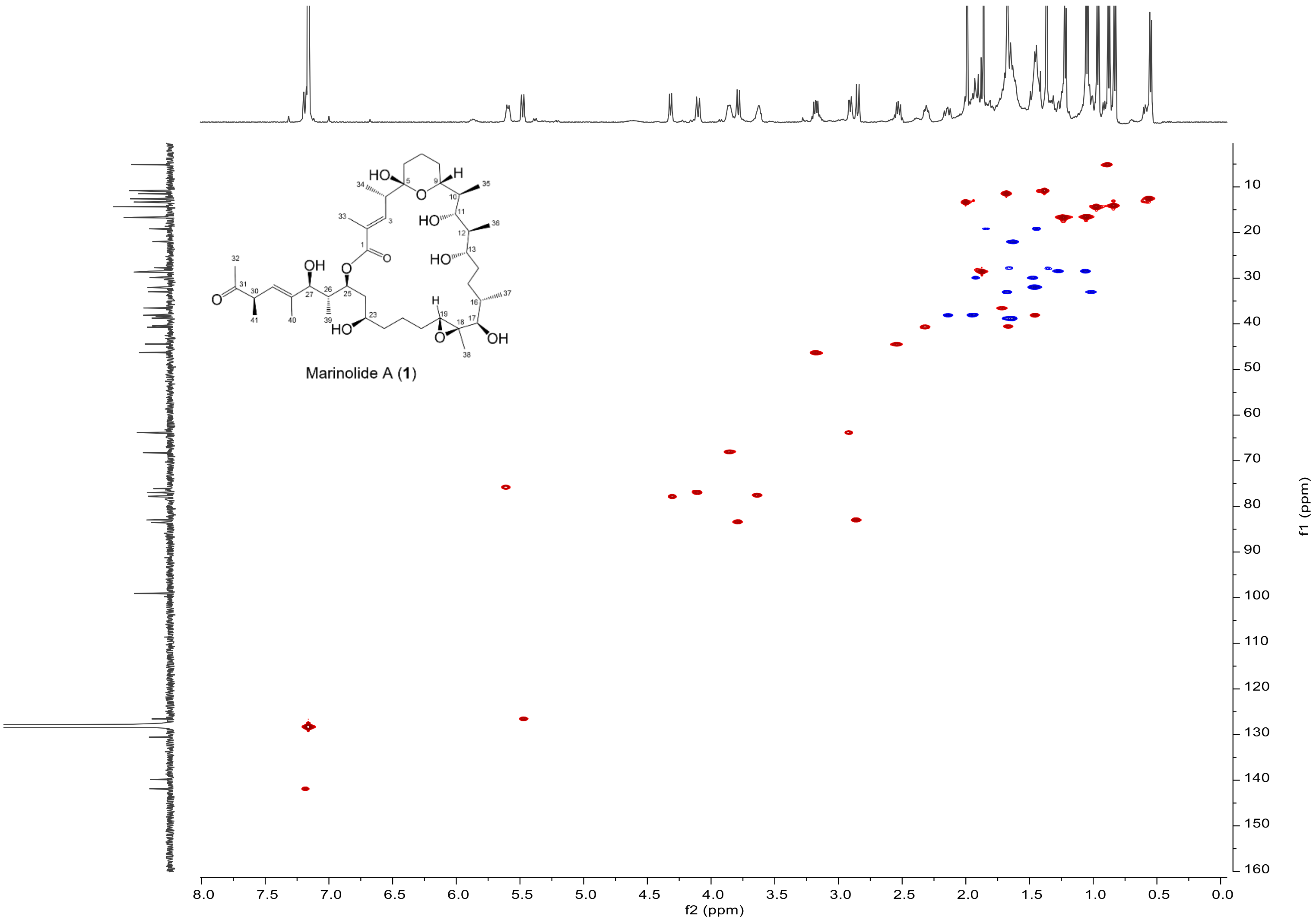

Figure S4. HSQC NMR spectrum of marinolide A (**1**) in benzene-d_6.

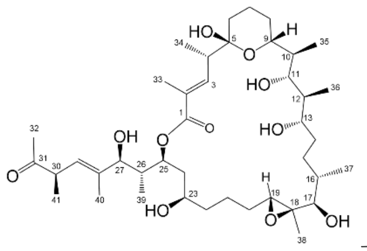

Marinolide A (**1**)

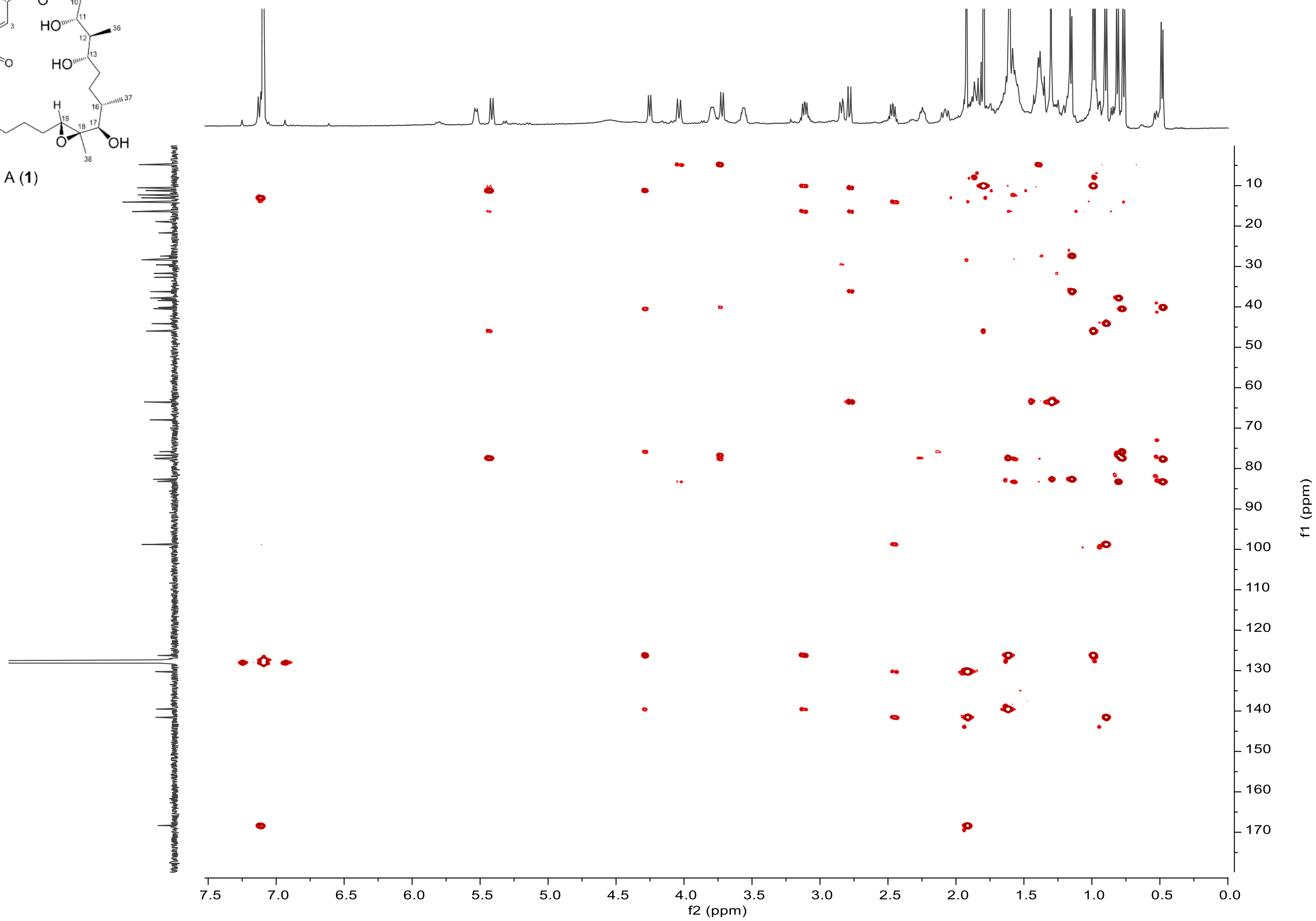

Figure S5. HMBC NMR spectrum of marinolide A (**1**) in benzene-d_6.

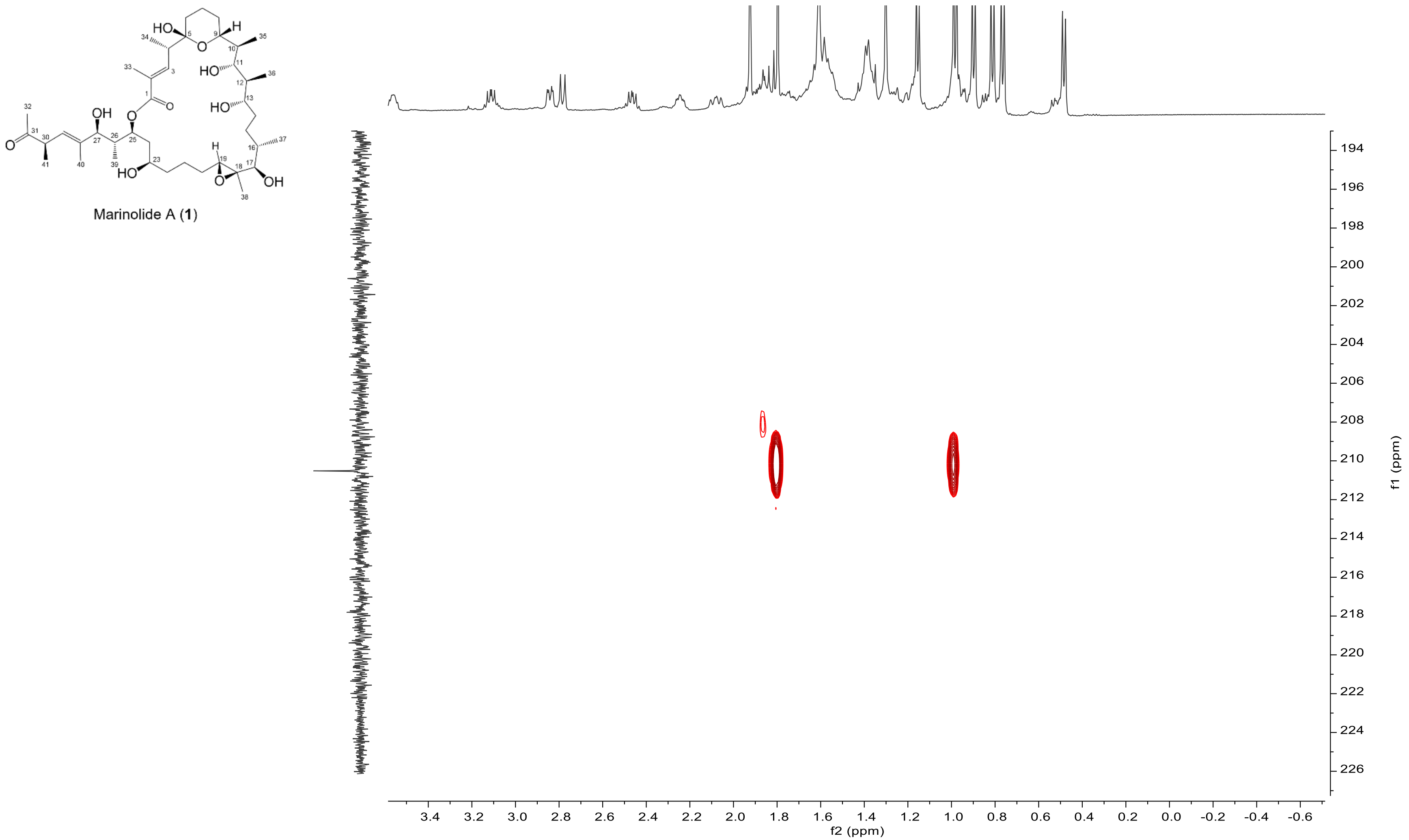

Figure S6. Extended HMBC NMR spectrum of marinolide A (**1**) in benzene-d_6.

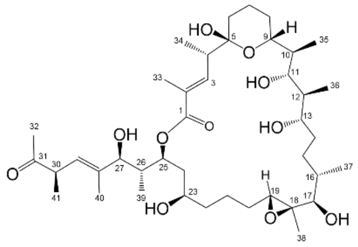

Marinolide A (**1**)

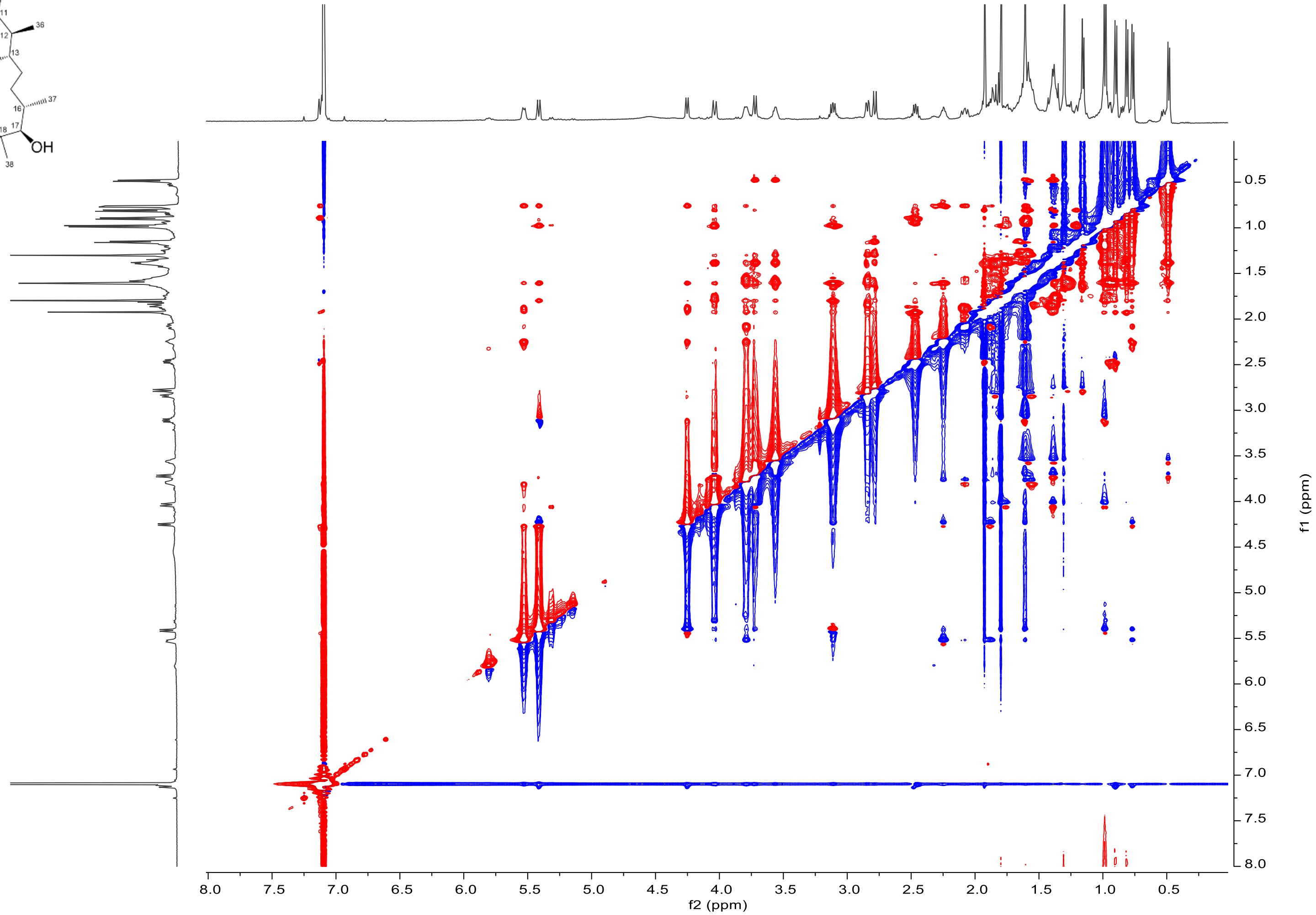

Figure S7. ROESY NMR spectrum of marinolide A (**1**) in benzene-d_6.

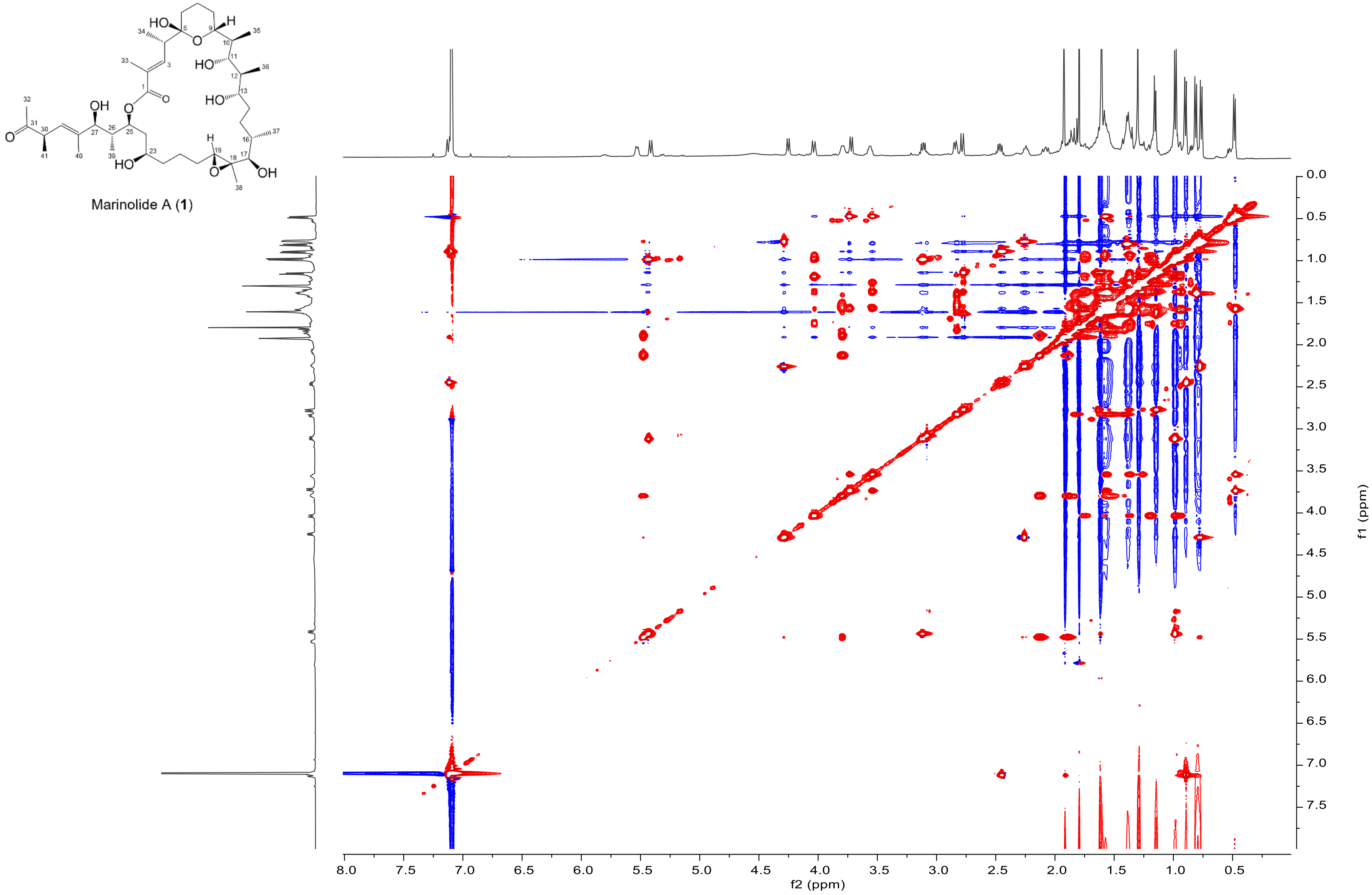

Figure S8. TOCSY NMR spectrum of marinolide A (**1**) in benzene-d_6.

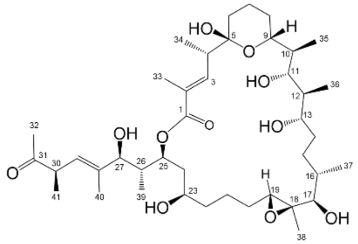

Marinolide A (**1**)

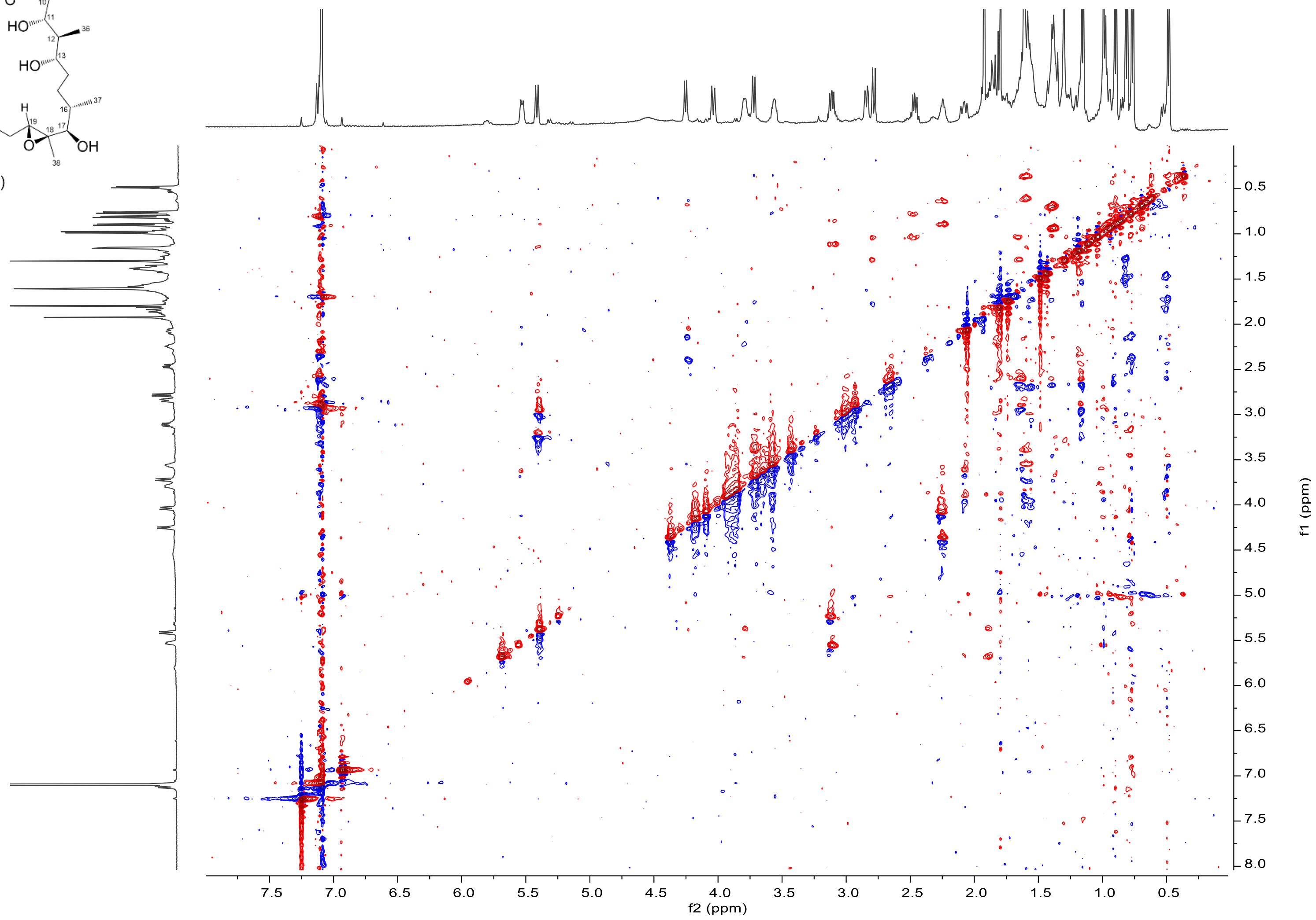

Figure S9. HETLOC NMR spectrum of marinolide A (**1**) in benzene-d_6.

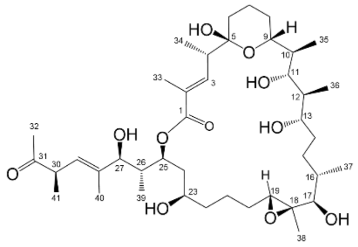

Marinolide A (**1**)

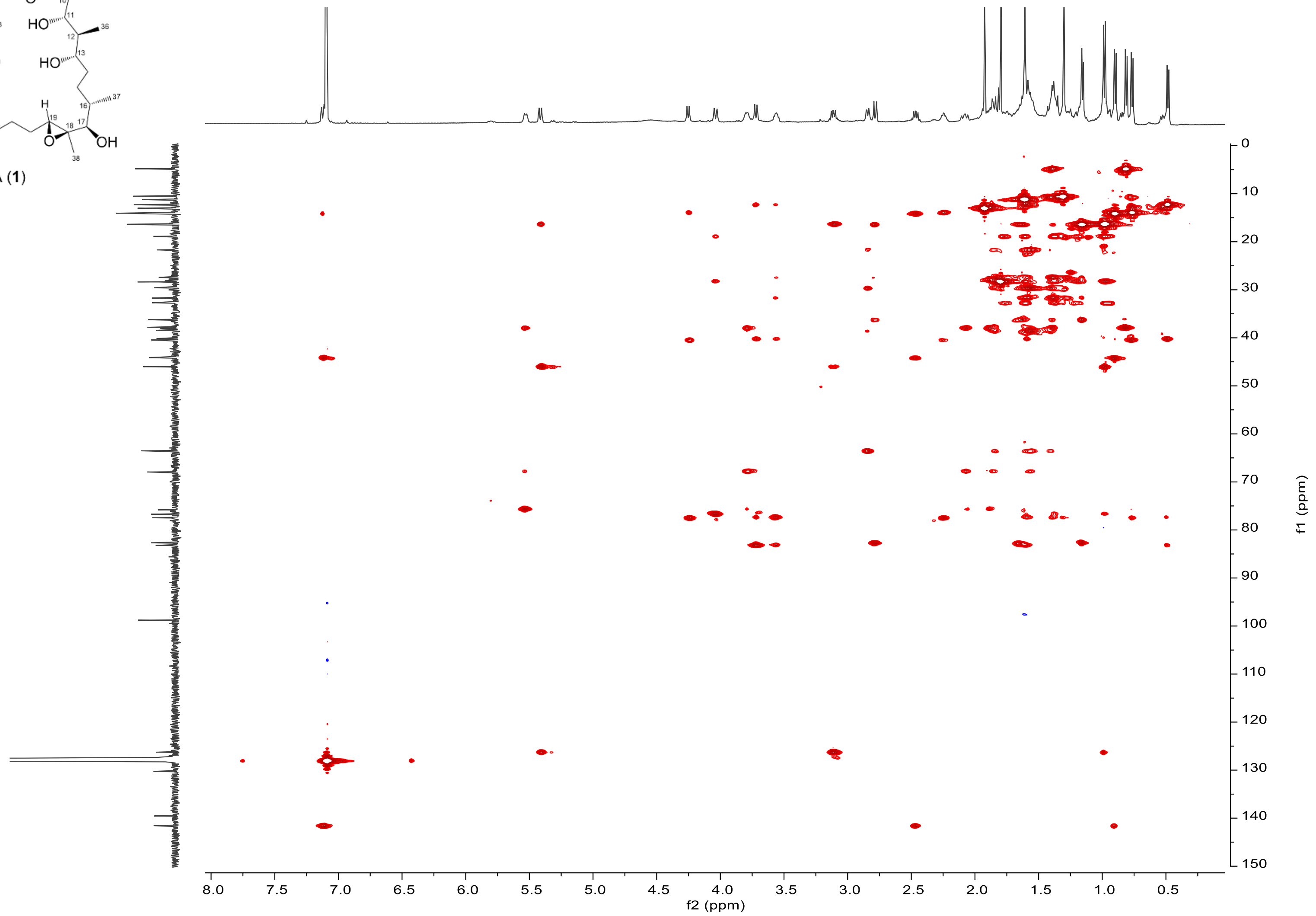

Figure S10. HSQC-TOCSY NMR spectrum of marinolide A (**1**) in benzene-d_6.

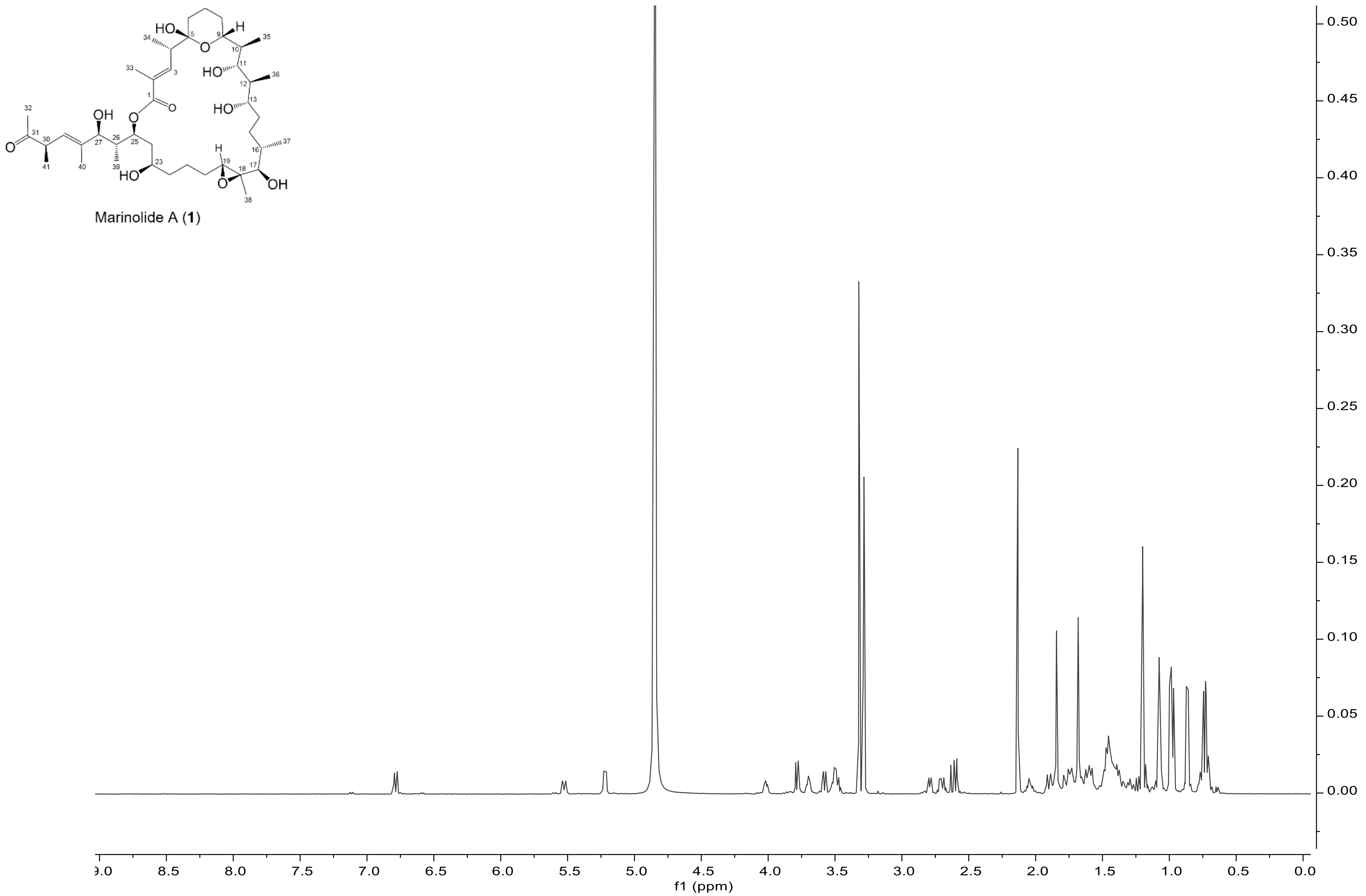

Figure S11. ^{1}H NMR spectrum of marinolide A (**1**) in CD_3OD.

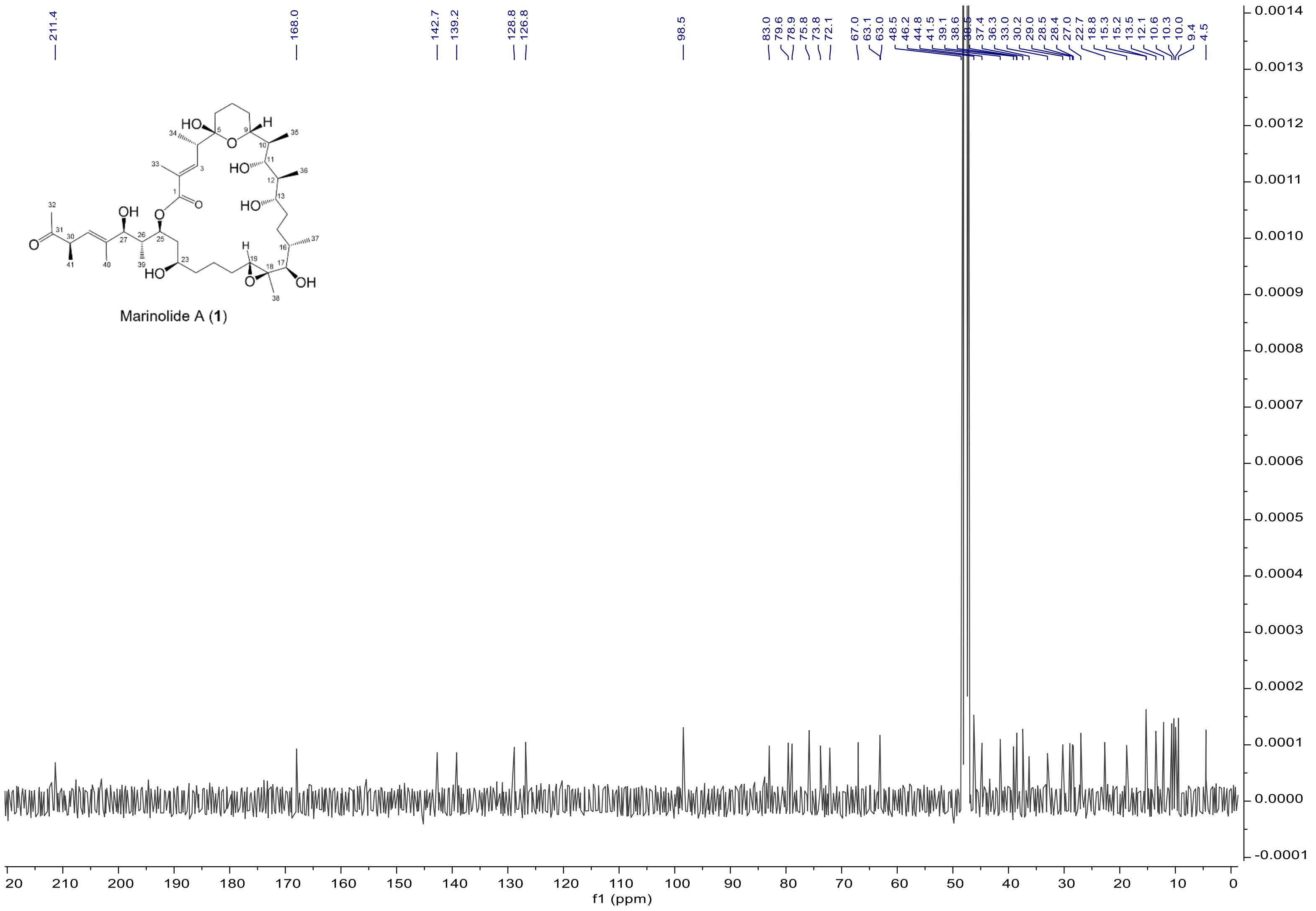

Figure S12. ^{13}C NMR spectrum of marinolide A (**1**) in CD_3OD.

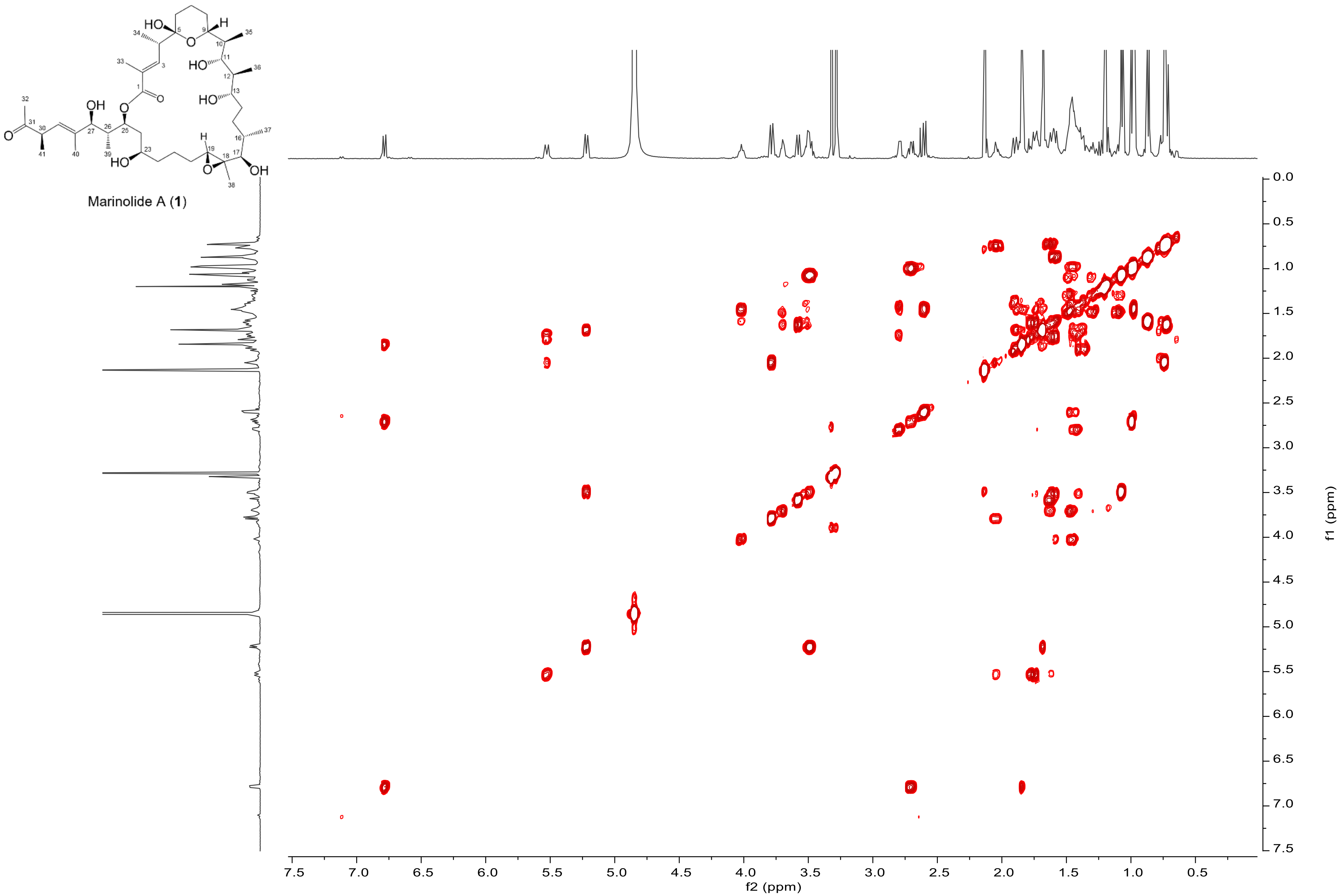

Figure S13. COSY NMR spectrum of marinolide A (**1**) in CD_3OD.

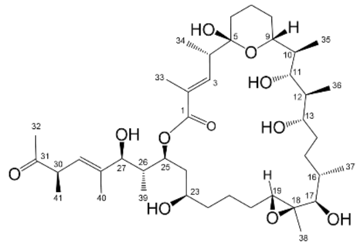

Marinolide A (**1**)

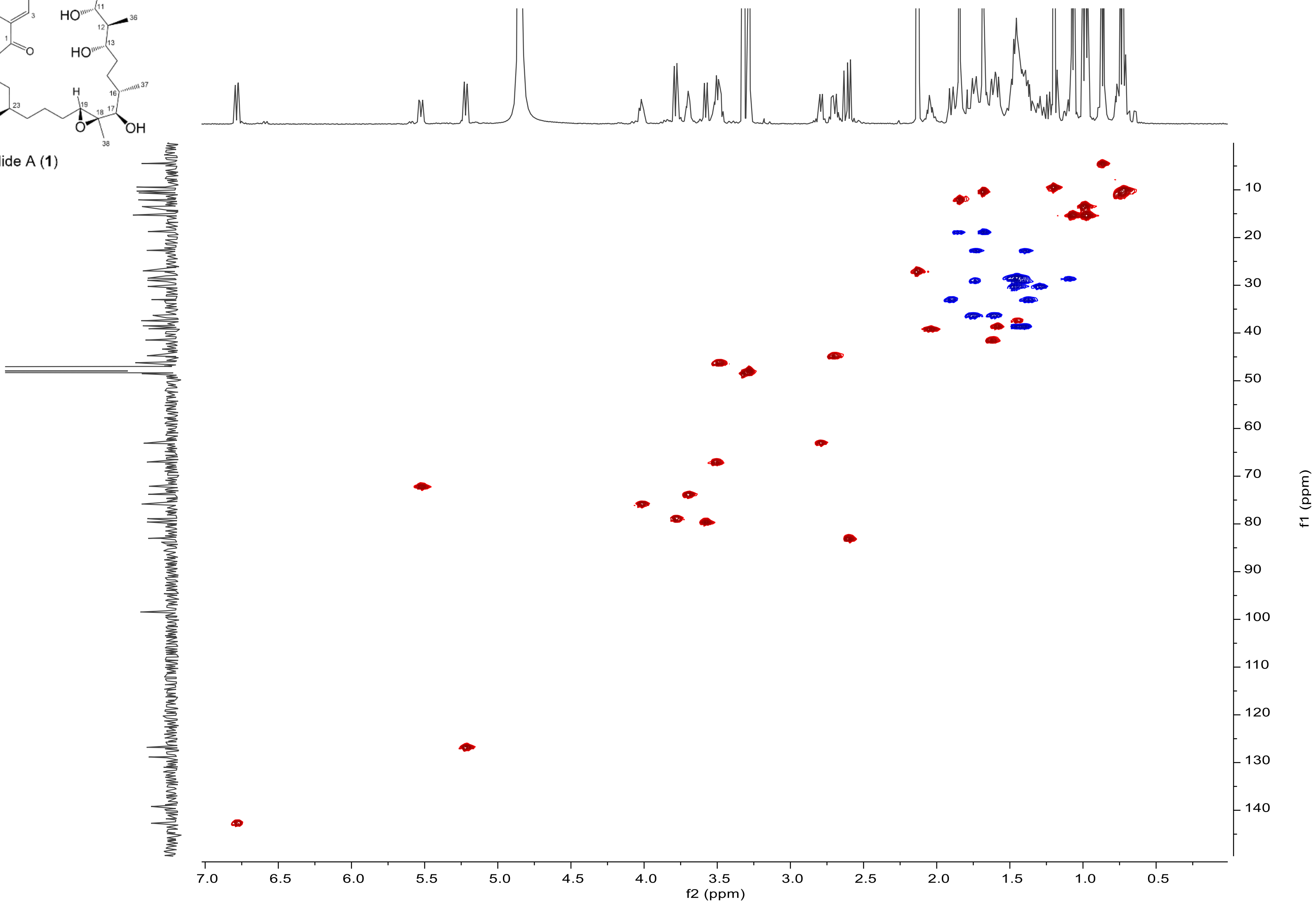

Figure S14. HSQC NMR spectrum of marinolide A (**1**) in CD_3OD.

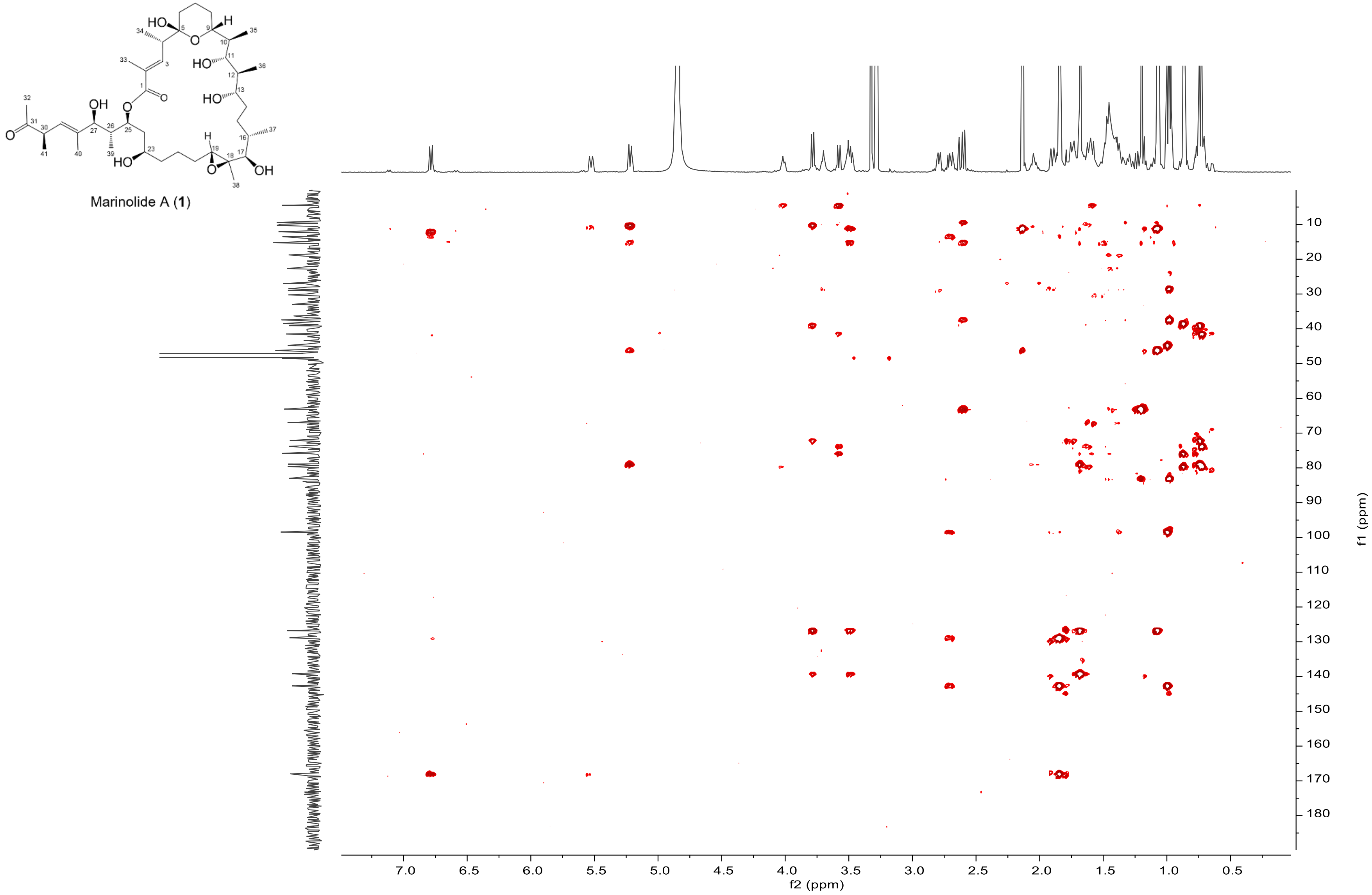

Figure S15. HMBC NMR spectrum of marinolide A (**1**) in CD_3OD.

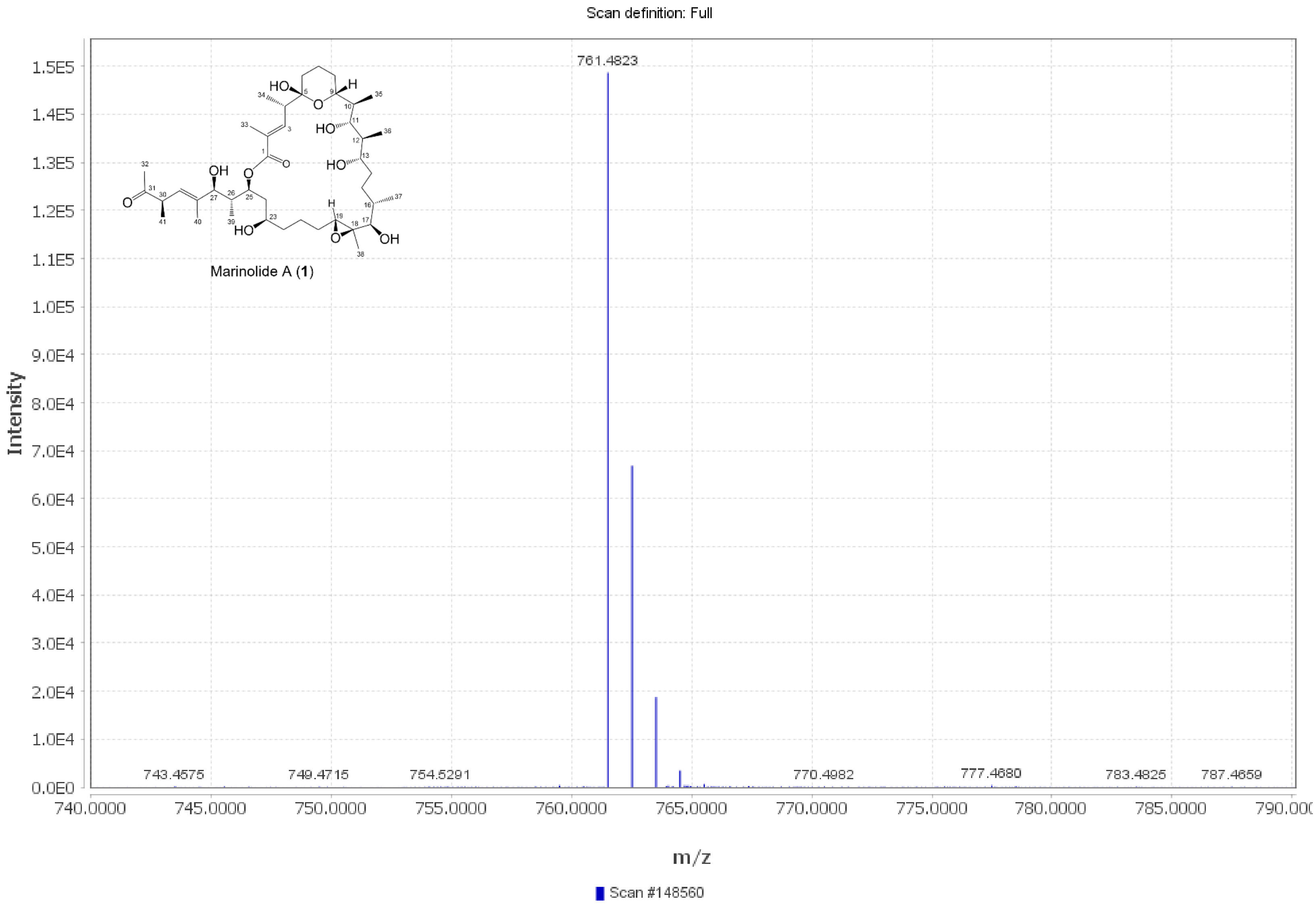

Figure S16. HR-ESI-TOFMS spectrum of marinolide A (**1**).

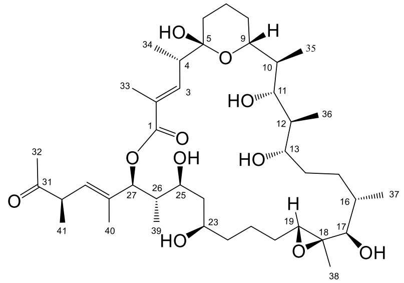

Marinolide B (**2**)

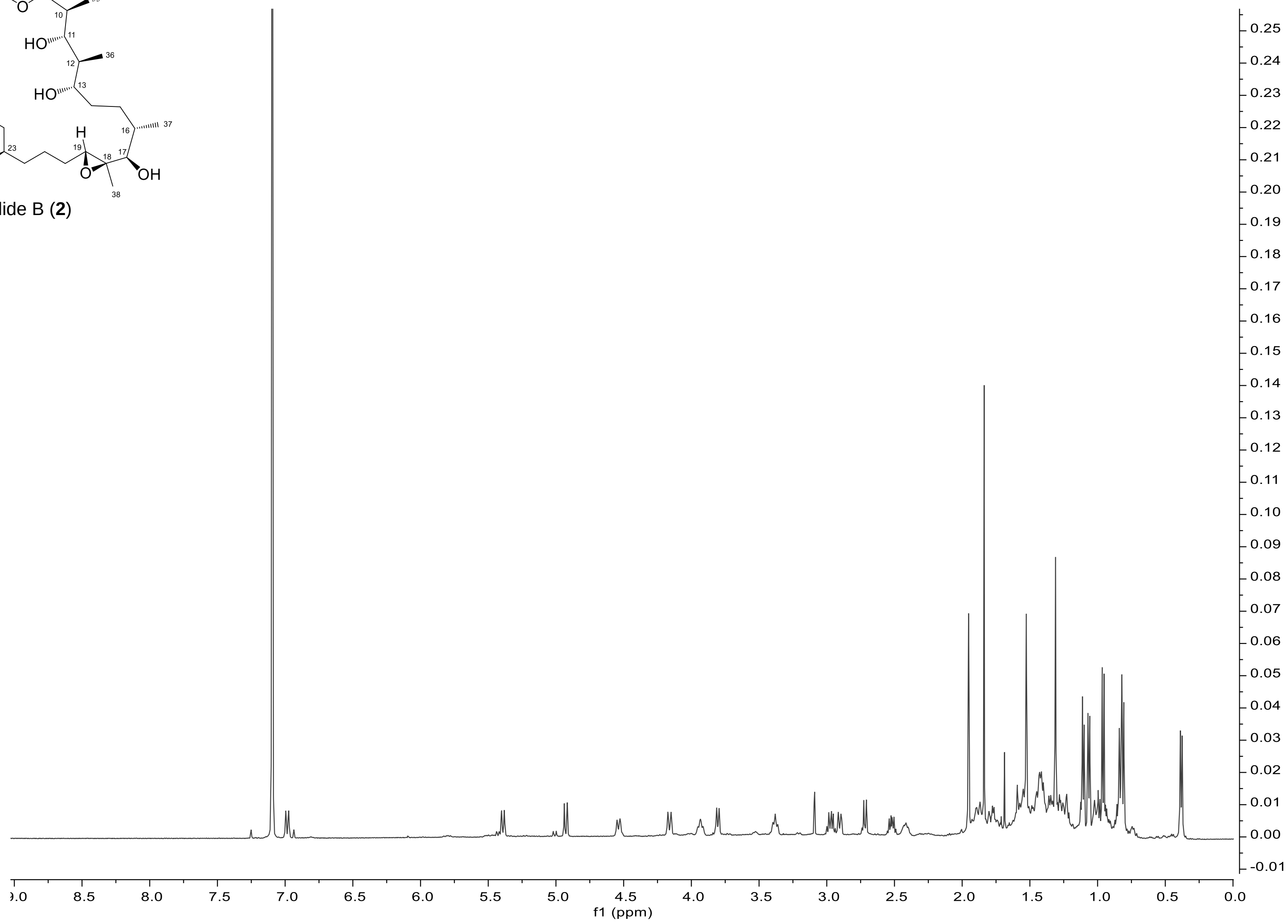

Figure S17. ^{1}H NMR spectrum of marinolide B (**2**) in benzene-d_6.

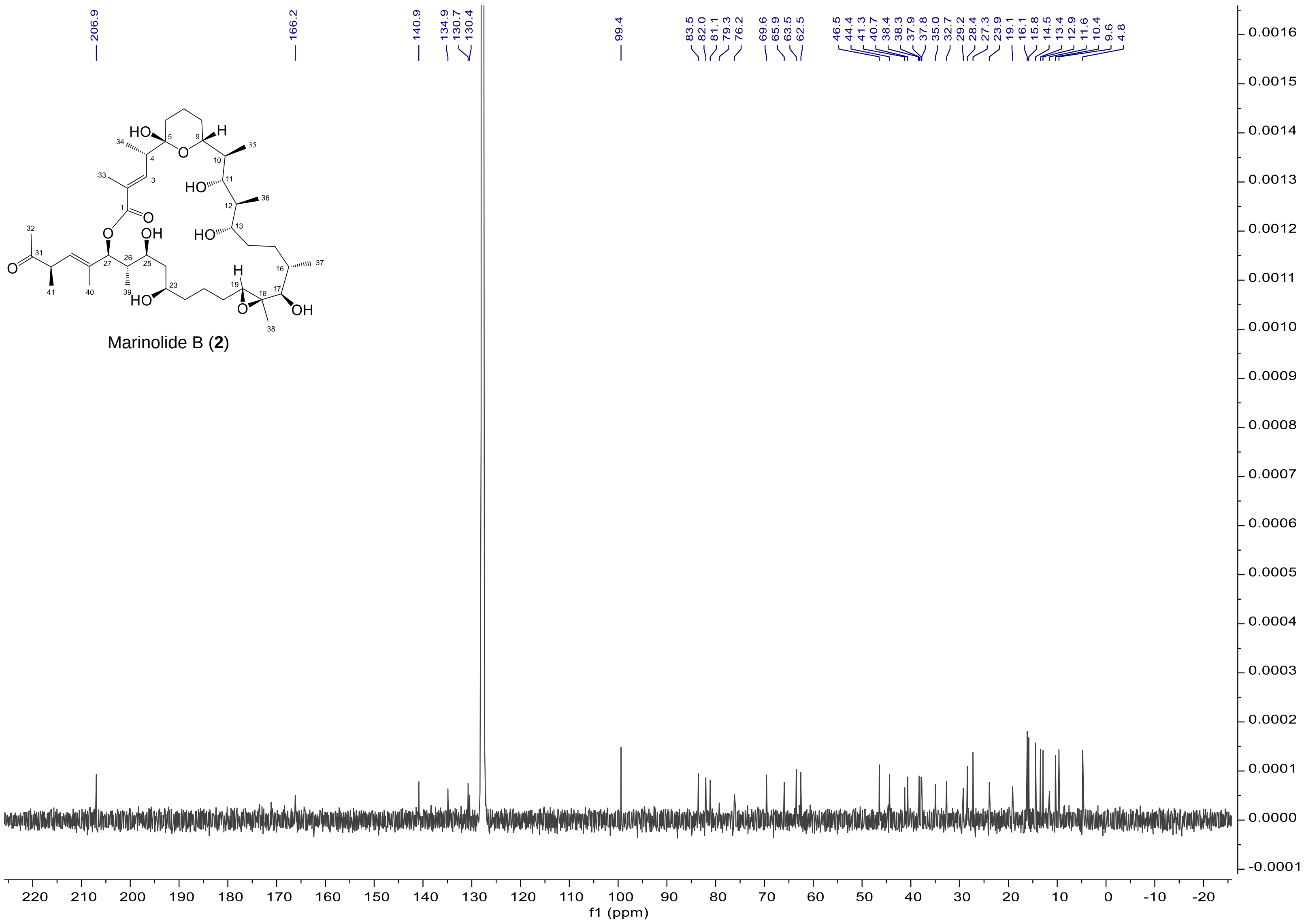

Figure S18. ^{13}C NMR spectrum of marinolide B (**2**) in benzene-d_6.

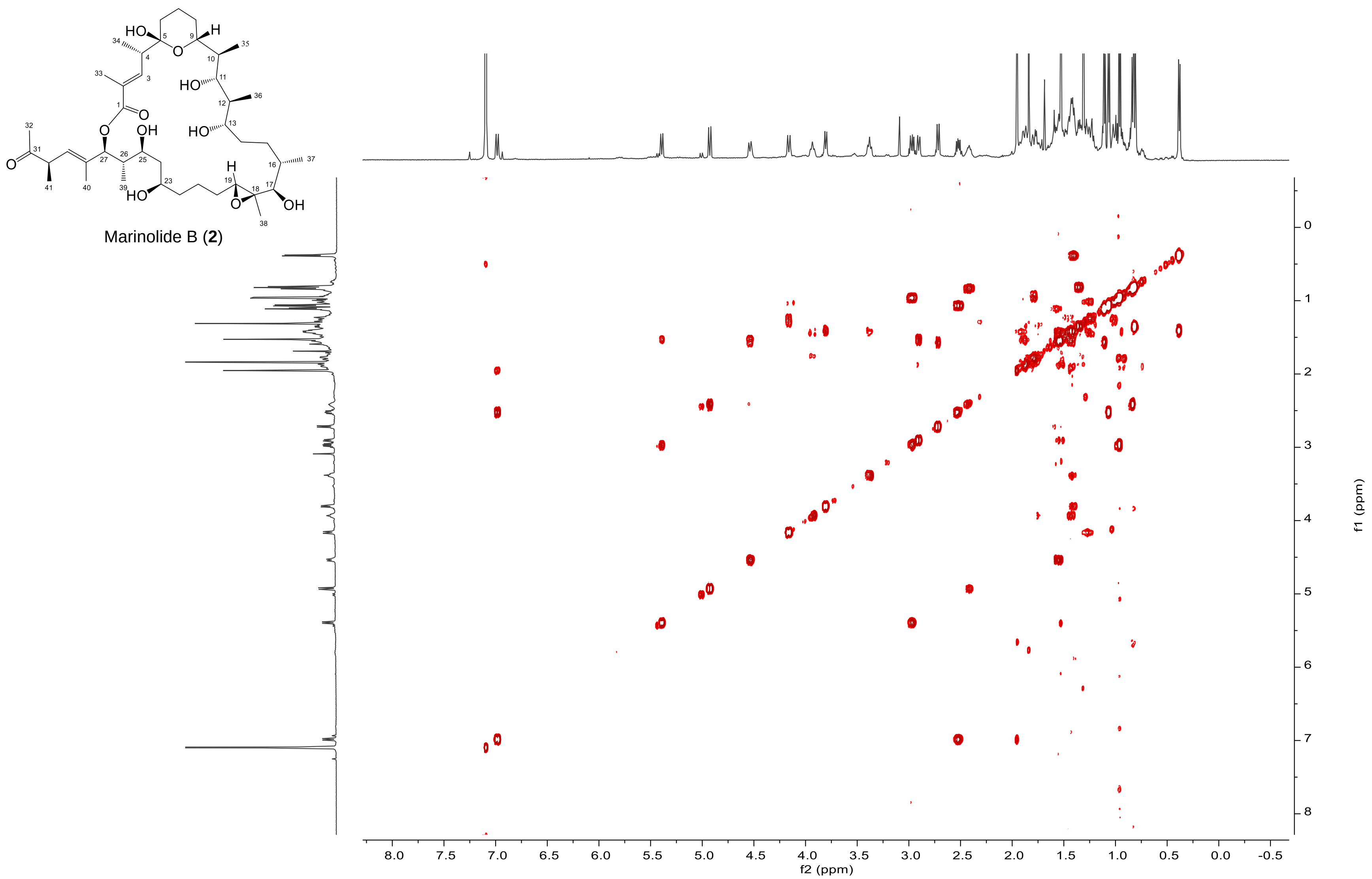

Figure S19. COSY NMR spectrum of marinolide B (**2**) in benzene-d_6.

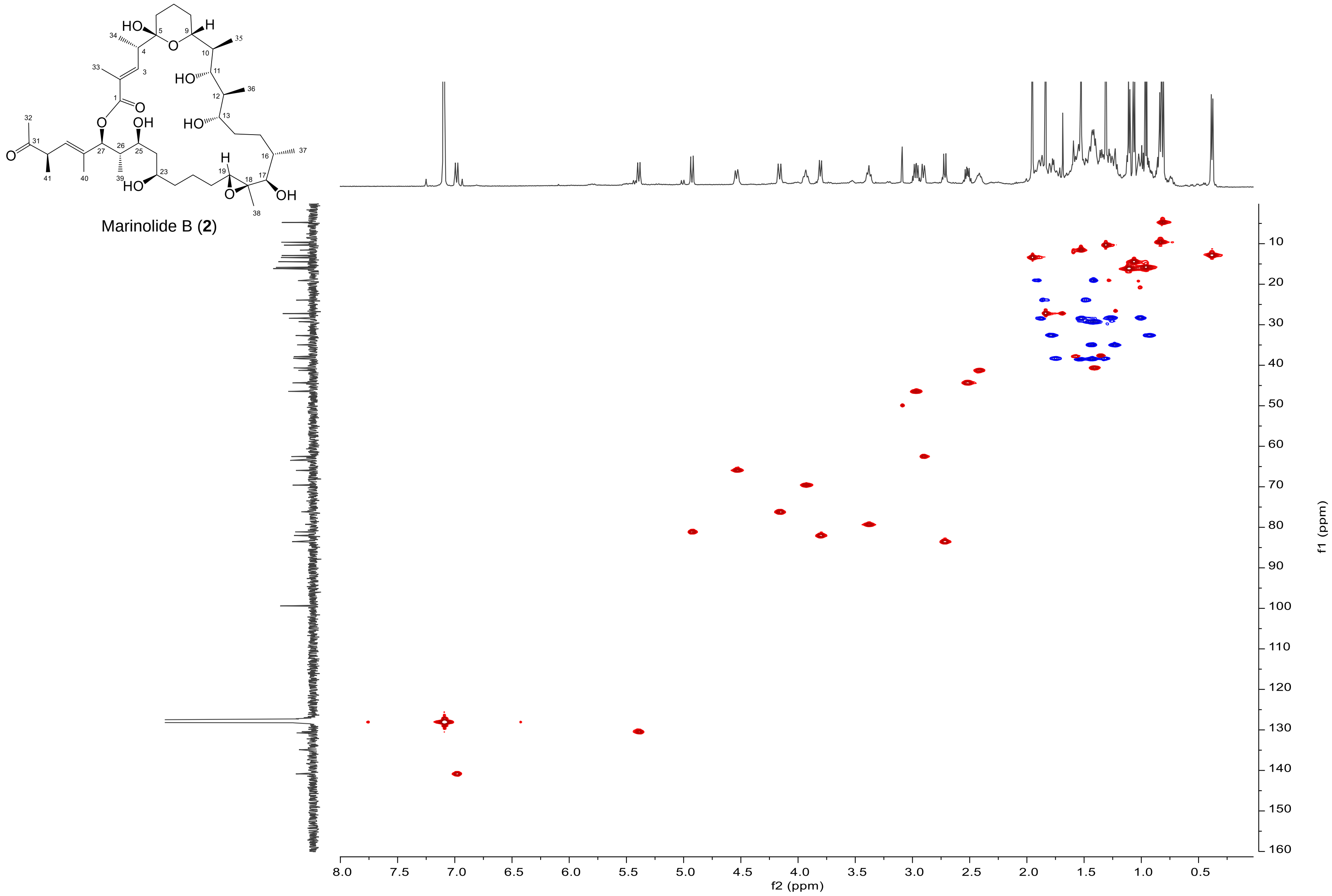

Marinolide B (**2**)

Figure S20. HSQC NMR spectrum of marinolide B (**2**) in benzene-d_6.

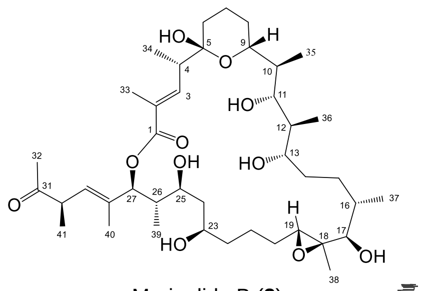

Marinolide B (**2**)

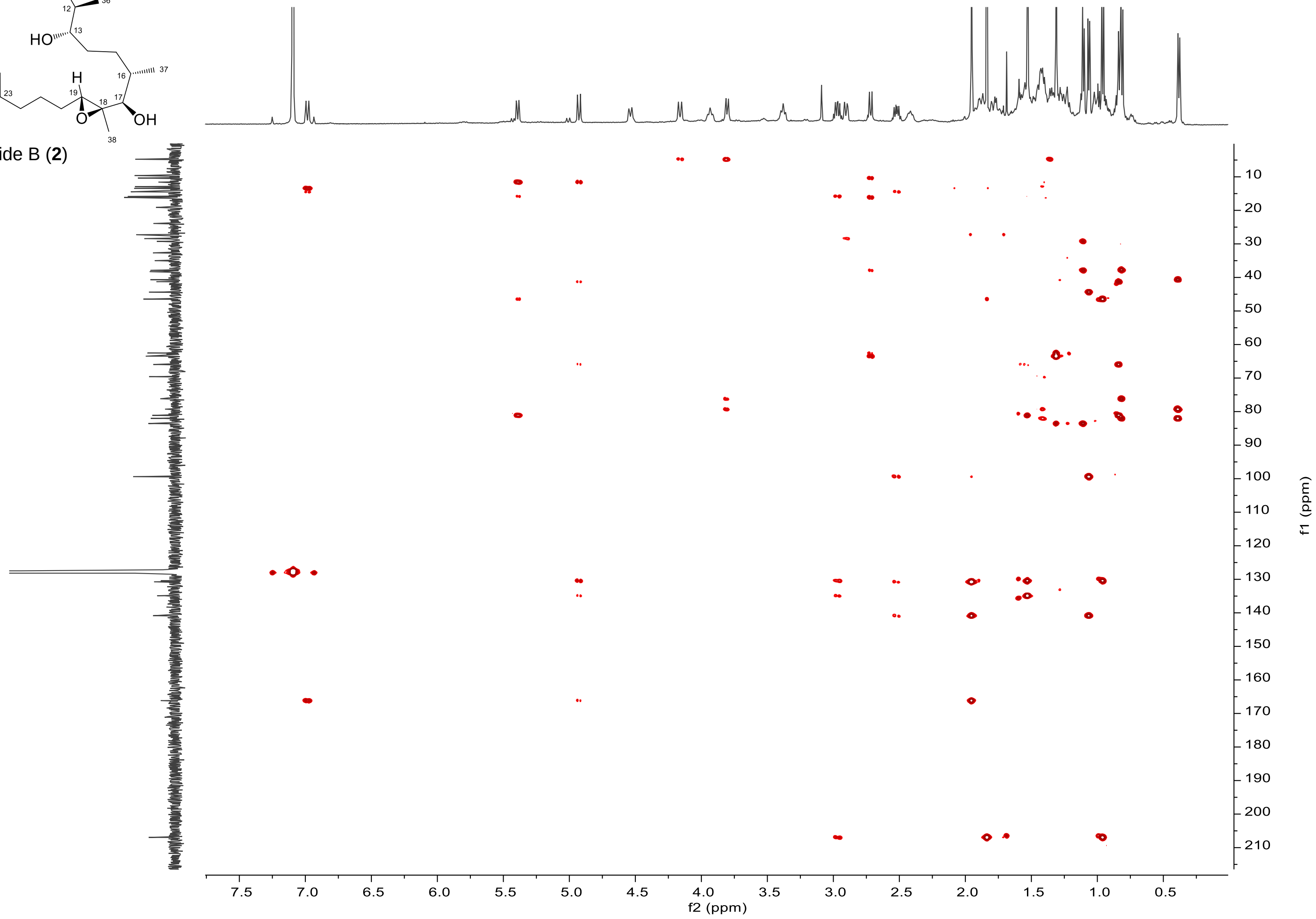

Figure S21. HMBC NMR spectrum of marinolide B (**2**) in benzene-d_6

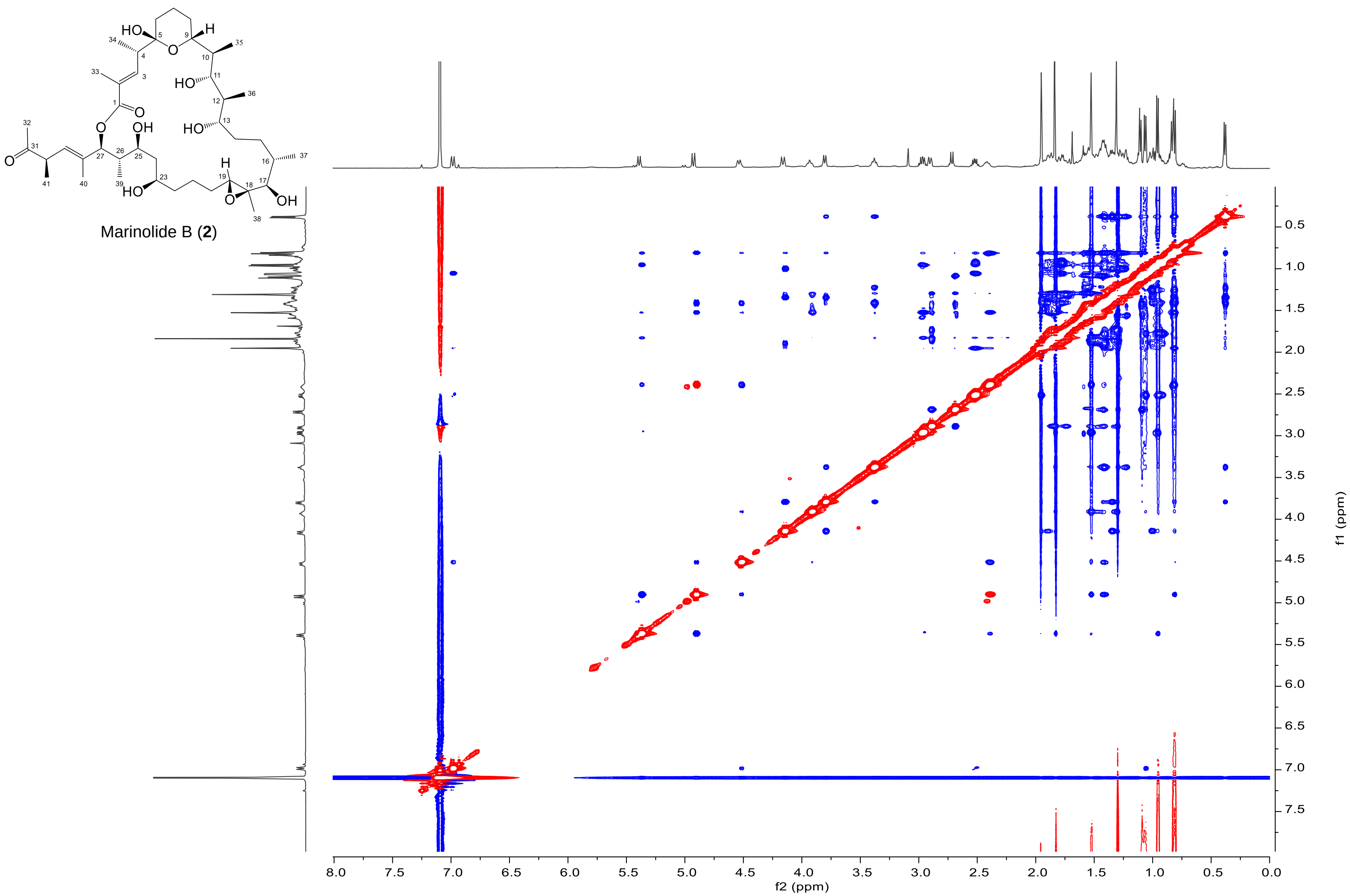

Figure S22. ROESY NMR spectrum of marinolide B (**2**) in benzene-d_6.

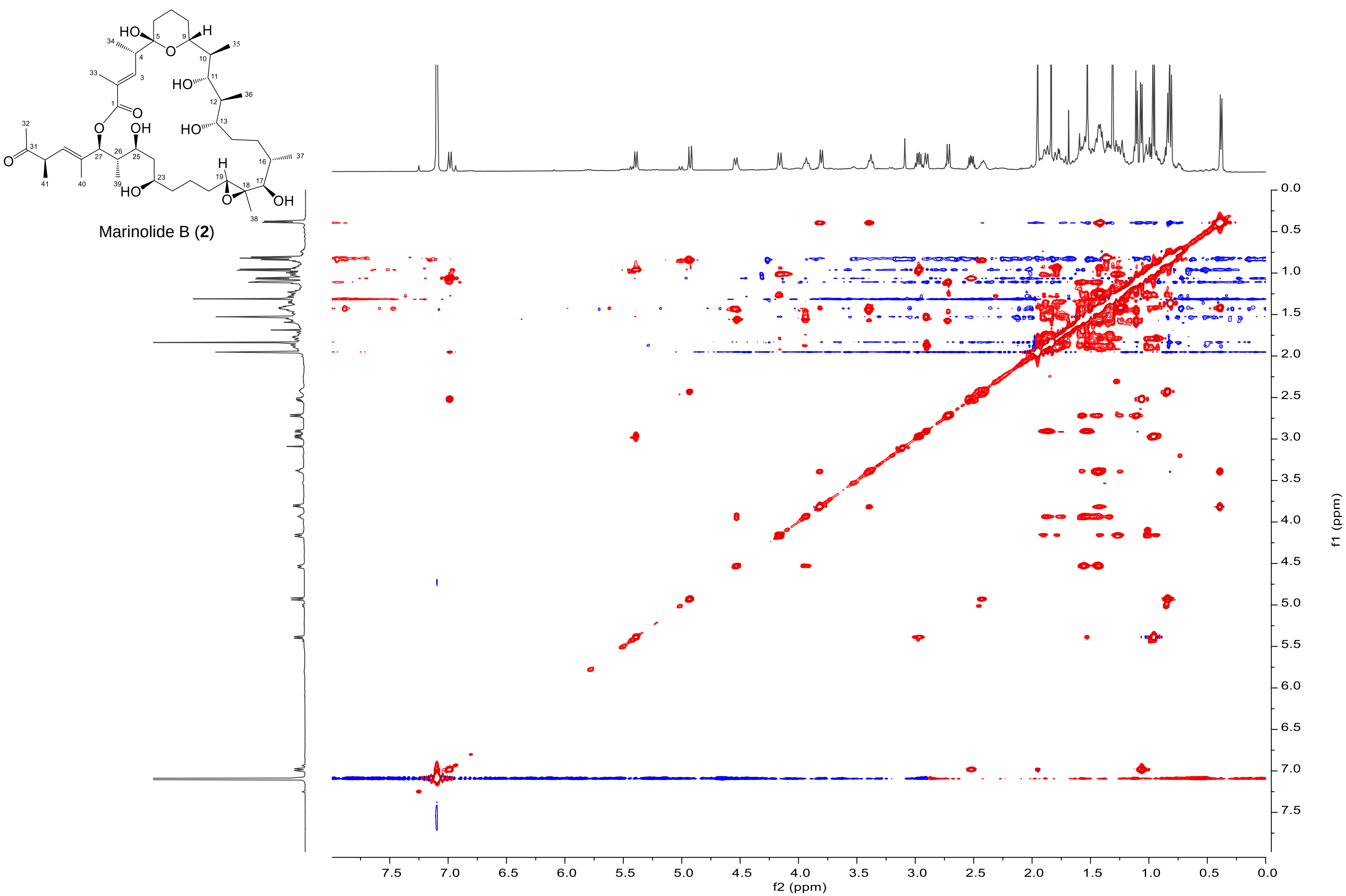

Figure S23. TOCSY NMR spectrum of marinolide B (**2**) in benzene-d_6.

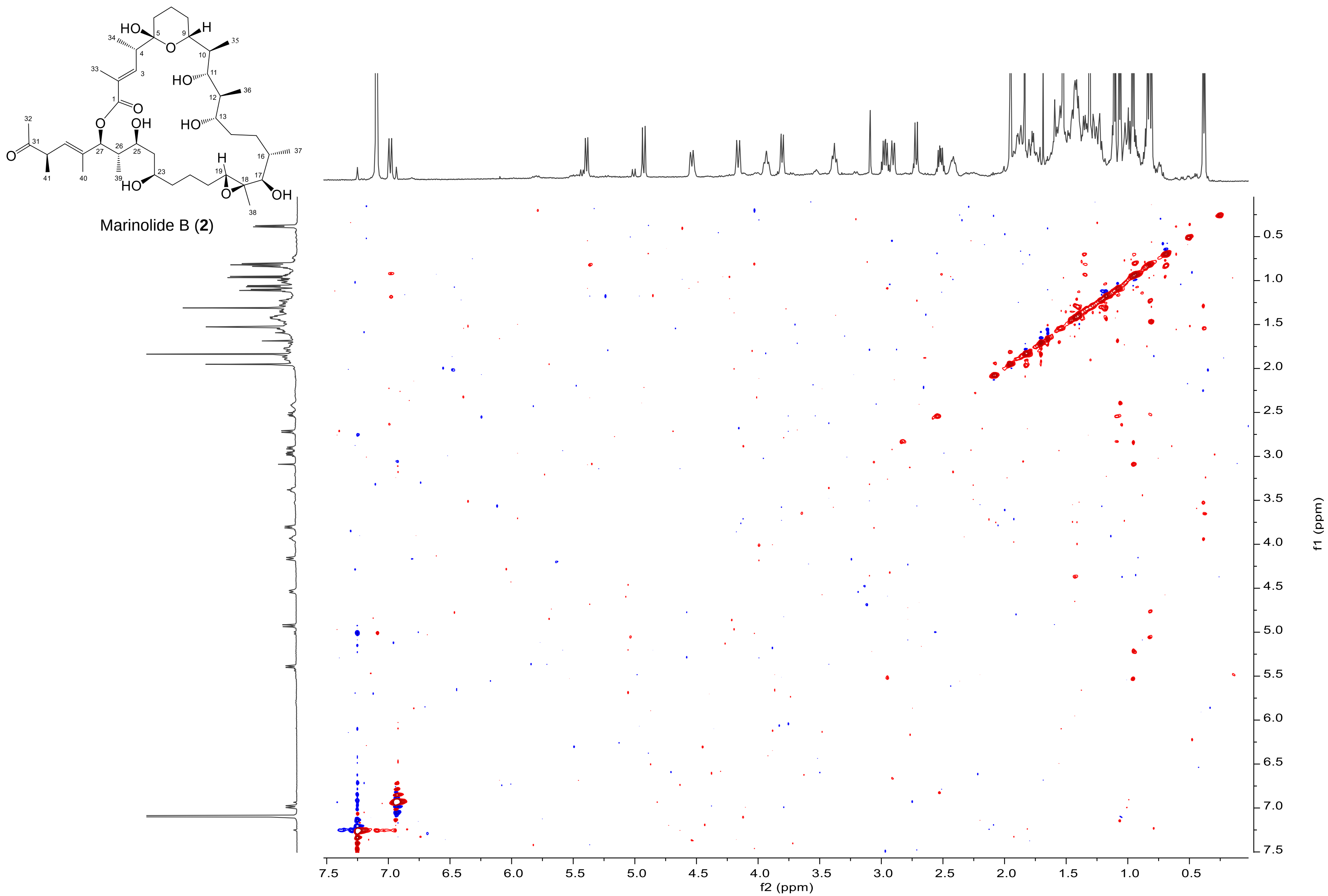

Figure S24. HETLOC NMR spectrum of marinolide B (**2**) in benzene-d_6.

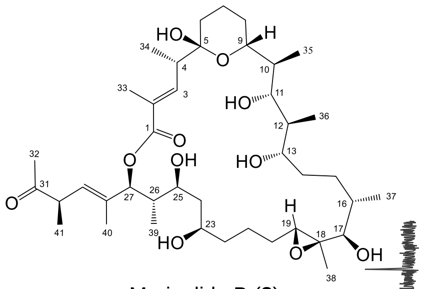

Marinolide B (**2**)

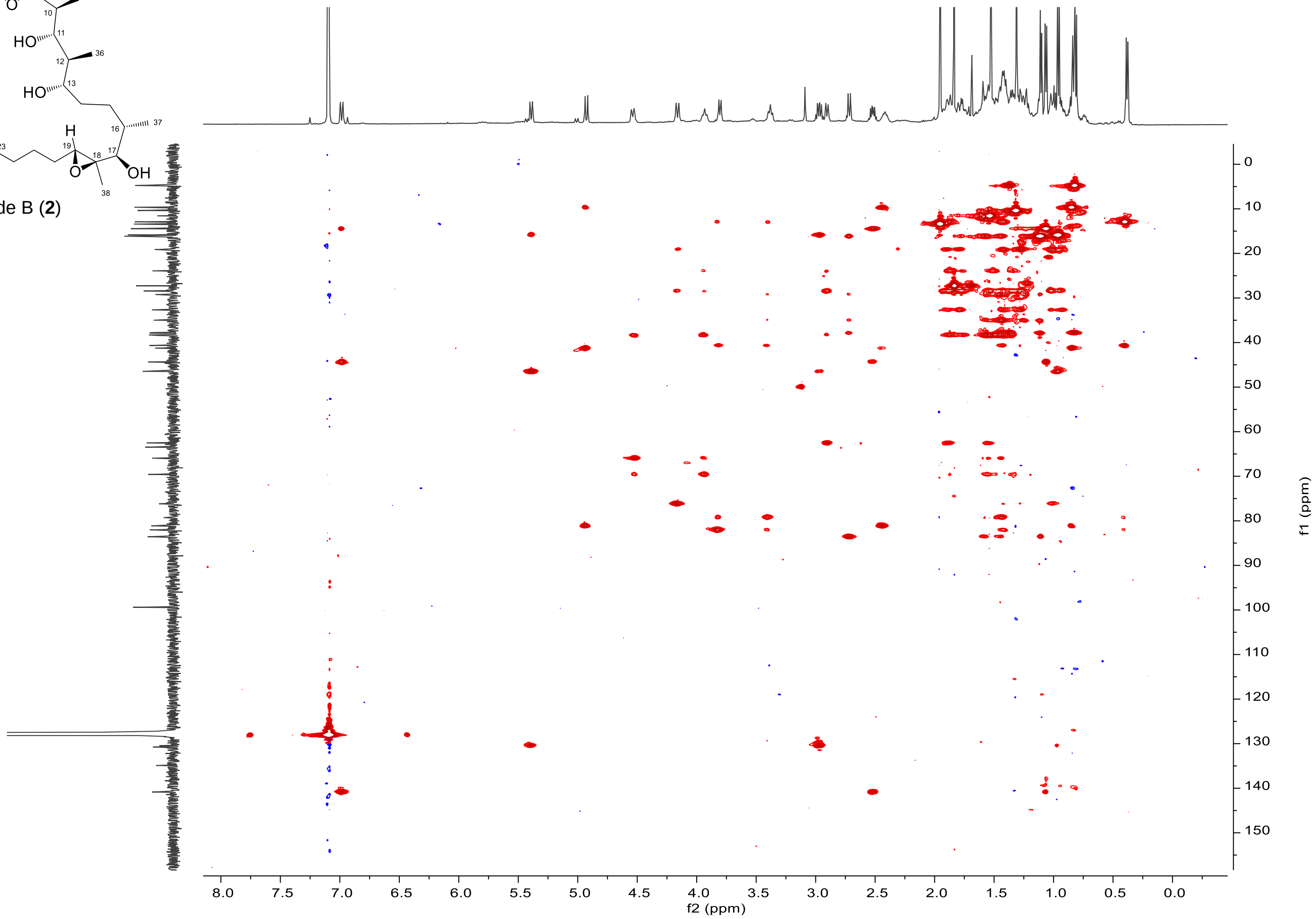

Figure S25. HSQC-TOCSY NMR spectrum of marinolide B (**2**) in benzene-d_6.

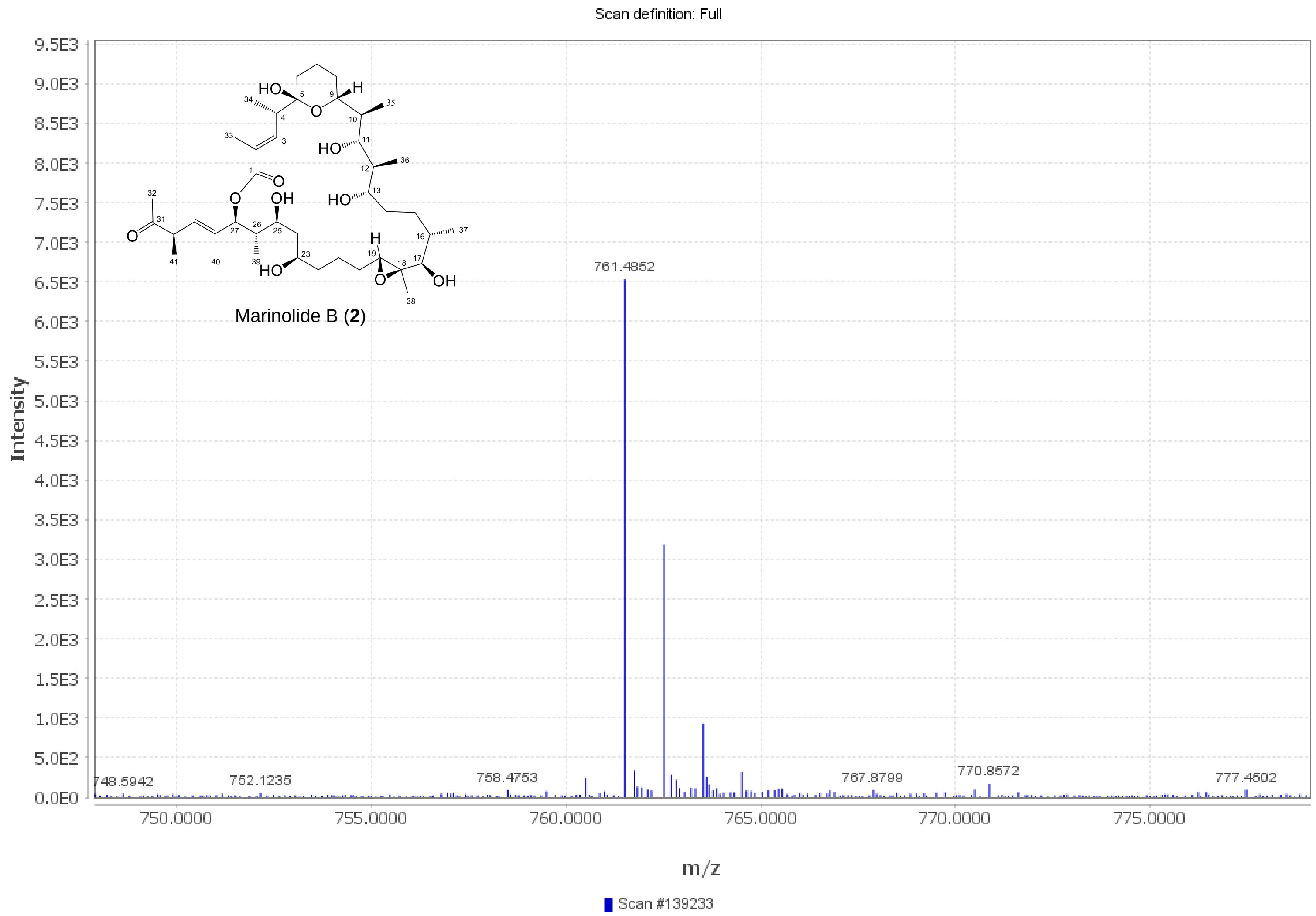

Figure S26. HR-ESI-TOFMS spectrum of marinolide B (**2**).

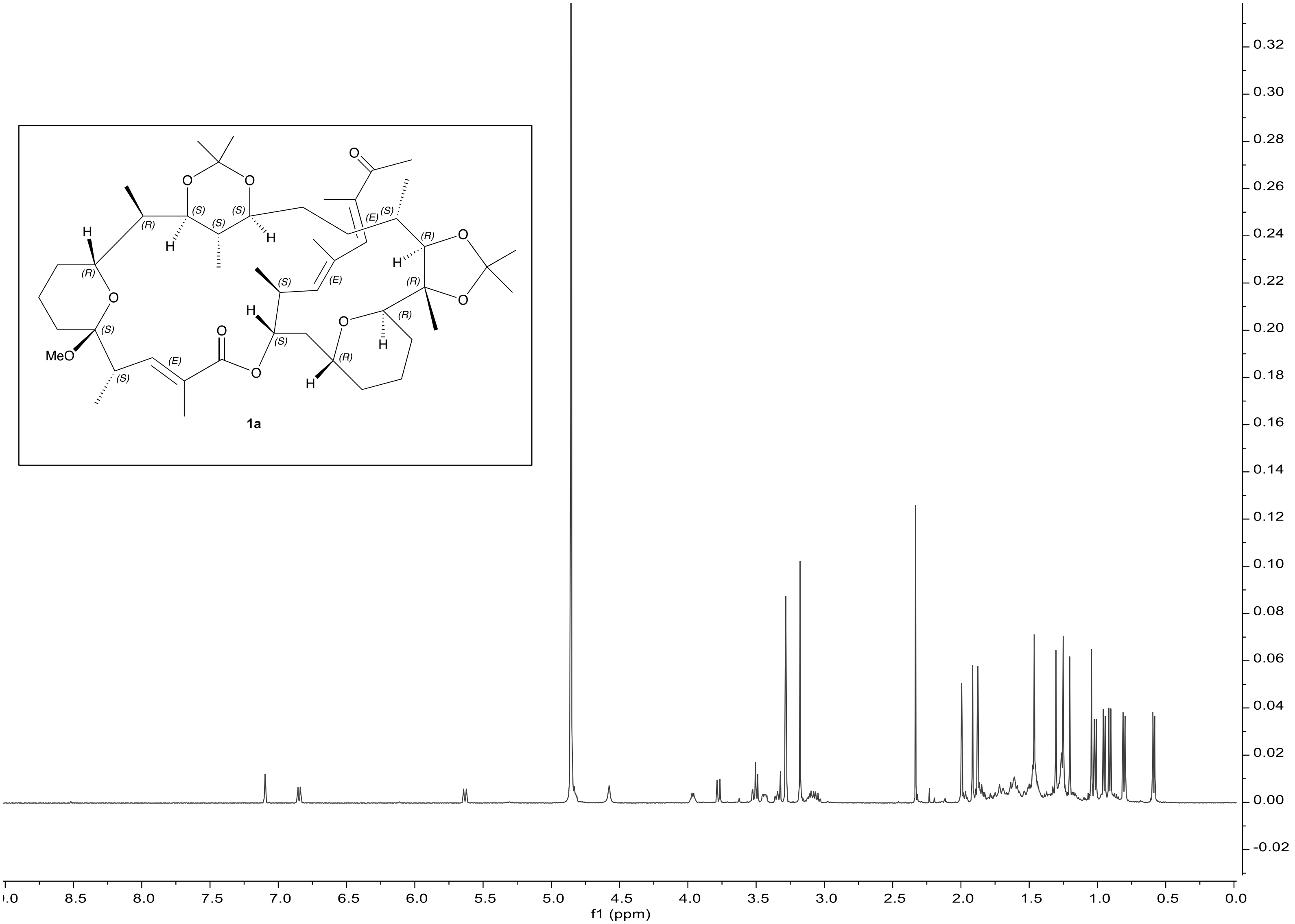

Figure S27. ^{1}H NMR spectrum of *bis*-acetonide (**1a**) in benzene-d_6.

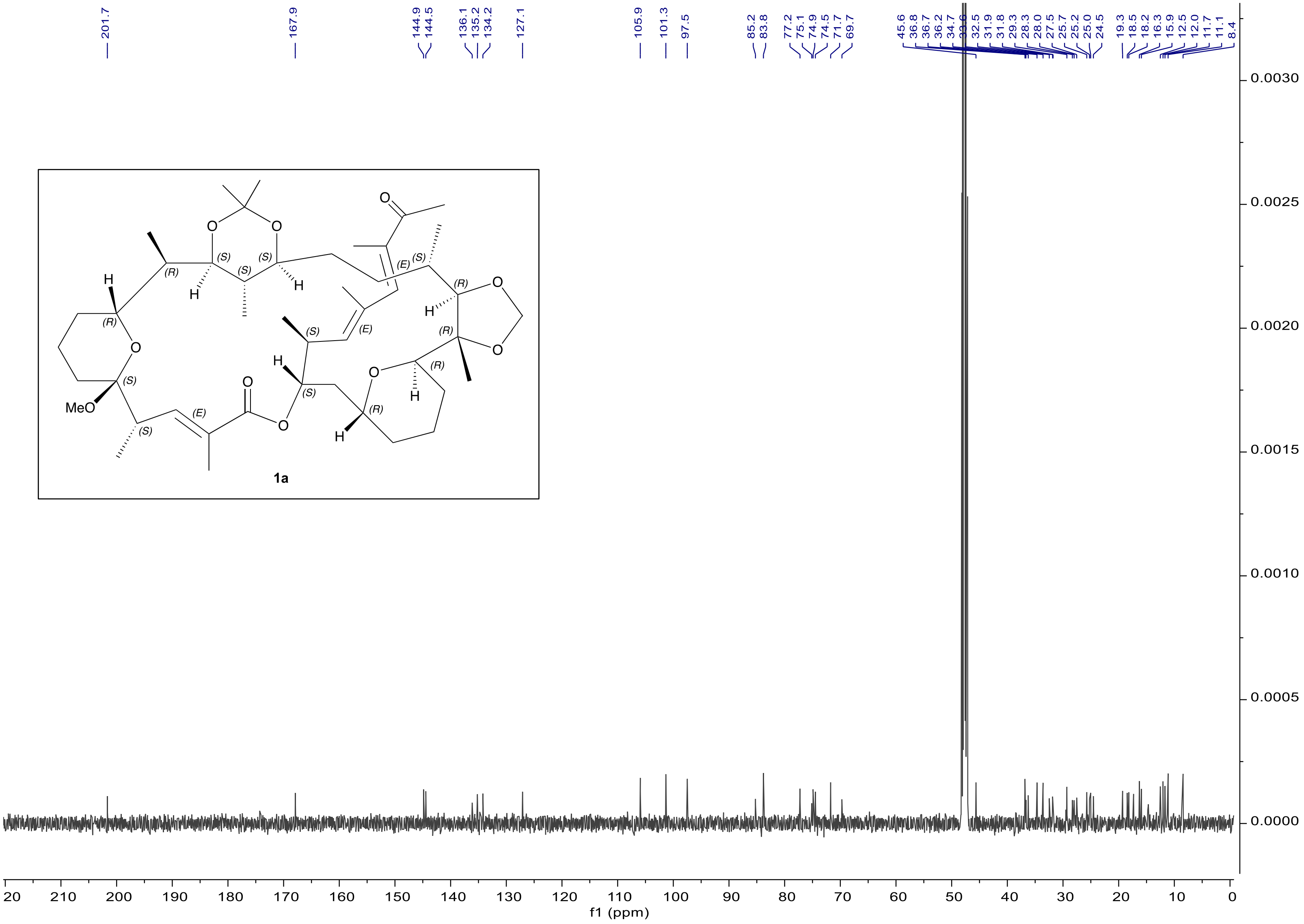

Figure S28. ^{13}C NMR spectrum of *bis*-acetonide (**1a**) in benzene-d_6.

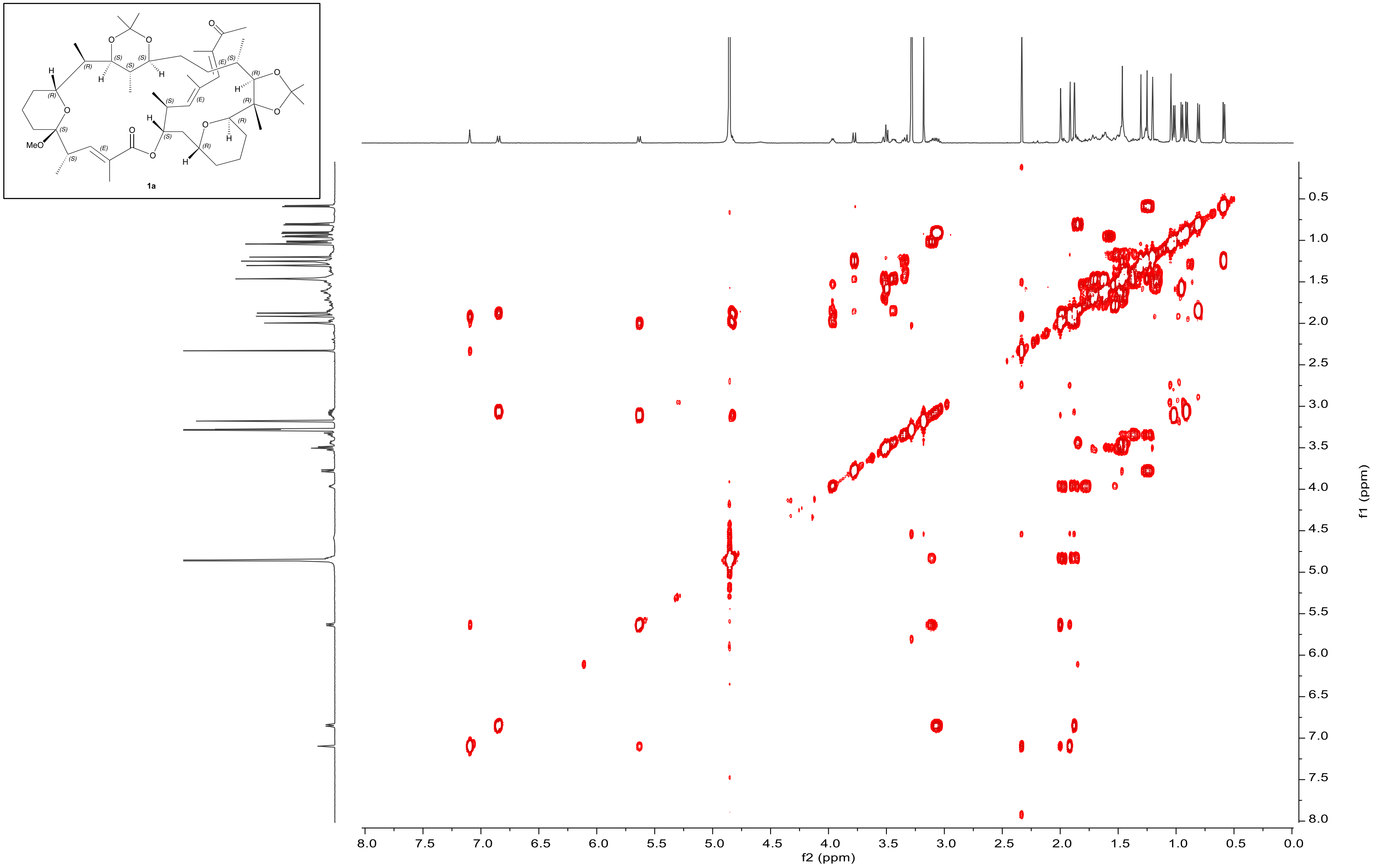

Figure S29. COSY NMR spectrum of *bis*-acetonide (**1a**) in benzene-d_6.

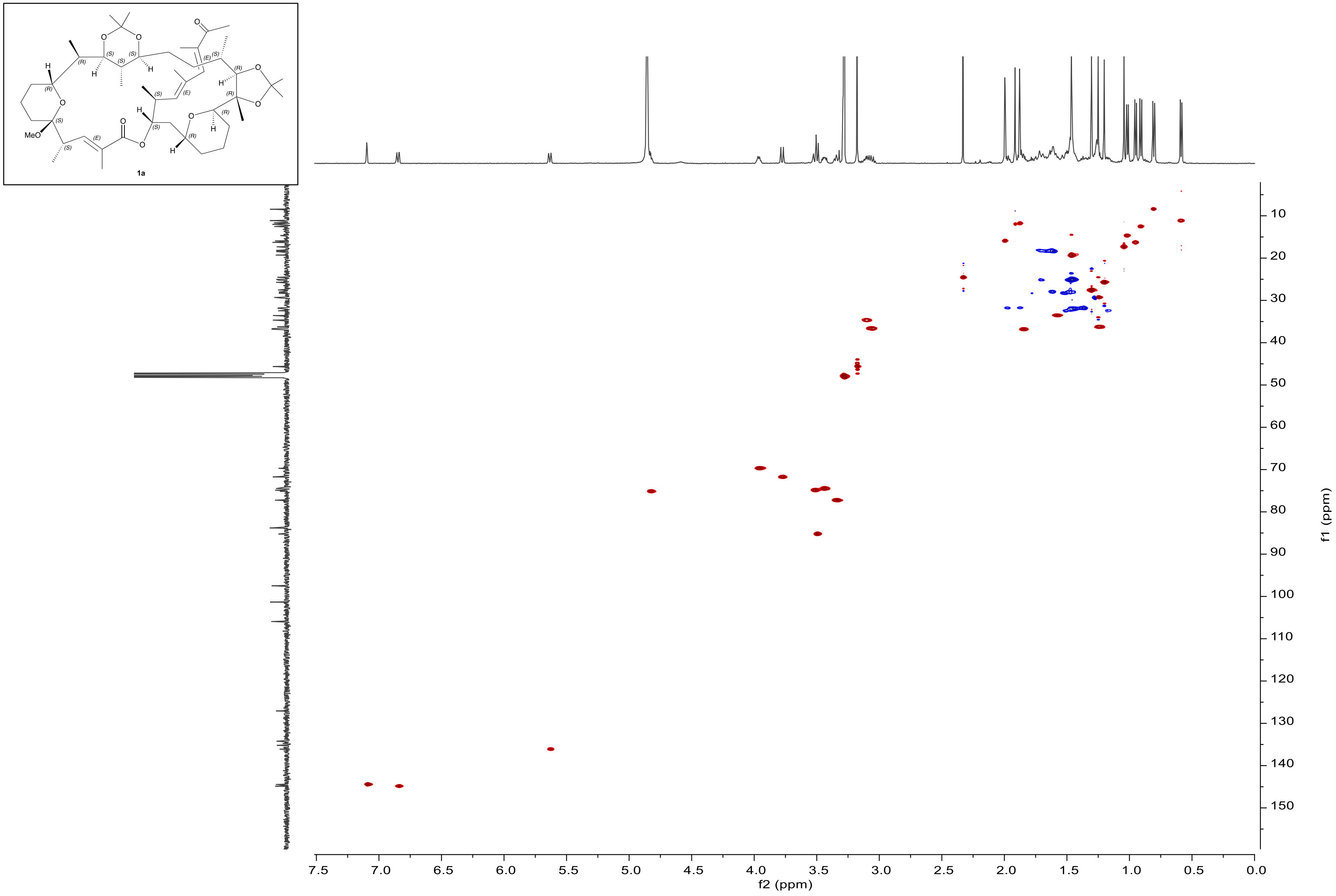

Figure S30. HSQC NMR spectrum of bis-acetonide (**1a**) in benzene-d_6.

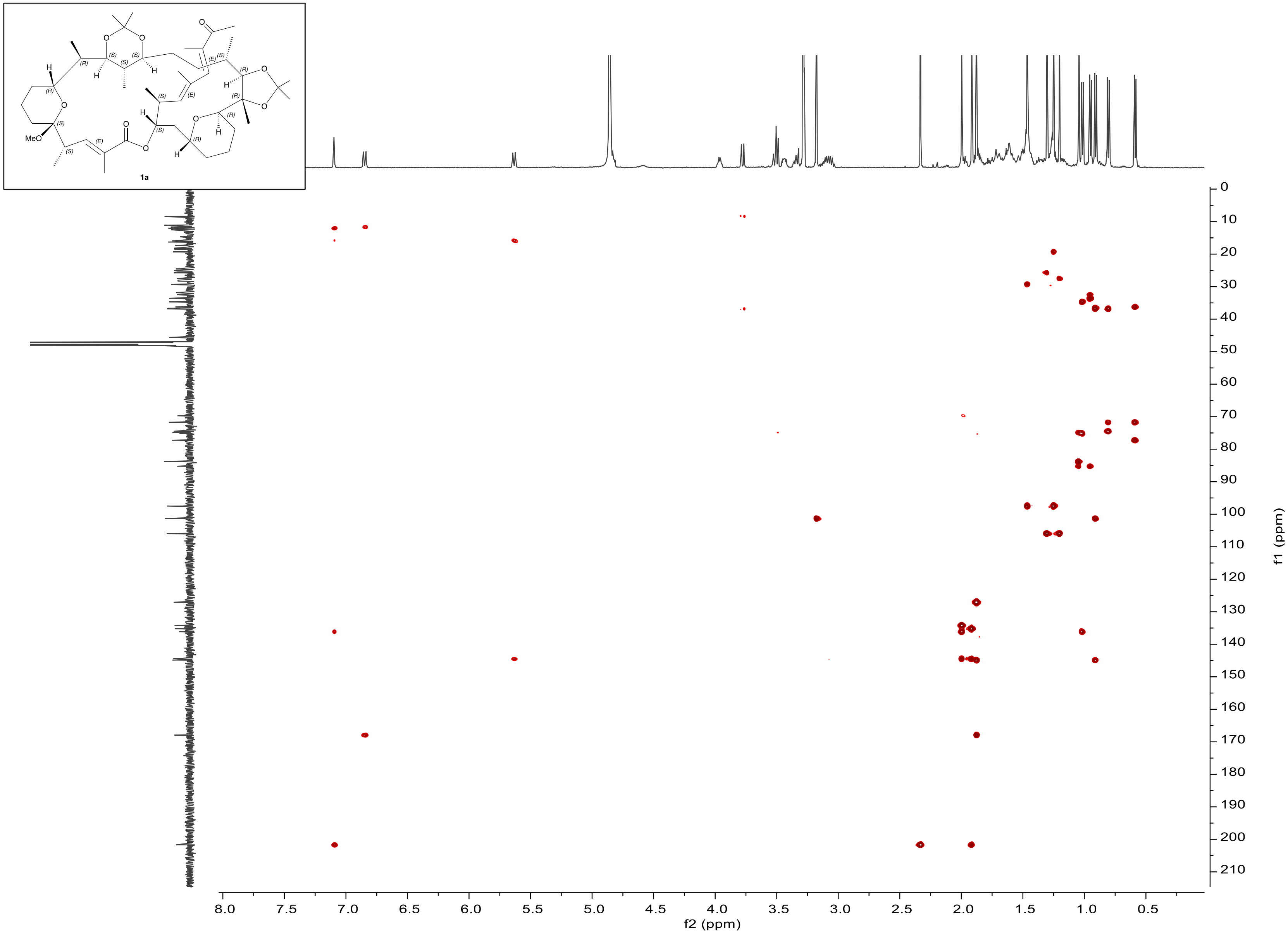

Figure S31. HMBC NMR spectrum of bis-acetonide (**1a**) in benzene-d_6

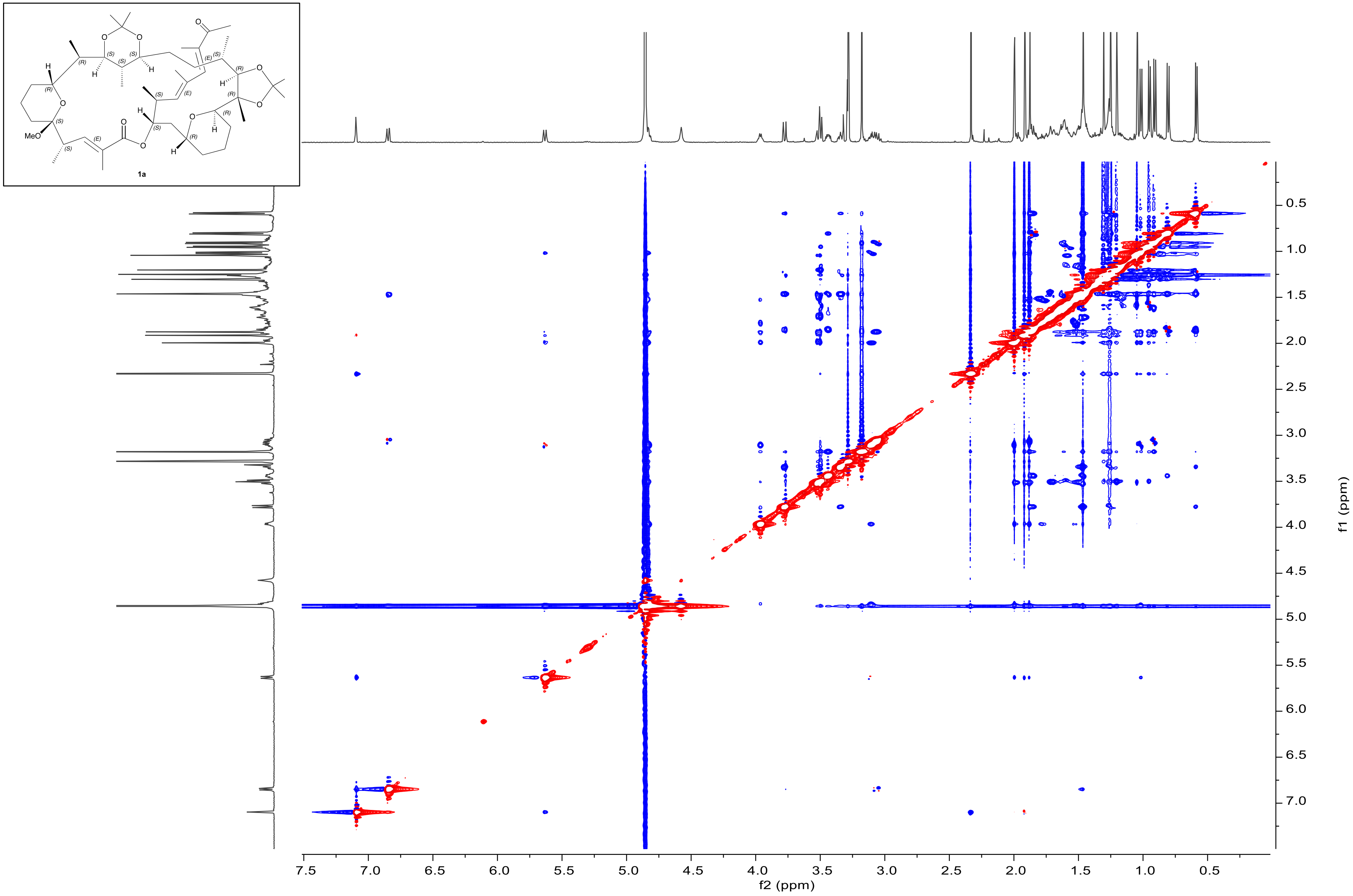

Figure S32. ROESY NMR spectrum of *bis*-acetonide (**1a**) in benzene-d_6.

Table S1. NMR spectroscopic data for marinolides A (**1**) and B (**2**).

	**1**, benzene-d_6		**1**, CD_3OD		**2**, benzene-d_6	
no.	δ_C, mult[a]	δ_H, mult (J in Hz)[b]	δ_C, mult[a]	δ_H, mult (J in Hz)[b]	δ_C, mult[a]	δ_H, mult (J in Hz)[b]
1	168.7, C		168.0, C		166.2, C	
2	130.6, C		128.8, C		130.8, C	
3	141.9, CH	7.19, m[c]	142.7, CH	6.79, dd (10.5, 1.4)	140.8, CH	6.98, d (10.9)
4	44.4, CH	2.53, m[c]	44.8, CH	2.70, m[c]	44.4, CH	2.51, m[c]
5	99.1, C		98.5, C		99.4, C	
6	33.0, CH_2	1.67, m[c]	33.0, CH_2	1.90, m[c]	32.7, CH_2	1.78, m[c]
		1.01, m[c]		1.37, m[c]		0.93, m[c]
7	19.2, CH_2	1.84, m[c]	18.8, CH_2	1.85, m[c]	19.0, CH_2	1.90, m[c]
		1.45, m[c]		1.67, m[c]		1.42, m[c]
8	28.5, CH_2	1.27, m[c]	28.5, CH_2	1.49, m[c]	28.3, CH_2	1.26, m[c]
		1.06, m[c]		1.09, m[c]		1.01, m[c]
9	77.0, CH	4.10, br d (11.5)	75.8, CH	4.02, m	76.2, CH	4.16, d (11.7)
10	38.1, CH	1.47, m[c]	38.5, CH	1.58, m[c]	37.9, CH	1.35, m[c]
11	83.5, CH	3.79, br d (9.3)	79.6, CH	3.58, br d (9.6)	82.1, CH	3.80, br d (9.2)
12	40.5, CH	1.66, m[c]	41.5, CH	1.62, m[c]	40.7, CH	1.41, m[c]
13	77.8, CH	3.63, m	73.8, CH	3.70, m	79.3, CH	3.38, t (9.0)
14	32.0, CH_2	1.46, m[c]	30.2, CH_2	1.47, m[c]	35.0, CH_2	1.43, m[c]
				1.29, m[c]		1.23, m[c]
15	27.7, CH_2	1.66, m[c]	28.4, CH_2	1.45, m[c]	29.2, CH_2	1.42, m[c]
		1.35, m[c]				
16	36.5, CH	1.71, m[c]	37.4, CH	1.44, m[c]	37.8, CH	1.57, m[c]
17	83.0, CH	2.85, d (10.3)	83.0, CH	2.60, d (10.2)	83.6, CH	2.72, d (9.7)
18	63.8, C		63.1, C		63.5, C	
19	63.9, CH	2.91, dd (9.9, 3.1)	63.0, CH	2.79, dd (8.5, 3.4)	62.5, CH	2.90, br d (9.5)
20	29.9, CH_2	1.92, m[c]	29.0, CH_2	1.74, m[c]	28.4, CH_2	1.88, m[c]
		1.47, m[c]		1.45, m[c]		1.53, m[c]
21	22.0, CH_2	1.63, m[c]	22.7, CH_2	1.73, m[c]	23.9, CH_2	1.86, m[c]
				1.40, m[c]		1.49, m[c]
22	38.7, CH_2	1.64, m[c]	38.6, CH_2	1.45, m[c]	38.3, CH_2	1.75, m[c]
				1.40, m[c]		1.32, m[c]
23	68.2, CH	3.85, m	67.0, CH	3.51, m[c]	69.6, CH	3.93, br t (9.5)

24	38.4, CH_2	2.15, br dd (13.7, 10.0)	36.3, CH_2	1.75, m[c]	38.4, CH_2	1.55, m[c]
		1.94, m[c]		1.60, m[c]		1.43, m[c]
25	76.1, CH	5.58, m	72.1, CH	5.53, ddd (11.1, 3.3, 2.0)	65.9, CH	4.54, br d (11.2)
26	40.8, CH	2.31, m	39.1, CH	2.05, m[c]	41.3, CH	2.42, m
27	77.9, CH	4.32, d (8.6)	78.9, CH	3.78, d (9.3)	81.1, CH	4.93, d (11.0)
28	139.8, C		139.2, C		134.9, C	
29	126.6, CH	5.48, d (9.5)	126.8, CH	5.22, d (10.0)	130.5, CH	5.39, br d (10.0)
30	46.3, CH	3.18, m	46.2, CH	3.48, m[c]	46.5, CH	2.97, m
31	210.5, C		211.4, C		206.9, C	
32	28.7, CH_3	1.86, s	27.0, CH_3	2.13, s	27.3 CH_3	1.84, s
33	13.3, CH_3	1.99, br s	12.1, CH_3	1.84, br d (1.0)	13.4, CH_3	1.95, s
34	14.4, CH_3	0.96, d (7.0)	13.5, CH_3	0.99, m[c]	14.5, CH_3	1.06, d (6.9)
35	5.1, CH_3	0.88, d (7.0)	4.5, CH_3	0.87, d (7.0)	4.8, CH_3	0.82, m[c]
36	12.6, CH_3	0.55, d (6.7)	9.9, CH_3	0.72, m[c]	12.9, CH_3	0.38, d (6.9)
37	16.7, CH_3	1.22, d (6.3)	15.2, CH_3	0.97, m[c]	16.1, CH_3	1.11, d (6.2)
38	10.8, CH_3	1.37, s	9.4, CH_3	1.20, s	10.4, CH_3	1.31, s
39	14.4, CH_3	0.83, d (7.0)	10.7, CH_3	0.74, m[c]	9.6, CH_3	0.83, m[c]
40	11.5, CH_3	1.67, br s	10.3, CH_3	1.68, br d (1.0)	11.6, CH_3	1.53, s
41	16.7, CH_3	1.05, d (6.9)	15.3, CH_3	1.07, d (6.7)	15.8, CH_3	0.96, d (6.6)

[a]125 MHz. [b]500 MHz. Chemical shifts (δ) in ppm. [c]Overlapping signals.

Table S2. 2D COSY and/or TOCSY, HMBC and HSQC-TOCSY NMR data of marinolide A (**1**) (500 MHz, benzene-d_6)

no.	δ_C/δ_H	COSY and/or TOCSY	HMBC	HSQC-TOCSY
1	168.7/-			
2	130.6/-			
3	141.9/7.19	H-4, H-33, H-34	C-1, C-5(w)[a], C-33	C-4, C-34
4	44.4/2.53	H-3, H-34	C-2, C-3, C-5, C-34	C-3, C-34
5	99.1/-			
6	33.0/1.67, 1.01	H-7	C-7(w)[a]	C-7
7	19.2/1.84, 1.45	H-6, H-8	C-9(w)[a]	C-8
8	28.5/1.27, 1.06	H-7, H-9		C-9
9	77.0/4.10	H-7, H-8, H-10	C-7, C-10(w)[a], C-11, C-35	C-7, C-8
10	3.81/1.47	H-9, H-11, H-35		C-9
11	83.5/3.79	H-10, H-12, H-36	C-9, C-12, C-13, C-35	C-12, C-13, C-36
12	40.5/1.66	H-11, H-13, H-36		C-13, C-14
13	77.8/3.63	H-12, H-14, H-36		C-11, C-12, C-14, C-36
14	32.0/1.46	H-13, H-15		C-13, C-15
15	27.7/1.66, 1.35	H-14, H-16		C-16
16	36.5/1.71	H-15, H-17		C-17
17	83.0/2.85	H-16, H-37	C-16, C-18, C-37, C-38	C-16, C-37
18	63.8/-			
19	63.9/2.91	H-20	C-18, C-20	C-20, C-21
20	29.9/1.92, 1.47	H-19, H21		C-19, C-21
21	22.0/1.63	H-20, H-22		C-22, C-23
22	38.7/1.64	H-21, H-23		C-21, C-23
23	68.2/3.98	H-22, H24		C-24, C-25
24	38.4/2.15, 1.94	H-23, H-25		C-23, C-25
25	76.1/5.80	H-23, H-24, H-26		C-23, C-24
26	40.8/2.31	H-25, H-27, H-39	C-27	C-27, C-39
27	77.9/4.32	H-26, H-39	C-25, C-26, C28, C-29, C-40	C-26, C-39
28	139.8/-			
29	126.6/5.48	H-30, H-40, H-41	C-27, C-30, C-40, C-41(w)[a]	C-30, C-41
30	46.3/3.18	H-29, H-41	C-28, C-29, C-31, C-41	C-29, C-41
31	210.5/-			
32	28.7/1.86		C-30, C-31	

33	13.3/1.99	H-3	C-1, C-2, C-3	
34	14.4/0.96	H-3, H-4	C-3, C-4, C-5	C-4
35	5.1/0.88	H-10	C-9, C-10, C-11	C-10
36	12.6/0.55	H-11, H-12, H-13	C-11, C-12, C-13	C-11, C-12
37	16.7/1.22	H-16, H-17	C-15, C-16, C-17	C-16, C-17
38	10.8/1.37		C-17, C-18, C-19	
39	14.4/0.83	H-26, H-27	C-25, C-26, C-27	C-26, C-27
40	11.5/1.67	H-29	C-27, C-28, C-29	
41	16.7/1.05	H-29, H-30	C-29, C-30, C-31	C-29, C-30

[a] 'w' denotes weak signal

Table S3. ROESY NMR data for marinolide A (**1**) (500 MHz, benzene-d_6)

no.	δ_C/δ_H	ROESY	no.	δ_C/δ_H	ROESY
1	168.7/-		31	210.5/-	
2	130.6/-		32	28.7/1.86	H-29, H-30
3	141.9/7.19	H-9, H-34, H-39(w)	33	13.3/1.99	H-4, H-35(w)
4	44.4/2.53	H-33	34	14.4/0.96	H-3, H-4, H-6
5	99.1/-		35	5.1/0.88	H-33(w), H-36
6	33.0/1.67, 1.01	H-34	36	12.6/0.55	H-11, H-13, H-35
7	19.2/1.84, 1.45	H-9, H-41	37	16.7/1.22	H-17
8	28.5/1.27, 1.06	H-9	38	10.8/1.37	H-16, H-17
9	77.0/4.10	H-3, H-7, H-10, H-11, H-41	39	14.4/0.83	H-3(w), H-25, H-27
10	3.81/1.47	H-9, H-11, H-12, H-13	40	11.5/1.67	H-23, H-25(w), H-26, H-29, H-30
11	83.5/3.79	H-9, H-10, H-14, H-36	41	16.7/1.05	H-7, H-9
12	40.5/1.66	H-10, H-13			
13	77.8/3.63	H-10, H-12, H-36			
14	32.0/1.46	H-11			
15	27.7/1.66, 1.35				
16	36.5/1.71	H-38			
17	83.0/2.85	H-19, H-37, H-38			
18	63.8/-				
19	63.9/2.91	H-17			
20	29.9/1.92, 1.47				
21	22.0/1.63				
22	38.7/1.64				
23	68.2/3.98	H-25, H-26, H-40			
24	38.4/2.15, 1.94				
25	76.1/5.80	H-23, H-39, H-40(w)			
26	40.8/2.31	H-23, H-40			
27	77.9/4.32	H-39			
28	139.8/-				
29	126.6/5.48	H-30, H-32, H-41			
30	46.3/3.18	H-29, H-32, H-40			

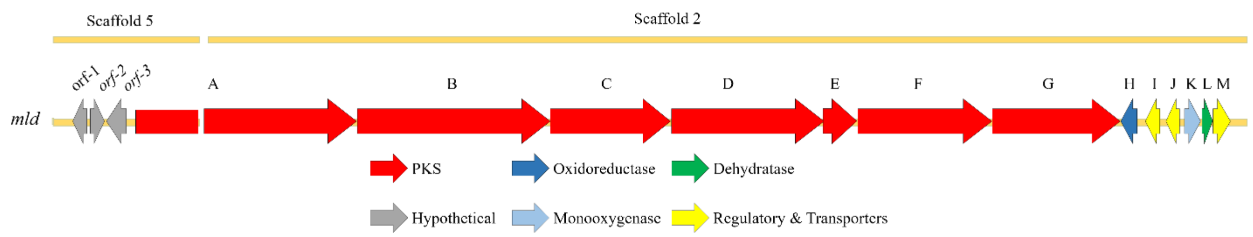

Figure S33. Organization of the marinolide *mld* biosynthetic cluster in actinomycete AJS-327. The polyketide synthase MldA is split across scaffolds 2 and 5. The gap could not be closed with PacBio sequencing, Illumina sequencing, or by PCR.

Table S4. Annotation of the open reading frames in the *mld* biosynthetic cluster, GenBank accession number OL257848

Gene Product	Amino Acid (number)	Proposed function	Sequence similarity (origin)	Similarity/ identity (%)	Accession number
Orf(-1)	394	Hypothetical	*Streptomyces* sp. HNM0575	86/76	WP_169134176
Orf(-2)	384	Cystathionine gamma synthase	*Streptomyces boluensis*	92/87	WP_161700726
Orf(-3)	557	Protein kinase	*Nonomuraea cypriaca*	81/67	WP_195895185
MldA		Polyketide synthase	N/A	N/A	N/A
MldB	5413	Polyketide synthase	N/A	N/A	N/A
MldC	3403	Polyketide synthase	N/A	N/A	N/A
MldD	4285	Polyketide synthase	N/A	N/A	N/A
MldE	952	Polyketide synthase	N/A	N/A	N/A
MldF	3789	Polyketide synthase	N/A	N/A	N/A
MldG	3600	Polyketide synthase	N/A	N/A	N/A
MldH	452	FAD-Dependent oxidoreductase	*Streptomyces camponoticapitis*	73/63	WP_189106772
MldI	409	TerC metal homeostasis membrane protein	*Mycolicibacterium* sp. S18	86/73	WP_197375429
MldJ	380	Helix-turn-helix domain protein	*Streptomyces* sp. CRXT-Y-14	80/77	WP_188340716
MldK	448	Flavin monooxygenase	*Streptomyces* sp. CRXT-Y-14	88/84	WP_188340717
MldL	281	Short chain dehydrogenase	*Streptomyces palmae*	90/85	WP_135342123
MldM	483	MFS transporter	*Streptomyces* sp. CRXT-Y-14	88/83	WP_188341737

Malonyl-CoA	75	106	203
Mld Loading	DDTAYAQ	GHSLGE	HAFH
Mld Mod4	GETVFAQ	GHSVGE	HAFH
Mld Mod5	DQTHYTQ	GHSVGE	HAFH
Mld Mod6	GETVFAQ	GHSVGE	HAFH
Mld Mod9	GETVFAQ	GHSVGE	HAFH
Mld Mod12	GETVFAQ	GHSVGE	HAFH
Mld Mod13	GETVFAQ	GHSVGE	HAFH

Methylmalonyl-CoA			
Mld Mod1*	-------	GAPTLE	YASH
Mld Mod2	GRVDVVQ	GHSQGE	YASH
Mld Mod3	GRVDVVQ	GHSQGE	YASH
Mld Mod7	DRVDVVQ	GHSQGE	YASH
Mld Mod8	GRVDVVQ	GHSQGE	YASH
Mld Mod10	GRVDVVQ	GHSQGE	YASH
Mld Mod11*	-----VQ	GHSQGE	YASH
Mld Mod14	GRVDVVQ	GHSQGE	YASH
Mld Mod15	DRVDVVQ	GHSQGE	YASH

Figure S34. Sequence alignment of key residues for malonyl-CoA and methylmalonyl-CoA selecting acyltransferase (AT) domains in the *mld* polyketide synthases. The marinolide loading domain, module 4, module 5, module 6, module 9, module 12 and module 13 contain the "HAFH" motif indicating the preference for malonyl-CoA, whereas module 1, module 2, module 3, module 7, module 8, module 10, module 11, module 14 and module 15 contain the "YASH" motif indicating a preference for methyl-malonyl-CoA.[1] The asterisk on modules 1 and 11 indicate partial AT domain sequences as the genome around these respective areas could not be closed using PacBio, Illumina, or Sanger sequencing of PCR products.

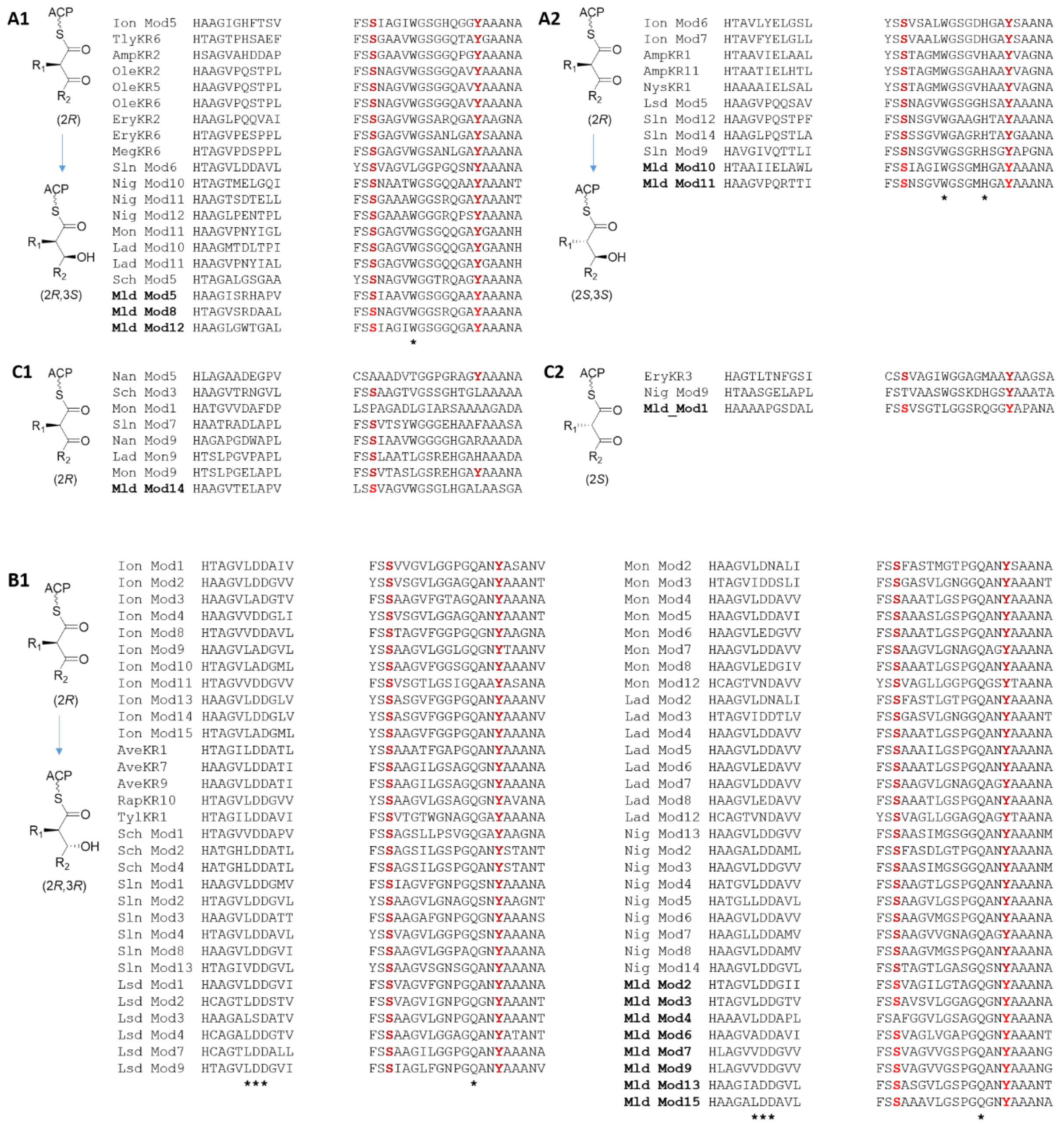

Figure S35. Sequence alignment of key residues for A1, A2, B1, B2, and redox-inactive C1 and C2 ketoreductase domains.[2] Abbreviations: ACP = acyl carrier protein domain; Amp = amphotericin; Ave = avermectin; Ery = erythromycin; Ion = ionostatin; Lsd = lasaloacid; Lad = laidlomycin; Meg = megalomycin; Mld = marinolide; Mon = monensin; Nan = nanchangmycin; Nig = nigericin; Nys = nystatin; Ole = oleandomycin; Pik = pikromycin; Rap = rapamycin; Sln = salinomycin; Sch = calcimycin; and Tyl = tylosin. Active site tyrosine and serine residues are highlighted in red. Signature residues for the A and B classes are marked with an asterisk.

```
Mld Mod2     WLTEHQVNGRTILPGTAV   AANGYHHGPRFR   VLLDAALQ   RLPFVWTDV
Mld Mod3     WLREHTRDGTVVVPSSAV   IECGAAGENVHD   DLMHALLR   RMATQWREV
Mld Mod6     WLVDHGVGGVVVVPGTAF   AGGGFDYGPVFR   ALLDAVLH   GMPFAFSGV
Mld Mod7     WLADHRILDAVLVPGTAL   TGIGFHYGDGFR   ALLDSALH   RLPFSWGEV
Mld Mod9     WLVDHGVGGVVVVPGTAF   AGGGFDYGPVFR   ALLDAVLH   GMPFAFSGV
Mld Mod13    WLVDHGVGGVVVVPGTAF   AGGGFDYGPVFR   ALLDAVLH   GMPFAFSGV
Mld Mod15    WLTDHQVSGVTLLPGTAF   AALGYGYGPAFR   ALLDAGVQ   RLPFAWTGL
                 #   *    *          ** *** *       #   *    *** *
                 HxxxGxxxxP          GYxYGPxF       DxxxQ/H  LPFxW
                    motif              motif         motif    motif
```

Figure S36. Sequence alignment of key residues for dehydratase domains in the marinolide (*mld*) polyketide synthases. DH motifs are shown[3] and based on conserved residues, modules 2, 6, 7, 9, 13, and 15 are predicted to be active. Module 3 is missing the catalytic aspartic acid residue and is predicted to be inactive. Catalytic histidine and aspartic acid residues are marked with #, whereas conserved residues are indicated by an asterisk.

```
Mld Mod6:     FRDVMVALGMYPGR
Mld Mod9:     FRDVLIALGMYPDD
Mld Mod13:    FRDVLIALGMYPGA
```

Figure S37. Sequence alignment of key residues for enoyl reductase domains in the marinolide (*mld*) polyketide synthases. Modules 6, 9 and 13 in the *mld* cluster contain ER domains. For all three modules, Y44 (highlighted in red) is present indicating a 2*S*-2-methyl-acyl product.[4] However, the AT prediction for each of the three modules indicates a preference for malonyl-CoA so there should be no ER-catalyzed epimerization activity on a 2-methyl-2,3-enoylacyl intermediate.

```
                                  160                    185               245         270       380       450
Mld Mod1  (A1): PTTLHGSQTGVFLGATSQDYG  APVGYEGYVLTGSTASVASGR  VTVMATPGMFVEFSKQ  ADGTGWSEGA  KSNIGHTQ  FGISGTNA
Mld Mod2  (D1): PEALRGSQTGTYIGLTYQDYA  ISQELEGYLITGGNASVASGR  ATVMSSPTTFLDFCRQ  ADGFGPGEGV  KSNIGHTQ  FGVSGTNA
Mld Mod3  (L6): AGMLRGSRTGVFAGLMYHEYG  APGEVEGYLANGSAGSVASGR  STVMTSPMLFTELSRQ  ADGFGPGEGV  KSNIGHTQ  FGVSGTNA
Mld Mod4  (I5): PTGLTGRRIGVFVGLNGQDYA  VPEEVEGYLGIGTAASVVSGR  ATVMSAPDTFVEFSRQ  ADGTSWGEGV  KSNIGHTQ  FGVSGTNA
Mld Mod5  (F5): PSTLRGSRTGVFAGTNGQDYA  VPDG-EDFAATGNAASVLSGR  VTVMSTPAAFVGFSRQ  ADGTAWGEGV  KSNIGHTQ  FGVSGTNA
Mld Mod6  (E2): PESLRTSPTGVFVGTSVHDYV  AGGAAEGHLATGNAASVVSGR  ATVMSTPGAFIEFSRQ  ADGTGWGEGV  KSNIGHSQ  FGVSGTNA
Mld Mod7  (N2): PTSLRGGKVGVFSGLIGQGYA  IPPDVEGYIGTGNTASVASGR  VTVMSTPDTFVEFSRQ  ADGTGWAEGA  KSNIGHSQ  FGVSGTNA
Mld Mod8  (L2): VLGLRGSRTGVFVGVIPQDYA  VPEEAAGYVGIGNTTSVVSGR  VTVMSSAATFVEFGRQ  ADGTGWGEGV  KSNIGHTQ  FGVSGTNA
Mld Mod9  (G4): ANTLRNTNTGVFVGASAQSYG  AGEGGEGYFLTGNAASVVSGR  VAVMSNPGAFVEFSRQ  ADGTGWGEGV  KSNIGHTQ  FGVSGTNA
Mld Mod10 (N5): VLGLRGSRTGVFVGVIPQDYA  VPEEAAGYVGIGNTTSVVSGR  VTVMSSAATFVEFGRQ  ADGTGWGEGV  KSNIGHTQ  FGVSGTNA
Mld Mod11 (H3): PELLRGSATGVFVGSNYQDYG  APEGAEGHMLTGGASSVLSGR  VAVMGSPGALVAFSKQ  ADGMGMAEGV  KSNIGHSQ  FGVSGTNA
Mld Mod12 (H7): LTALHGSSTGVFVGAGFQGY-  TADTSDGYFLTGSTGSVVSGR  VTVMSTPGAFVEFSRQ  ADGTGWGEGV  KSNIGHTQ  FGVSGTNA
Mld Mod13 (E6): PESLRTSPTGVFVGTNGQDYV  AGIAAEGYLATGNAASVVSGR  VTVMSTPGAFVEFSRQ  ADGTGWGEGV  KSNIGHTQ  FGVSGTNA
Mld Mod14 (N2): PLSVAGRPVGTFVGTSTSGYG  APPGVEGYLLTGSAAAVASGR  ATVMSSPGTFVEFSRQ  ADGTGWGEGV  KSNIGHTQ  FGVSGTNA
Mld Mod15 (C1): PQEVRGSDTGVFVGTNGQDYA  AGDGMEGYLGTGNAASVVSGR  VTVMSTPGSLLTFARQ  ADGMGMAEGV  KSNIGHTQ  FGMSGTNA
                              ******         ***********       ***   ******        ****            *    *
```

Figure S38. Sequence alignment of select regions for ketosynthase domains in the marinolide (*mld*) polyketide synthases. The selected regions correspond to substrate tunnels identified by the Keatinge-Clay lab, and the 32 asterisks indicate residues that are predicted to be involved in selecting the polyketide substrate, in particular the chemistry at the alpha, beta and gamma positions.[5] Based on the type of substrate they accept, the KS domains from the *mld* PKSs were separated into groups where the KS in module1 is an acetyl acceptor and belongs to group A1, the KS in module 2 belongs to group D1 and is an L-alpha-methyl-beta-ketoacyl acceptor, the KS domains in modules 3 and 8 are trans-alpha/beta-enoyl acceptors and belong to groups L6 and L2, respectively, the KS in module 4 belongs to the I5 group and is a D-alpha-methyl-D-beta-hydroxy acceptor, the KS domain in module 5 is an alpha-unsubstituted-D-beta-hydroxy acceptor and belongs to group F5, the KS domains in modules 6 and 13 belong to groups E2 and E6, respectively, and are alpha-unsubstituted-L-beta-hydroxy acceptors, the KS domains in modules 7 and 14 belong to group N2 and are alpha-unsubstituted-beta-methylene acceptors, the KS in module 9 belongs to group G4 and is a D-alpha-methyl-L-beta-hydroxy acceptor, the KS in module 10 is an alpha-unsubstituted-beta-methylene-gamma-methyl acceptor and belongs to group N5, the KS domains in modules 11 and 12 are L-alpha-methyl-L-beta-hydroxy acceptors and belong to groups H3 and H7, respectively, and the KS in module 15 belongs to group C1 and is a D-alpha-methyl-beta-ketoacyl acceptor.

Table S5. Crystal data and structure refinement for **1a**.

Identification code	fenical001_0m_a_sq	
Empirical formula	C48 H78 O10	
Formula weight	815.10	
Temperature	100.0 K	
Wavelength	1.54178 Å	
Crystal system	Monoclinic	
Space group	P 1 21 1	
Unit cell dimensions	a = 13.3950(3) Å	α= 90°.
	b = 11.0249(3) Å	β= 100.339(2)°.
	c = 33.7472(10) Å	γ = 90°.
Volume	4902.8(2) $Å^3$	
Z	4	
Density (calculated)	1.104 Mg/m^3	
Absorption coefficient	0.604 mm^{-1}	
F(000)	1784	
Crystal size	0.18 x 0.14 x 0.12 mm^3	
Theta range for data collection	1.331 to 70.094°.	
Index ranges	-16<=h<=16, -13<=k<=13, -41<=l<=41	
Reflections collected	92643	
Independent reflections	18579 [R(int) = 0.0489]	
Completeness to theta = 67.679°	100.0 %	
Absorption correction	Semi-empirical from equivalents	
Max. and min. transmission	0.6617 and 0.5766	
Refinement method	Full-matrix least-squares on F^2	
Data / restraints / parameters	18579 / 82 / 1180	
Goodness-of-fit on F^2	1.015	
Final R indices [I>2sigma(I)]	R1 = 0.0378, wR2 = 0.0861	
R indices (all data)	R1 = 0.0456, wR2 = 0.0899	
Absolute structure parameter	0.06(5)	
Extinction coefficient	n/a	
Largest diff. peak and hole	0.291 and -0.201 e.$Å^{-3}$	

Table S6. Atomic coordinates (x 10^4) and equivalent isotropic displacement parameters ($Å^2$x 10^3) for **1a**. U(eq) is defined as one third of the trace of the orthogonalized U^{ij} tensor.

	x	y	z	U(eq)
C(1A)	4866(4)	1671(4)	4141(2)	89(2)
C(2A)	5426(2)	959(3)	3866(1)	44(1)
C(3A)	5998(2)	1609(3)	3594(1)	42(1)
C(4A)	6545(4)	777(4)	3350(2)	86(2)
C(5A)	5998(2)	2831(3)	3586(1)	40(1)
C(6A)	6543(2)	3696(3)	3376(1)	38(1)
C(7A)	7438(3)	3295(3)	3193(1)	50(1)
C(8A)	6254(2)	4869(3)	3370(1)	36(1)
C(9A)	6748(2)	5920(3)	3198(1)	32(1)
C(10A)	5992(2)	6893(3)	3018(1)	41(1)
C(11A)	7590(2)	6416(2)	3532(1)	28(1)
C(12A)	7208(2)	7127(3)	3858(1)	33(1)
C(13A)	8048(2)	7389(3)	4221(1)	36(1)
C(14A)	7895(2)	8558(3)	4440(1)	40(1)
C(15A)	7046(2)	8426(3)	4684(1)	38(1)
C(16A)	7282(2)	7355(3)	4970(1)	36(1)
C(17A)	7436(2)	6204(3)	4736(1)	32(1)
C(35A)	12822(2)	5628(2)	3814(1)	29(1)
C(36A)	13767(2)	5079(3)	4073(1)	39(1)
C(37A)	13023(2)	5993(2)	3396(1)	29(1)
C(38A)	13046(2)	4965(3)	3097(1)	36(1)
C(39A)	13134(2)	5481(3)	2685(1)	41(1)
C(40A)	12266(2)	6370(3)	2546(1)	39(1)
C(41A)	12220(2)	7342(3)	2859(1)	32(1)
C(42A)	13326(3)	8959(3)	3158(1)	66(1)
C(43A)	11037(2)	8652(4)	2354(1)	55(1)
C(44A)	11252(2)	8130(3)	2782(1)	36(1)
C(45A)	10369(2)	7418(2)	2875(1)	31(1)

C(46A)	9695(2)	7751(2)	3097(1)	27(1)
C(47A)	9656(2)	8964(3)	3295(1)	38(1)
C(48A)	8964(2)	6803(2)	3178(1)	28(1)
O(1A)	5445(2)	-151(2)	3883(1)	54(1)
O(2A)	8208(1)	6376(2)	4497(1)	36(1)
O(7A)	12224(1)	6833(2)	3246(1)	27(1)
O(8A)	13110(2)	8046(2)	2857(1)	46(1)
O(9A)	9016(2)	5736(2)	3101(1)	39(1)
O(10A)	8243(1)	7270(2)	3366(1)	28(1)
C(18A)	7675(6)	5080(10)	5009(4)	30(1)
C(19A)	8713(5)	5234(15)	5281(4)	41(2)
C(20A)	6397(5)	3914(6)	5225(2)	38(2)
C(21A)	5376(4)	4078(7)	4957(2)	58(2)
C(22A)	6307(4)	3432(5)	5634(2)	46(1)
C(23A)	7518(5)	3836(7)	4786(2)	35(2)
C(24A)	8424(9)	3059(9)	4717(5)	33(1)
C(25A)	8119(5)	1725(6)	4623(2)	46(1)
C(26A)	8924(3)	3589(5)	4375(1)	41(1)
C(27A)	9879(3)	4084(4)	4473(1)	31(1)
C(28A)	10454(3)	4144(4)	4124(1)	27(1)
C(29A)	11352(4)	2784(4)	3756(1)	28(1)
C(30A)	11868(4)	1559(4)	3812(1)	36(1)
C(31A)	10619(4)	2843(5)	3356(1)	34(1)
C(32A)	11853(10)	4900(6)	3802(4)	23(1)
C(33A)	11319(7)	5059(5)	4166(3)	29(1)
C(34A)	10918(7)	6360(5)	4180(3)	42(1)
O(3A)	6926(3)	5050(6)	5272(2)	32(1)
O(4A)	7021(2)	3112(3)	5051(1)	35(1)
O(5A)	10839(3)	2939(3)	4085(1)	28(1)
O(6A)	12145(4)	3652(5)	3772(2)	26(1)
C(18B)	7893(17)	5110(20)	4993(9)	30(1)
C(19B)	8953(15)	5200(40)	5240(10)	41(2)
C(20B)	6413(12)	4092(17)	5101(4)	38(2)

C(21B)	5375(11)	4711(16)	5012(5)	58(2)
C(22B)	6446(8)	3073(10)	5395(3)	46(1)
C(23B)	7696(13)	3977(19)	4704(6)	35(2)
C(24B)	8450(20)	2940(20)	4757(11)	33(1)
C(25B)	7864(14)	1729(17)	4723(5)	46(1)
C(26B)	9140(7)	2954(12)	4443(3)	41(1)
C(27B)	10090(7)	3454(10)	4439(3)	31(1)
C(28B)	10607(9)	3673(13)	4083(3)	27(1)
C(29B)	11630(9)	2520(11)	3691(4)	28(1)
C(30B)	12267(9)	1394(10)	3734(3)	36(1)
C(31B)	10909(9)	2571(13)	3283(3)	34(1)
C(32B)	11940(20)	4660(19)	3771(9)	23(1)
C(33B)	11350(17)	4741(15)	4116(7)	29(1)
C(34B)	10829(18)	5958(14)	4145(7)	42(1)
O(3B)	7194(10)	4913(15)	5246(5)	32(1)
O(4B)	6663(4)	3692(6)	4724(2)	35(1)
O(5B)	11114(6)	2555(8)	4020(2)	28(1)
O(6B)	12358(10)	3487(14)	3733(5)	26(1)
C(1)	510(2)	8470(3)	922(1)	55(1)
C(2)	1497(2)	9058(2)	1106(1)	28(1)
C(3)	2320(2)	8333(2)	1353(1)	28(1)
C(4)	3213(3)	9076(3)	1549(1)	70(1)
C(5)	2223(2)	7119(2)	1368(1)	23(1)
C(6)	2915(2)	6160(2)	1553(1)	22(1)
C(7)	3981(2)	6457(2)	1759(1)	27(1)
C(8)	2561(2)	5022(2)	1518(1)	23(1)
C(9)	3132(2)	3871(2)	1664(1)	24(1)
C(10)	2432(2)	2902(2)	1789(1)	34(1)
C(11)	3702(2)	3433(2)	1334(1)	21(1)
C(12)	3041(2)	2831(2)	976(1)	24(1)
C(13)	3609(2)	2652(2)	623(1)	26(1)
C(14)	3308(2)	1501(2)	381(1)	31(1)
C(15)	2256(2)	1628(2)	124(1)	30(1)

C(16)	2217(2)	2759(2)	-141(1)	28(1)
C(17)	2528(2)	3867(2)	124(1)	22(1)
C(18)	2550(2)	5075(2)	-105(1)	23(1)
C(19)	3298(2)	5066(3)	-394(1)	32(1)
C(20)	1057(2)	6229(2)	-200(1)	25(1)
C(21)	386(2)	5811(3)	92(1)	30(1)
C(22)	462(2)	6910(2)	-553(1)	34(1)
C(23)	2646(2)	6196(2)	186(1)	22(1)
C(24)	3641(2)	6916(2)	274(1)	24(1)
C(25)	3447(2)	8230(2)	378(1)	33(1)
C(26)	4380(2)	6307(2)	620(1)	28(1)
C(35)	8739(2)	4424(2)	1214(1)	23(1)
C(36)	9459(2)	5207(3)	1020(1)	33(1)
C(37)	9304(2)	3790(2)	1593(1)	22(1)
C(38)	9635(2)	4603(2)	1959(1)	29(1)
C(39)	10035(2)	3839(3)	2333(1)	31(1)
C(40)	9218(2)	2935(2)	2399(1)	28(1)
C(41)	8888(2)	2161(2)	2026(1)	23(1)
C(42)	9737(2)	787(2)	1634(1)	35(1)
C(43)	7944(2)	1366(2)	2037(1)	22(1)
C(44)	8056(2)	615(2)	2425(1)	29(1)
C(45)	7009(2)	2149(2)	1974(1)	22(1)
C(46)	6112(2)	1885(2)	1751(1)	28(1)
C(47)	5844(3)	711(3)	1528(2)	72(1)
C(48)	5341(2)	2879(2)	1700(1)	24(1)
O(1)	1614(1)	10138(2)	1054(1)	39(1)
O(2)	3516(1)	3703(2)	363(1)	24(1)
O(3)	1548(1)	5218(2)	-344(1)	25(1)
O(4)	1853(1)	6983(2)	-11(1)	26(1)
O(7)	8601(1)	2875(1)	1676(1)	20(1)
O(8)	9755(1)	1435(2)	2001(1)	28(1)
O(9)	5499(1)	3913(2)	1812(1)	32(1)
O(10)	4446(1)	2497(1)	1489(1)	24(1)

C(27)	5457(2)	6808(3)	694(1)	32(1)
C(28)	6191(2)	6260(3)	1051(1)	27(1)
C(29)	7421(3)	7005(3)	1602(1)	34(1)
C(30)	7973(3)	8183(3)	1724(2)	53(1)
C(31)	6968(3)	6474(4)	1951(1)	39(1)
C(32)	7791(4)	5121(5)	1305(1)	21(1)
C(33)	6987(3)	5394(3)	933(1)	23(1)
C(34)	6475(3)	4220(4)	749(1)	32(1)
O(5)	6668(2)	7286(2)	1265(1)	35(1)
O(6)	8166(2)	6245(3)	1488(1)	26(1)
C(27')	5317(9)	6091(13)	538(4)	32(1)
C(28')	6108(11)	5854(14)	917(5)	27(1)
C(29')	7270(12)	7044(13)	1387(5)	34(1)
C(30')	7730(13)	8313(12)	1418(7)	53(1)
C(31')	6885(15)	6710(18)	1771(5)	39(1)
C(32')	7800(20)	5050(20)	1218(8)	21(1)
C(33')	6944(13)	5004(15)	851(5)	23(1)
C(34')	6643(13)	3679(15)	752(5)	32(1)
O(5')	6502(8)	7024(11)	1055(4)	35(1)
O(6')	8090(11)	6265(13)	1339(4)	26(1)

Table S7. Bond lengths [Å] and angles [°] for **1a**.

C(1A)-H(1AA)	0.9800	C(12A)-C(13A)	1.535(4)
C(1A)-H(1AB)	0.9800	C(13A)-H(13A)	1.0000
C(1A)-H(1AC)	0.9800	C(13A)-C(14A)	1.517(4)
C(1A)-C(2A)	1.512(5)	C(13A)-O(2A)	1.444(4)
C(2A)-C(3A)	1.483(4)	C(14A)-H(14A)	0.9900
C(2A)-O(1A)	1.225(4)	C(14A)-H(14B)	0.9900
C(3A)-C(4A)	1.506(5)	C(14A)-C(15A)	1.526(4)
C(3A)-C(5A)	1.347(4)	C(15A)-H(15A)	0.9900
C(4A)-H(4AA)	0.9800	C(15A)-H(15B)	0.9900
C(4A)-H(4AB)	0.9800	C(15A)-C(16A)	1.521(4)
C(4A)-H(4AC)	0.9800	C(16A)-H(16A)	0.9900
C(5A)-H(5A)	0.9500	C(16A)-H(16B)	0.9900
C(5A)-C(6A)	1.459(4)	C(16A)-C(17A)	1.526(4)
C(6A)-C(7A)	1.511(4)	C(17A)-H(17A)	1.0000
C(6A)-C(8A)	1.349(4)	C(17A)-H(17B)	1.0000
C(7A)-H(7AA)	0.9800	C(17A)-O(2A)	1.436(3)
C(7A)-H(7AB)	0.9800	C(17A)-C(18A)	1.543(14)
C(7A)-H(7AC)	0.9800	C(17A)-C(18B)	1.55(3)
C(8A)-H(8A)	0.9500	C(35A)-H(35A)	1.0000
C(8A)-C(9A)	1.502(4)	C(35A)-H(35B)	1.0000
C(9A)-H(9A)	1.0000	C(35A)-C(36A)	1.527(4)
C(9A)-C(10A)	1.524(4)	C(35A)-C(37A)	1.538(3)
C(9A)-C(11A)	1.545(4)	C(35A)-C(32A)	1.520(12)
C(10A)-H(10A)	0.9800	C(35A)-C(32B)	1.58(3)
C(10A)-H(10B)	0.9800	C(36A)-H(36A)	0.9800
C(10A)-H(10C)	0.9800	C(36A)-H(36B)	0.9800
C(11A)-H(11A)	1.0000	C(36A)-H(36C)	0.9800
C(11A)-C(12A)	1.515(4)	C(37A)-H(37A)	1.0000
C(11A)-O(10A)	1.464(3)	C(37A)-C(38A)	1.520(4)
C(12A)-H(12A)	0.9900	C(37A)-O(7A)	1.438(3)
C(12A)-H(12B)	0.9900	C(38A)-H(38A)	0.9900

C(38A)-H(38B)	0.9900	C(19A)-H(19A)	0.9800
C(38A)-C(39A)	1.527(4)	C(19A)-H(19B)	0.9800
C(39A)-H(39A)	0.9900	C(19A)-H(19C)	0.9800
C(39A)-H(39B)	0.9900	C(20A)-C(21A)	1.510(8)
C(39A)-C(40A)	1.529(4)	C(20A)-C(22A)	1.504(6)
C(40A)-H(40A)	0.9900	C(20A)-O(3A)	1.434(8)
C(40A)-H(40B)	0.9900	C(20A)-O(4A)	1.414(7)
C(40A)-C(41A)	1.514(4)	C(21A)-H(21A)	0.9800
C(41A)-C(44A)	1.544(4)	C(21A)-H(21B)	0.9800
C(41A)-O(7A)	1.420(3)	C(21A)-H(21C)	0.9800
C(41A)-O(8A)	1.424(3)	C(22A)-H(22A)	0.9800
C(42A)-H(42A)	0.9800	C(22A)-H(22B)	0.9800
C(42A)-H(42B)	0.9800	C(22A)-H(22C)	0.9800
C(42A)-H(42C)	0.9800	C(23A)-H(23A)	1.0000
C(42A)-O(8A)	1.423(5)	C(23A)-C(24A)	1.537(8)
C(43A)-H(43A)	0.9800	C(23A)-O(4A)	1.446(6)
C(43A)-H(43B)	0.9800	C(24A)-H(24A)	1.0000
C(43A)-H(43C)	0.9800	C(24A)-C(25A)	1.544(10)
C(43A)-C(44A)	1.535(4)	C(24A)-C(26A)	1.549(10)
C(44A)-H(44A)	1.0000	C(25A)-H(25A)	0.9800
C(44A)-C(45A)	1.499(4)	C(25A)-H(25B)	0.9800
C(45A)-H(45A)	0.9500	C(25A)-H(25C)	0.9800
C(45A)-C(46A)	1.324(4)	C(26A)-H(26A)	0.9900
C(46A)-C(47A)	1.501(4)	C(26A)-H(26B)	0.9900
C(46A)-C(48A)	1.490(4)	C(26A)-C(27A)	1.375(6)
C(47A)-H(47A)	0.9800	C(27A)-H(27A)	0.9900
C(47A)-H(47B)	0.9800	C(27A)-H(27B)	0.9900
C(47A)-H(47C)	0.9800	C(27A)-C(28A)	1.521(5)
C(48A)-O(9A)	1.209(3)	C(28A)-H(28A)	1.0000
C(48A)-O(10A)	1.348(3)	C(28A)-C(33A)	1.523(8)
C(18A)-C(19A)	1.531(8)	C(28A)-O(5A)	1.440(5)
C(18A)-C(23A)	1.562(8)	C(29A)-C(30A)	1.514(6)
C(18A)-O(3A)	1.453(7)	C(29A)-C(31A)	1.521(6)

C(29A)-O(5A)	1.417(5)	C(23B)-C(24B)	1.52(2)
C(29A)-O(6A)	1.423(7)	C(23B)-O(4B)	1.432(15)
C(30A)-H(30A)	0.9800	C(24B)-H(24B)	1.0000
C(30A)-H(30B)	0.9800	C(24B)-C(25B)	1.55(2)
C(30A)-H(30C)	0.9800	C(24B)-C(26B)	1.521(19)
C(31A)-H(31A)	0.9800	C(25B)-H(25D)	0.9800
C(31A)-H(31B)	0.9800	C(25B)-H(25E)	0.9800
C(31A)-H(31C)	0.9800	C(25B)-H(25F)	0.9800
C(32A)-H(32A)	1.0000	C(26B)-H(26C)	0.9900
C(32A)-C(33A)	1.541(8)	C(26B)-H(26D)	0.9900
C(32A)-O(6A)	1.438(6)	C(26B)-C(27B)	1.389(12)
C(33A)-H(33A)	1.0000	C(27B)-H(27C)	0.9900
C(33A)-C(34A)	1.535(7)	C(27B)-H(27D)	0.9900
C(34A)-H(34A)	0.9800	C(27B)-C(28B)	1.511(13)
C(34A)-H(34B)	0.9800	C(28B)-H(28B)	1.0000
C(34A)-H(34C)	0.9800	C(28B)-C(33B)	1.533(17)
C(18B)-C(19B)	1.52(2)	C(28B)-O(5B)	1.442(13)
C(18B)-C(23B)	1.575(18)	C(29B)-C(30B)	1.498(14)
C(18B)-O(3B)	1.394(18)	C(29B)-C(31B)	1.533(15)
C(19B)-H(19D)	0.9800	C(29B)-O(5B)	1.411(11)
C(19B)-H(19E)	0.9800	C(29B)-O(6B)	1.434(16)
C(19B)-H(19F)	0.9800	C(30B)-H(30D)	0.9800
C(20B)-C(21B)	1.530(17)	C(30B)-H(30E)	0.9800
C(20B)-C(22B)	1.494(15)	C(30B)-H(30F)	0.9800
C(20B)-O(3B)	1.403(18)	C(31B)-H(31D)	0.9800
C(20B)-O(4B)	1.442(15)	C(31B)-H(31E)	0.9800
C(21B)-H(21D)	0.9800	C(31B)-H(31F)	0.9800
C(21B)-H(21E)	0.9800	C(32B)-H(32B)	1.0000
C(21B)-H(21F)	0.9800	C(32B)-C(33B)	1.520(18)
C(22B)-H(22D)	0.9800	C(32B)-O(6B)	1.426(17)
C(22B)-H(22E)	0.9800	C(33B)-H(33B)	1.0000
C(22B)-H(22F)	0.9800	C(33B)-C(34B)	1.523(17)
C(23B)-H(23B)	1.0000	C(34B)-H(34D)	0.9800

C(34B)-H(34E)	0.9800	C(12)-C(13)	1.537(3)
C(34B)-H(34F)	0.9800	C(13)-H(13)	1.0000
C(1)-H(1A)	0.9800	C(13)-C(14)	1.523(3)
C(1)-H(1B)	0.9800	C(13)-O(2)	1.446(3)
C(1)-H(1C)	0.9800	C(14)-H(14C)	0.9900
C(1)-C(2)	1.503(4)	C(14)-H(14D)	0.9900
C(2)-C(3)	1.490(4)	C(14)-C(15)	1.523(4)
C(2)-O(1)	1.217(3)	C(15)-H(15C)	0.9900
C(3)-C(4)	1.502(4)	C(15)-H(15D)	0.9900
C(3)-C(5)	1.347(4)	C(15)-C(16)	1.531(4)
C(4)-H(4A)	0.9800	C(16)-H(16C)	0.9900
C(4)-H(4B)	0.9800	C(16)-H(16D)	0.9900
C(4)-H(4C)	0.9800	C(16)-C(17)	1.527(3)
C(5)-H(5)	0.9500	C(17)-H(17)	1.0000
C(5)-C(6)	1.470(3)	C(17)-C(18)	1.542(3)
C(6)-C(7)	1.507(3)	C(17)-O(2)	1.433(3)
C(6)-C(8)	1.339(3)	C(18)-C(19)	1.519(3)
C(7)-H(7A)	0.9800	C(18)-C(23)	1.568(3)
C(7)-H(7B)	0.9800	C(18)-O(3)	1.447(3)
C(7)-H(7C)	0.9800	C(19)-H(19G)	0.9800
C(8)-H(8)	0.9500	C(19)-H(19H)	0.9800
C(8)-C(9)	1.518(3)	C(19)-H(19I)	0.9800
C(9)-H(9)	1.0000	C(20)-C(21)	1.521(3)
C(9)-C(10)	1.529(4)	C(20)-C(22)	1.508(3)
C(9)-C(11)	1.537(3)	C(20)-O(3)	1.423(3)
C(10)-H(10D)	0.9800	C(20)-O(4)	1.412(3)
C(10)-H(10E)	0.9800	C(21)-H(21G)	0.9800
C(10)-H(10F)	0.9800	C(21)-H(21H)	0.9800
C(11)-H(11)	1.0000	C(21)-H(21I)	0.9800
C(11)-C(12)	1.518(3)	C(22)-H(22G)	0.9800
C(11)-O(10)	1.464(3)	C(22)-H(22H)	0.9800
C(12)-H(12C)	0.9900	C(22)-H(22I)	0.9800
C(12)-H(12D)	0.9900	C(23)-H(23)	1.0000

C(23)-C(24)	1.534(3)	C(40)-H(40D)	0.9900
C(23)-O(4)	1.439(3)	C(40)-C(41)	1.522(3)
C(24)-H(24)	1.0000	C(41)-C(43)	1.545(3)
C(24)-C(25)	1.523(3)	C(41)-O(7)	1.414(3)
C(24)-C(26)	1.542(3)	C(41)-O(8)	1.425(3)
C(25)-H(25G)	0.9800	C(42)-H(42D)	0.9800
C(25)-H(25H)	0.9800	C(42)-H(42E)	0.9800
C(25)-H(25I)	0.9800	C(42)-H(42F)	0.9800
C(26)-H(26E)	0.9900	C(42)-O(8)	1.426(3)
C(26)-H(26F)	0.9900	C(43)-H(43)	1.0000
C(26)-H(26G)	0.9900	C(43)-C(44)	1.532(3)
C(26)-H(26H)	0.9900	C(43)-C(45)	1.506(3)
C(26)-C(27)	1.522(4)	C(44)-H(44B)	0.9800
C(26)-C(27')	1.354(14)	C(44)-H(44C)	0.9800
C(35)-H(35)	1.0000	C(44)-H(44D)	0.9800
C(35)-H(35C)	1.0000	C(45)-H(45)	0.9500
C(35)-C(36)	1.526(3)	C(45)-C(46)	1.330(3)
C(35)-C(37)	1.535(3)	C(46)-C(47)	1.509(4)
C(35)-C(32)	1.562(6)	C(46)-C(48)	1.494(3)
C(35)-C(32')	1.43(3)	C(47)-H(47D)	0.9800
C(36)-H(36D)	0.9800	C(47)-H(47E)	0.9800
C(36)-H(36E)	0.9800	C(47)-H(47F)	0.9800
C(36)-H(36F)	0.9800	C(48)-O(9)	1.207(3)
C(37)-H(37)	1.0000	C(48)-O(10)	1.348(3)
C(37)-C(38)	1.526(3)	C(27)-H(27E)	0.9900
C(37)-O(7)	1.440(3)	C(27)-H(27F)	0.9900
C(38)-H(38C)	0.9900	C(27)-C(28)	1.534(4)
C(38)-H(38D)	0.9900	C(28)-H(28)	1.0000
C(38)-C(39)	1.531(4)	C(28)-C(33)	1.537(5)
C(39)-H(39C)	0.9900	C(28)-O(5)	1.430(4)
C(39)-H(39D)	0.9900	C(29)-C(30)	1.514(5)
C(39)-C(40)	1.526(4)	C(29)-C(31)	1.532(6)
C(40)-H(40C)	0.9900	C(29)-O(5)	1.413(4)

C(29)-O(6)	1.409(4)	C(32')-O(6')	1.43(2)
C(30)-H(30G)	0.9800	C(33')-H(33')	1.0000
C(30)-H(30H)	0.9800	C(33')-C(34')	1.536(17)
C(30)-H(30I)	0.9800	C(34')-H(34J)	0.9800
C(31)-H(31G)	0.9800	C(34')-H(34K)	0.9800
C(31)-H(31H)	0.9800	C(34')-H(34L)	0.9800
C(31)-H(31I)	0.9800		
C(32)-H(32)	1.0000	H(1AA)-C(1A)-H(1AB)	109.5
C(32)-C(33)	1.531(5)	H(1AA)-C(1A)-H(1AC)	109.5
C(32)-O(6)	1.434(5)	H(1AB)-C(1A)-H(1AC)	109.5
C(33)-H(33)	1.0000	C(2A)-C(1A)-H(1AA)	109.5
C(33)-C(34)	1.542(5)	C(2A)-C(1A)-H(1AB)	109.5
C(34)-H(34G)	0.9800	C(2A)-C(1A)-H(1AC)	109.5
C(34)-H(34H)	0.9800	C(3A)-C(2A)-C(1A)	119.8(3)
C(34)-H(34I)	0.9800	O(1A)-C(2A)-C(1A)	119.9(3)
C(27')-H(27G)	0.9900	O(1A)-C(2A)-C(3A)	120.2(3)
C(27')-H(27H)	0.9900	C(2A)-C(3A)-C(4A)	113.5(3)
C(27')-C(28')	1.530(16)	C(5A)-C(3A)-C(2A)	119.8(3)
C(28')-H(28')	1.0000	C(5A)-C(3A)-C(4A)	126.7(3)
C(28')-C(33')	1.506(18)	C(3A)-C(4A)-H(4AA)	109.5
C(28')-O(5')	1.440(15)	C(3A)-C(4A)-H(4AB)	109.5
C(29')-C(30')	1.524(17)	C(3A)-C(4A)-H(4AC)	109.5
C(29')-C(31')	1.522(19)	H(4AA)-C(4A)-H(4AB)	109.5
C(29')-O(5')	1.378(17)	H(4AA)-C(4A)-H(4AC)	109.5
C(29')-O(6')	1.426(18)	H(4AB)-C(4A)-H(4AC)	109.5
C(30')-H(30J)	0.9800	C(3A)-C(5A)-H(5A)	114.1
C(30')-H(30K)	0.9800	C(3A)-C(5A)-C(6A)	131.7(3)
C(30')-H(30L)	0.9800	C(6A)-C(5A)-H(5A)	114.1
C(31')-H(31J)	0.9800	C(5A)-C(6A)-C(7A)	120.7(3)
C(31')-H(31K)	0.9800	C(8A)-C(6A)-C(5A)	117.9(3)
C(31')-H(31L)	0.9800	C(8A)-C(6A)-C(7A)	121.4(3)
C(32')-H(32')	1.0000	C(6A)-C(7A)-H(7AA)	109.5
C(32')-C(33')	1.53(2)	C(6A)-C(7A)-H(7AB)	109.5

C(6A)-C(7A)-H(7AC)	109.5	C(14A)-C(13A)-H(13A)	106.5
H(7AA)-C(7A)-H(7AB)	109.5	O(2A)-C(13A)-C(12A)	111.8(2)
H(7AA)-C(7A)-H(7AC)	109.5	O(2A)-C(13A)-H(13A)	106.5
H(7AB)-C(7A)-H(7AC)	109.5	O(2A)-C(13A)-C(14A)	111.2(2)
C(6A)-C(8A)-H(8A)	116.5	C(13A)-C(14A)-H(14A)	109.4
C(6A)-C(8A)-C(9A)	126.9(3)	C(13A)-C(14A)-H(14B)	109.4
C(9A)-C(8A)-H(8A)	116.5	C(13A)-C(14A)-C(15A)	111.1(2)
C(8A)-C(9A)-H(9A)	107.7	H(14A)-C(14A)-H(14B)	108.0
C(8A)-C(9A)-C(10A)	112.9(2)	C(15A)-C(14A)-H(14A)	109.4
C(8A)-C(9A)-C(11A)	107.8(2)	C(15A)-C(14A)-H(14B)	109.4
C(10A)-C(9A)-H(9A)	107.7	C(14A)-C(15A)-H(15A)	109.9
C(10A)-C(9A)-C(11A)	112.8(2)	C(14A)-C(15A)-H(15B)	109.9
C(11A)-C(9A)-H(9A)	107.7	H(15A)-C(15A)-H(15B)	108.3
C(9A)-C(10A)-H(10A)	109.5	C(16A)-C(15A)-C(14A)	109.0(2)
C(9A)-C(10A)-H(10B)	109.5	C(16A)-C(15A)-H(15A)	109.9
C(9A)-C(10A)-H(10C)	109.5	C(16A)-C(15A)-H(15B)	109.9
H(10A)-C(10A)-H(10B)	109.5	C(15A)-C(16A)-H(16A)	109.5
H(10A)-C(10A)-H(10C)	109.5	C(15A)-C(16A)-H(16B)	109.5
H(10B)-C(10A)-H(10C)	109.5	C(15A)-C(16A)-C(17A)	110.5(2)
C(9A)-C(11A)-H(11A)	108.9	H(16A)-C(16A)-H(16B)	108.1
C(12A)-C(11A)-C(9A)	114.6(2)	C(17A)-C(16A)-H(16A)	109.5
C(12A)-C(11A)-H(11A)	108.9	C(17A)-C(16A)-H(16B)	109.5
O(10A)-C(11A)-C(9A)	110.86(19)	C(16A)-C(17A)-H(17A)	107.3
O(10A)-C(11A)-H(11A)	108.9	C(16A)-C(17A)-H(17B)	110.0
O(10A)-C(11A)-C(12A)	104.4(2)	C(16A)-C(17A)-C(18A)	113.1(4)
C(11A)-C(12A)-H(12A)	109.1	C(16A)-C(17A)-C(18B)	115.9(9)
C(11A)-C(12A)-H(12B)	109.1	O(2A)-C(17A)-C(16A)	111.0(2)
C(11A)-C(12A)-C(13A)	112.5(2)	O(2A)-C(17A)-H(17A)	107.3
H(12A)-C(12A)-H(12B)	107.8	O(2A)-C(17A)-H(17B)	110.0
C(13A)-C(12A)-H(12A)	109.1	O(2A)-C(17A)-C(18A)	110.4(3)
C(13A)-C(12A)-H(12B)	109.1	O(2A)-C(17A)-C(18B)	99.5(8)
C(12A)-C(13A)-H(13A)	106.5	C(18A)-C(17A)-H(17A)	107.3
C(14A)-C(13A)-C(12A)	113.8(2)	C(18B)-C(17A)-H(17B)	110.0

C(36A)-C(35A)-H(35A)	105.2	C(40A)-C(39A)-H(39A)	109.7
C(36A)-C(35A)-H(35B)	109.0	C(40A)-C(39A)-H(39B)	109.7
C(36A)-C(35A)-C(37A)	111.4(2)	C(39A)-C(40A)-H(40A)	109.4
C(36A)-C(35A)-C(32B)	108.4(11)	C(39A)-C(40A)-H(40B)	109.4
C(37A)-C(35A)-H(35A)	105.2	H(40A)-C(40A)-H(40B)	108.0
C(37A)-C(35A)-H(35B)	109.0	C(41A)-C(40A)-C(39A)	111.3(2)
C(37A)-C(35A)-C(32B)	110.1(11)	C(41A)-C(40A)-H(40A)	109.4
C(32A)-C(35A)-H(35A)	105.2	C(41A)-C(40A)-H(40B)	109.4
C(32A)-C(35A)-C(36A)	115.0(5)	C(40A)-C(41A)-C(44A)	114.8(2)
C(32A)-C(35A)-C(37A)	113.7(5)	O(7A)-C(41A)-C(40A)	111.6(2)
C(32B)-C(35A)-H(35B)	109.0	O(7A)-C(41A)-C(44A)	103.9(2)
C(35A)-C(36A)-H(36A)	109.5	O(7A)-C(41A)-O(8A)	110.9(2)
C(35A)-C(36A)-H(36B)	109.5	O(8A)-C(41A)-C(40A)	104.1(2)
C(35A)-C(36A)-H(36C)	109.5	O(8A)-C(41A)-C(44A)	111.8(2)
H(36A)-C(36A)-H(36B)	109.5	H(42A)-C(42A)-H(42B)	109.5
H(36A)-C(36A)-H(36C)	109.5	H(42A)-C(42A)-H(42C)	109.5
H(36B)-C(36A)-H(36C)	109.5	H(42B)-C(42A)-H(42C)	109.5
C(35A)-C(37A)-H(37A)	108.5	O(8A)-C(42A)-H(42A)	109.5
C(38A)-C(37A)-C(35A)	116.2(2)	O(8A)-C(42A)-H(42B)	109.5
C(38A)-C(37A)-H(37A)	108.5	O(8A)-C(42A)-H(42C)	109.5
O(7A)-C(37A)-C(35A)	104.46(19)	H(43A)-C(43A)-H(43B)	109.5
O(7A)-C(37A)-H(37A)	108.5	H(43A)-C(43A)-H(43C)	109.5
O(7A)-C(37A)-C(38A)	110.4(2)	H(43B)-C(43A)-H(43C)	109.5
C(37A)-C(38A)-H(38A)	109.7	C(44A)-C(43A)-H(43A)	109.5
C(37A)-C(38A)-H(38B)	109.7	C(44A)-C(43A)-H(43B)	109.5
C(37A)-C(38A)-C(39A)	109.9(2)	C(44A)-C(43A)-H(43C)	109.5
H(38A)-C(38A)-H(38B)	108.2	C(41A)-C(44A)-H(44A)	107.5
C(39A)-C(38A)-H(38A)	109.7	C(43A)-C(44A)-C(41A)	112.3(2)
C(39A)-C(38A)-H(38B)	109.7	C(43A)-C(44A)-H(44A)	107.5
C(38A)-C(39A)-H(39A)	109.7	C(45A)-C(44A)-C(41A)	109.9(2)
C(38A)-C(39A)-H(39B)	109.7	C(45A)-C(44A)-C(43A)	111.8(2)
C(38A)-C(39A)-C(40A)	109.8(2)	C(45A)-C(44A)-H(44A)	107.5
H(39A)-C(39A)-H(39B)	108.2	C(44A)-C(45A)-H(45A)	116.0

C(46A)-C(45A)-C(44A)	128.1(3)	O(4A)-C(20A)-C(21A)	111.4(5)
C(46A)-C(45A)-H(45A)	116.0	O(4A)-C(20A)-C(22A)	108.1(4)
C(45A)-C(46A)-C(47A)	125.5(2)	O(4A)-C(20A)-O(3A)	105.9(4)
C(45A)-C(46A)-C(48A)	116.5(2)	C(20A)-C(21A)-H(21A)	109.5
C(48A)-C(46A)-C(47A)	117.8(2)	C(20A)-C(21A)-H(21B)	109.5
C(46A)-C(47A)-H(47A)	109.5	C(20A)-C(21A)-H(21C)	109.5
C(46A)-C(47A)-H(47B)	109.5	H(21A)-C(21A)-H(21B)	109.5
C(46A)-C(47A)-H(47C)	109.5	H(21A)-C(21A)-H(21C)	109.5
H(47A)-C(47A)-H(47B)	109.5	H(21B)-C(21A)-H(21C)	109.5
H(47A)-C(47A)-H(47C)	109.5	C(20A)-C(22A)-H(22A)	109.5
H(47B)-C(47A)-H(47C)	109.5	C(20A)-C(22A)-H(22B)	109.5
O(9A)-C(48A)-C(46A)	125.3(2)	C(20A)-C(22A)-H(22C)	109.5
O(9A)-C(48A)-O(10A)	123.1(2)	H(22A)-C(22A)-H(22B)	109.5
O(10A)-C(48A)-C(46A)	111.6(2)	H(22A)-C(22A)-H(22C)	109.5
C(17A)-O(2A)-C(13A)	115.2(2)	H(22B)-C(22A)-H(22C)	109.5
C(41A)-O(7A)-C(37A)	117.25(19)	C(18A)-C(23A)-H(23A)	109.1
C(42A)-O(8A)-C(41A)	116.4(2)	C(24A)-C(23A)-C(18A)	121.3(7)
C(48A)-O(10A)-C(11A)	117.54(19)	C(24A)-C(23A)-H(23A)	109.1
C(17A)-C(18A)-C(23A)	115.0(8)	O(4A)-C(23A)-C(18A)	102.9(5)
C(19A)-C(18A)-C(17A)	109.8(8)	O(4A)-C(23A)-H(23A)	109.1
C(19A)-C(18A)-C(23A)	114.6(8)	O(4A)-C(23A)-C(24A)	104.5(6)
O(3A)-C(18A)-C(17A)	106.8(7)	C(23A)-C(24A)-H(24A)	107.9
O(3A)-C(18A)-C(19A)	106.8(7)	C(23A)-C(24A)-C(25A)	112.0(7)
O(3A)-C(18A)-C(23A)	103.0(6)	C(23A)-C(24A)-C(26A)	111.2(7)
C(18A)-C(19A)-H(19A)	109.5	C(25A)-C(24A)-H(24A)	107.9
C(18A)-C(19A)-H(19B)	109.5	C(25A)-C(24A)-C(26A)	109.8(8)
C(18A)-C(19A)-H(19C)	109.5	C(26A)-C(24A)-H(24A)	107.9
H(19A)-C(19A)-H(19B)	109.5	C(24A)-C(25A)-H(25A)	109.5
H(19A)-C(19A)-H(19C)	109.5	C(24A)-C(25A)-H(25B)	109.5
H(19B)-C(19A)-H(19C)	109.5	C(24A)-C(25A)-H(25C)	109.5
C(22A)-C(20A)-C(21A)	112.3(5)	H(25A)-C(25A)-H(25B)	109.5
O(3A)-C(20A)-C(21A)	109.8(6)	H(25A)-C(25A)-H(25C)	109.5
O(3A)-C(20A)-C(22A)	109.1(5)	H(25B)-C(25A)-H(25C)	109.5

C(24A)-C(26A)-H(26A)	107.7
C(24A)-C(26A)-H(26B)	107.7
H(26A)-C(26A)-H(26B)	107.1
C(27A)-C(26A)-C(24A)	118.5(7)
C(27A)-C(26A)-H(26A)	107.7
C(27A)-C(26A)-H(26B)	107.7
C(26A)-C(27A)-H(27A)	108.7
C(26A)-C(27A)-H(27B)	108.7
C(26A)-C(27A)-C(28A)	114.1(4)
H(27A)-C(27A)-H(27B)	107.6
C(28A)-C(27A)-H(27A)	108.7
C(28A)-C(27A)-H(27B)	108.7
C(27A)-C(28A)-H(28A)	108.3
C(27A)-C(28A)-C(33A)	116.1(4)
C(33A)-C(28A)-H(28A)	108.3
O(5A)-C(28A)-C(27A)	105.7(3)
O(5A)-C(28A)-H(28A)	108.3
O(5A)-C(28A)-C(33A)	109.9(4)
C(30A)-C(29A)-C(31A)	110.9(4)
O(5A)-C(29A)-C(30A)	106.5(4)
O(5A)-C(29A)-C(31A)	111.3(4)
O(5A)-C(29A)-O(6A)	110.6(4)
O(6A)-C(29A)-C(30A)	106.0(4)
O(6A)-C(29A)-C(31A)	111.4(5)
C(29A)-C(30A)-H(30A)	109.5
C(29A)-C(30A)-H(30B)	109.5
C(29A)-C(30A)-H(30C)	109.5
H(30A)-C(30A)-H(30B)	109.5
H(30A)-C(30A)-H(30C)	109.5
H(30B)-C(30A)-H(30C)	109.5
C(29A)-C(31A)-H(31A)	109.5
C(29A)-C(31A)-H(31B)	109.5
C(29A)-C(31A)-H(31C)	109.5
H(31A)-C(31A)-H(31B)	109.5
H(31A)-C(31A)-H(31C)	109.5
H(31B)-C(31A)-H(31C)	109.5
C(35A)-C(32A)-H(32A)	108.6
C(35A)-C(32A)-C(33A)	115.7(8)
C(33A)-C(32A)-H(32A)	108.6
O(6A)-C(32A)-C(35A)	105.4(7)
O(6A)-C(32A)-H(32A)	108.6
O(6A)-C(32A)-C(33A)	109.6(6)
C(28A)-C(33A)-C(32A)	107.7(6)
C(28A)-C(33A)-H(33A)	109.3
C(28A)-C(33A)-C(34A)	110.9(6)
C(32A)-C(33A)-H(33A)	109.3
C(34A)-C(33A)-C(32A)	110.1(6)
C(34A)-C(33A)-H(33A)	109.3
C(33A)-C(34A)-H(34A)	109.5
C(33A)-C(34A)-H(34B)	109.5
C(33A)-C(34A)-H(34C)	109.5
H(34A)-C(34A)-H(34B)	109.5
H(34A)-C(34A)-H(34C)	109.5
H(34B)-C(34A)-H(34C)	109.5
C(20A)-O(3A)-C(18A)	109.5(5)
C(20A)-O(4A)-C(23A)	106.5(4)
C(29A)-O(5A)-C(28A)	114.6(3)
C(29A)-O(6A)-C(32A)	115.9(6)
C(17A)-C(18B)-C(23B)	105.5(19)
C(19B)-C(18B)-C(17A)	120(2)
C(19B)-C(18B)-C(23B)	115(2)
O(3B)-C(18B)-C(17A)	102.9(17)
O(3B)-C(18B)-C(19B)	110(2)
O(3B)-C(18B)-C(23B)	101.5(14)
C(18B)-C(19B)-H(19D)	109.5
C(18B)-C(19B)-H(19E)	109.5

C(18B)-C(19B)-H(19F)	109.5	C(26B)-C(24B)-C(25B)	108.6(17)
H(19D)-C(19B)-H(19E)	109.5	C(24B)-C(25B)-H(25D)	109.5
H(19D)-C(19B)-H(19F)	109.5	C(24B)-C(25B)-H(25E)	109.5
H(19E)-C(19B)-H(19F)	109.5	C(24B)-C(25B)-H(25F)	109.5
C(22B)-C(20B)-C(21B)	112.5(13)	H(25D)-C(25B)-H(25E)	109.5
O(3B)-C(20B)-C(21B)	112.0(14)	H(25D)-C(25B)-H(25F)	109.5
O(3B)-C(20B)-C(22B)	108.8(14)	H(25E)-C(25B)-H(25F)	109.5
O(3B)-C(20B)-O(4B)	103.2(11)	C(24B)-C(26B)-H(26C)	104.1
O(4B)-C(20B)-C(21B)	108.1(11)	C(24B)-C(26B)-H(26D)	104.1
O(4B)-C(20B)-C(22B)	111.9(13)	H(26C)-C(26B)-H(26D)	105.5
C(20B)-C(21B)-H(21D)	109.5	C(27B)-C(26B)-C(24B)	132.6(15)
C(20B)-C(21B)-H(21E)	109.5	C(27B)-C(26B)-H(26C)	104.1
C(20B)-C(21B)-H(21F)	109.5	C(27B)-C(26B)-H(26D)	104.1
H(21D)-C(21B)-H(21E)	109.5	C(26B)-C(27B)-H(27C)	105.2
H(21D)-C(21B)-H(21F)	109.5	C(26B)-C(27B)-H(27D)	105.2
H(21E)-C(21B)-H(21F)	109.5	C(26B)-C(27B)-C(28B)	128.4(9)
C(20B)-C(22B)-H(22D)	109.5	H(27C)-C(27B)-H(27D)	105.9
C(20B)-C(22B)-H(22E)	109.5	C(28B)-C(27B)-H(27C)	105.2
C(20B)-C(22B)-H(22F)	109.5	C(28B)-C(27B)-H(27D)	105.2
H(22D)-C(22B)-H(22E)	109.5	C(27B)-C(28B)-H(28B)	107.7
H(22D)-C(22B)-H(22F)	109.5	C(27B)-C(28B)-C(33B)	116.7(11)
H(22E)-C(22B)-H(22F)	109.5	C(33B)-C(28B)-H(28B)	107.7
C(18B)-C(23B)-H(23B)	105.7	O(5B)-C(28B)-C(27B)	106.0(9)
C(24B)-C(23B)-C(18B)	119.3(19)	O(5B)-C(28B)-H(28B)	107.7
C(24B)-C(23B)-H(23B)	105.7	O(5B)-C(28B)-C(33B)	110.5(11)
O(4B)-C(23B)-C(18B)	101.7(11)	C(30B)-C(29B)-C(31B)	112.0(9)
O(4B)-C(23B)-H(23B)	105.7	O(5B)-C(29B)-C(30B)	107.2(9)
O(4B)-C(23B)-C(24B)	117.6(15)	O(5B)-C(29B)-C(31B)	112.7(9)
C(23B)-C(24B)-H(24B)	108.9	O(5B)-C(29B)-O(6B)	108.8(10)
C(23B)-C(24B)-C(25B)	108.5(18)	O(6B)-C(29B)-C(30B)	103.9(10)
C(23B)-C(24B)-C(26B)	112.9(19)	O(6B)-C(29B)-C(31B)	111.7(12)
C(25B)-C(24B)-H(24B)	108.9	C(29B)-C(30B)-H(30D)	109.5
C(26B)-C(24B)-H(24B)	108.9	C(29B)-C(30B)-H(30E)	109.5

C(29B)-C(30B)-H(30F)	109.5	H(1A)-C(1)-H(1C)	109.5
H(30D)-C(30B)-H(30E)	109.5	H(1B)-C(1)-H(1C)	109.5
H(30D)-C(30B)-H(30F)	109.5	C(2)-C(1)-H(1A)	109.5
H(30E)-C(30B)-H(30F)	109.5	C(2)-C(1)-H(1B)	109.5
C(29B)-C(31B)-H(31D)	109.5	C(2)-C(1)-H(1C)	109.5
C(29B)-C(31B)-H(31E)	109.5	C(3)-C(2)-C(1)	120.5(2)
C(29B)-C(31B)-H(31F)	109.5	O(1)-C(2)-C(1)	119.3(2)
H(31D)-C(31B)-H(31E)	109.5	O(1)-C(2)-C(3)	120.2(2)
H(31D)-C(31B)-H(31F)	109.5	C(2)-C(3)-C(4)	113.9(2)
H(31E)-C(31B)-H(31F)	109.5	C(5)-C(3)-C(2)	119.2(2)
C(35A)-C(32B)-H(32B)	107.8	C(5)-C(3)-C(4)	126.9(3)
C(33B)-C(32B)-C(35A)	111.9(17)	C(3)-C(4)-H(4A)	109.5
C(33B)-C(32B)-H(32B)	107.8	C(3)-C(4)-H(4B)	109.5
O(6B)-C(32B)-C(35A)	108.6(19)	C(3)-C(4)-H(4C)	109.5
O(6B)-C(32B)-H(32B)	107.8	H(4A)-C(4)-H(4B)	109.5
O(6B)-C(32B)-C(33B)	112.9(17)	H(4A)-C(4)-H(4C)	109.5
C(28B)-C(33B)-H(33B)	107.2	H(4B)-C(4)-H(4C)	109.5
C(32B)-C(33B)-C(28B)	108.5(15)	C(3)-C(5)-H(5)	113.9
C(32B)-C(33B)-H(33B)	107.2	C(3)-C(5)-C(6)	132.3(2)
C(32B)-C(33B)-C(34B)	114.0(17)	C(6)-C(5)-H(5)	113.9
C(34B)-C(33B)-C(28B)	112.5(15)	C(5)-C(6)-C(7)	120.9(2)
C(34B)-C(33B)-H(33B)	107.2	C(8)-C(6)-C(5)	116.7(2)
C(33B)-C(34B)-H(34D)	109.5	C(8)-C(6)-C(7)	122.4(2)
C(33B)-C(34B)-H(34E)	109.5	C(6)-C(7)-H(7A)	109.5
C(33B)-C(34B)-H(34F)	109.5	C(6)-C(7)-H(7B)	109.5
H(34D)-C(34B)-H(34E)	109.5	C(6)-C(7)-H(7C)	109.5
H(34D)-C(34B)-H(34F)	109.5	H(7A)-C(7)-H(7B)	109.5
H(34E)-C(34B)-H(34F)	109.5	H(7A)-C(7)-H(7C)	109.5
C(18B)-O(3B)-C(20B)	115.3(13)	H(7B)-C(7)-H(7C)	109.5
C(23B)-O(4B)-C(20B)	110.6(11)	C(6)-C(8)-H(8)	116.4
C(29B)-O(5B)-C(28B)	116.7(8)	C(6)-C(8)-C(9)	127.3(2)
C(32B)-O(6B)-C(29B)	114.2(15)	C(9)-C(8)-H(8)	116.4
H(1A)-C(1)-H(1B)	109.5	C(8)-C(9)-H(9)	107.8

C(8)-C(9)-C(10)	112.1(2)	C(15)-C(14)-H(14C)	109.4
C(8)-C(9)-C(11)	108.28(19)	C(15)-C(14)-H(14D)	109.4
C(10)-C(9)-H(9)	107.8	C(14)-C(15)-H(15C)	109.7
C(10)-C(9)-C(11)	112.9(2)	C(14)-C(15)-H(15D)	109.7
C(11)-C(9)-H(9)	107.8	C(14)-C(15)-C(16)	109.8(2)
C(9)-C(10)-H(10D)	109.5	H(15C)-C(15)-H(15D)	108.2
C(9)-C(10)-H(10E)	109.5	C(16)-C(15)-H(15C)	109.7
C(9)-C(10)-H(10F)	109.5	C(16)-C(15)-H(15D)	109.7
H(10D)-C(10)-H(10E)	109.5	C(15)-C(16)-H(16C)	109.8
H(10D)-C(10)-H(10F)	109.5	C(15)-C(16)-H(16D)	109.8
H(10E)-C(10)-H(10F)	109.5	H(16C)-C(16)-H(16D)	108.2
C(9)-C(11)-H(11)	109.0	C(17)-C(16)-C(15)	109.4(2)
C(12)-C(11)-C(9)	114.81(19)	C(17)-C(16)-H(16C)	109.8
C(12)-C(11)-H(11)	109.0	C(17)-C(16)-H(16D)	109.8
O(10)-C(11)-C(9)	110.94(18)	C(16)-C(17)-H(17)	107.9
O(10)-C(11)-H(11)	109.0	C(16)-C(17)-C(18)	115.19(19)
O(10)-C(11)-C(12)	103.84(18)	C(18)-C(17)-H(17)	107.9
C(11)-C(12)-H(12C)	109.2	O(2)-C(17)-C(16)	110.89(18)
C(11)-C(12)-H(12D)	109.2	O(2)-C(17)-H(17)	107.9
C(11)-C(12)-C(13)	111.97(19)	O(2)-C(17)-C(18)	106.94(18)
H(12C)-C(12)-H(12D)	107.9	C(17)-C(18)-C(23)	112.03(18)
C(13)-C(12)-H(12C)	109.2	C(19)-C(18)-C(17)	112.9(2)
C(13)-C(12)-H(12D)	109.2	C(19)-C(18)-C(23)	114.6(2)
C(12)-C(13)-H(13)	106.8	O(3)-C(18)-C(17)	105.91(18)
C(14)-C(13)-C(12)	113.7(2)	O(3)-C(18)-C(19)	107.15(19)
C(14)-C(13)-H(13)	106.8	O(3)-C(18)-C(23)	103.17(18)
O(2)-C(13)-C(12)	111.46(18)	C(18)-C(19)-H(19G)	109.5
O(2)-C(13)-H(13)	106.8	C(18)-C(19)-H(19H)	109.5
O(2)-C(13)-C(14)	110.91(19)	C(18)-C(19)-H(19I)	109.5
C(13)-C(14)-H(14C)	109.4	H(19G)-C(19)-H(19H)	109.5
C(13)-C(14)-H(14D)	109.4	H(19G)-C(19)-H(19I)	109.5
H(14C)-C(14)-H(14D)	108.0	H(19H)-C(19)-H(19I)	109.5
C(15)-C(14)-C(13)	111.00(19)	C(22)-C(20)-C(21)	111.8(2)

O(3)-C(20)-C(21)	110.4(2)	H(25G)-C(25)-H(25I)	109.5
O(3)-C(20)-C(22)	109.4(2)	H(25H)-C(25)-H(25I)	109.5
O(4)-C(20)-C(21)	111.84(19)	C(24)-C(26)-H(26E)	108.4
O(4)-C(20)-C(22)	108.2(2)	C(24)-C(26)-H(26F)	108.4
O(4)-C(20)-O(3)	104.93(18)	C(24)-C(26)-H(26G)	108.6
C(20)-C(21)-H(21G)	109.5	C(24)-C(26)-H(26H)	108.6
C(20)-C(21)-H(21H)	109.5	H(26E)-C(26)-H(26F)	107.5
C(20)-C(21)-H(21I)	109.5	H(26G)-C(26)-H(26H)	107.6
H(21G)-C(21)-H(21H)	109.5	C(27)-C(26)-C(24)	115.3(2)
H(21G)-C(21)-H(21I)	109.5	C(27)-C(26)-H(26E)	108.4
H(21H)-C(21)-H(21I)	109.5	C(27)-C(26)-H(26F)	108.4
C(20)-C(22)-H(22G)	109.5	C(27')-C(26)-C(24)	114.5(6)
C(20)-C(22)-H(22H)	109.5	C(27')-C(26)-H(26G)	108.6
C(20)-C(22)-H(22I)	109.5	C(27')-C(26)-H(26H)	108.6
H(22G)-C(22)-H(22H)	109.5	C(36)-C(35)-H(35)	106.8
H(22G)-C(22)-H(22I)	109.5	C(36)-C(35)-H(35C)	104.2
H(22H)-C(22)-H(22I)	109.5	C(36)-C(35)-C(37)	111.2(2)
C(18)-C(23)-H(23)	108.4	C(36)-C(35)-C(32)	113.9(3)
C(24)-C(23)-C(18)	120.04(19)	C(37)-C(35)-H(35)	106.8
C(24)-C(23)-H(23)	108.4	C(37)-C(35)-H(35C)	104.2
O(4)-C(23)-C(18)	102.43(17)	C(37)-C(35)-C(32)	110.8(2)
O(4)-C(23)-H(23)	108.4	C(32)-C(35)-H(35)	106.8
O(4)-C(23)-C(24)	108.73(18)	C(32')-C(35)-H(35C)	104.2
C(23)-C(24)-H(24)	108.4	C(32')-C(35)-C(36)	110.6(12)
C(23)-C(24)-C(26)	109.82(19)	C(32')-C(35)-C(37)	120.7(12)
C(25)-C(24)-C(23)	111.0(2)	C(35)-C(36)-H(36D)	109.5
C(25)-C(24)-H(24)	108.4	C(35)-C(36)-H(36E)	109.5
C(25)-C(24)-C(26)	110.7(2)	C(35)-C(36)-H(36F)	109.5
C(26)-C(24)-H(24)	108.4	H(36D)-C(36)-H(36E)	109.5
C(24)-C(25)-H(25G)	109.5	H(36D)-C(36)-H(36F)	109.5
C(24)-C(25)-H(25H)	109.5	H(36E)-C(36)-H(36F)	109.5
C(24)-C(25)-H(25I)	109.5	C(35)-C(37)-H(37)	108.6
H(25G)-C(25)-H(25H)	109.5	C(38)-C(37)-C(35)	115.9(2)

C(38)-C(37)-H(37)	108.6	O(8)-C(42)-H(42F)	109.5
O(7)-C(37)-C(35)	103.86(18)	C(41)-C(43)-H(43)	107.5
O(7)-C(37)-H(37)	108.6	C(44)-C(43)-C(41)	111.91(19)
O(7)-C(37)-C(38)	111.09(19)	C(44)-C(43)-H(43)	107.5
C(37)-C(38)-H(38C)	109.5	C(45)-C(43)-C(41)	109.60(19)
C(37)-C(38)-H(38D)	109.5	C(45)-C(43)-H(43)	107.5
C(37)-C(38)-C(39)	110.5(2)	C(45)-C(43)-C(44)	112.46(19)
H(38C)-C(38)-H(38D)	108.1	C(43)-C(44)-H(44B)	109.5
C(39)-C(38)-H(38C)	109.5	C(43)-C(44)-H(44C)	109.5
C(39)-C(38)-H(38D)	109.5	C(43)-C(44)-H(44D)	109.5
C(38)-C(39)-H(39C)	109.9	H(44B)-C(44)-H(44C)	109.5
C(38)-C(39)-H(39D)	109.9	H(44B)-C(44)-H(44D)	109.5
H(39C)-C(39)-H(39D)	108.3	H(44C)-C(44)-H(44D)	109.5
C(40)-C(39)-C(38)	109.0(2)	C(43)-C(45)-H(45)	116.6
C(40)-C(39)-H(39C)	109.9	C(46)-C(45)-C(43)	126.8(2)
C(40)-C(39)-H(39D)	109.9	C(46)-C(45)-H(45)	116.6
C(39)-C(40)-H(40C)	109.5	C(45)-C(46)-C(47)	125.5(2)
C(39)-C(40)-H(40D)	109.5	C(45)-C(46)-C(48)	116.2(2)
H(40C)-C(40)-H(40D)	108.1	C(48)-C(46)-C(47)	118.3(2)
C(41)-C(40)-C(39)	110.7(2)	C(46)-C(47)-H(47D)	109.5
C(41)-C(40)-H(40C)	109.5	C(46)-C(47)-H(47E)	109.5
C(41)-C(40)-H(40D)	109.5	C(46)-C(47)-H(47F)	109.5
C(40)-C(41)-C(43)	114.51(19)	H(47D)-C(47)-H(47E)	109.5
O(7)-C(41)-C(40)	112.0(2)	H(47D)-C(47)-H(47F)	109.5
O(7)-C(41)-C(43)	103.63(17)	H(47E)-C(47)-H(47F)	109.5
O(7)-C(41)-O(8)	111.47(18)	O(9)-C(48)-C(46)	125.2(2)
O(8)-C(41)-C(40)	104.25(19)	O(9)-C(48)-O(10)	123.3(2)
O(8)-C(41)-C(43)	111.22(19)	O(10)-C(48)-C(46)	111.3(2)
H(42D)-C(42)-H(42E)	109.5	C(17)-O(2)-C(13)	114.41(17)
H(42D)-C(42)-H(42F)	109.5	C(20)-O(3)-C(18)	109.47(17)
H(42E)-C(42)-H(42F)	109.5	C(20)-O(4)-C(23)	106.80(17)
O(8)-C(42)-H(42D)	109.5	C(41)-O(7)-C(37)	117.52(17)
O(8)-C(42)-H(42E)	109.5	C(41)-O(8)-C(42)	116.50(18)

C(48)-O(10)-C(11)	116.94(17)	C(33)-C(32)-H(32)	109.2
C(26)-C(27)-H(27E)	108.2	O(6)-C(32)-C(35)	106.0(4)
C(26)-C(27)-H(27F)	108.2	O(6)-C(32)-H(32)	109.2
C(26)-C(27)-C(28)	116.2(3)	O(6)-C(32)-C(33)	108.9(4)
H(27E)-C(27)-H(27F)	107.4	C(28)-C(33)-H(33)	108.7
C(28)-C(27)-H(27E)	108.2	C(28)-C(33)-C(34)	110.3(3)
C(28)-C(27)-H(27F)	108.2	C(32)-C(33)-C(28)	109.1(3)
C(27)-C(28)-H(28)	108.9	C(32)-C(33)-H(33)	108.7
C(27)-C(28)-C(33)	114.7(3)	C(32)-C(33)-C(34)	111.2(3)
C(33)-C(28)-H(28)	108.9	C(34)-C(33)-H(33)	108.7
O(5)-C(28)-C(27)	104.5(3)	C(33)-C(34)-H(34G)	109.5
O(5)-C(28)-H(28)	108.9	C(33)-C(34)-H(34H)	109.5
O(5)-C(28)-C(33)	110.8(2)	C(33)-C(34)-H(34I)	109.5
C(30)-C(29)-C(31)	111.2(4)	H(34G)-C(34)-H(34H)	109.5
O(5)-C(29)-C(30)	105.8(3)	H(34G)-C(34)-H(34I)	109.5
O(5)-C(29)-C(31)	112.2(3)	H(34H)-C(34)-H(34I)	109.5
O(6)-C(29)-C(30)	104.4(3)	C(29)-O(5)-C(28)	115.0(2)
O(6)-C(29)-C(31)	112.4(3)	C(29)-O(6)-C(32)	114.9(3)
O(6)-C(29)-O(5)	110.4(3)	C(26)-C(27')-H(27G)	109.0
C(29)-C(30)-H(30G)	109.5	C(26)-C(27')-H(27H)	109.0
C(29)-C(30)-H(30H)	109.5	C(26)-C(27')-C(28')	112.8(12)
C(29)-C(30)-H(30I)	109.5	H(27G)-C(27')-H(27H)	107.8
H(30G)-C(30)-H(30H)	109.5	C(28')-C(27')-H(27G)	109.0
H(30G)-C(30)-H(30I)	109.5	C(28')-C(27')-H(27H)	109.0
H(30H)-C(30)-H(30I)	109.5	C(27')-C(28')-H(28')	108.4
C(29)-C(31)-H(31G)	109.5	C(33')-C(28')-C(27')	113.9(13)
C(29)-C(31)-H(31H)	109.5	C(33')-C(28')-H(28')	108.4
C(29)-C(31)-H(31I)	109.5	O(5')-C(28')-C(27')	106.1(11)
H(31G)-C(31)-H(31H)	109.5	O(5')-C(28')-H(28')	108.4
H(31G)-C(31)-H(31I)	109.5	O(5')-C(28')-C(33')	111.5(12)
H(31H)-C(31)-H(31I)	109.5	C(31')-C(29')-C(30')	111.0(15)
C(35)-C(32)-H(32)	109.2	O(5')-C(29')-C(30')	107.7(14)
C(33)-C(32)-C(35)	114.3(3)	O(5')-C(29')-C(31')	111.7(13)

O(5')-C(29')-O(6')	112.1(12)
O(6')-C(29')-C(30')	104.5(13)
O(6')-C(29')-C(31')	109.6(14)
C(29')-C(30')-H(30J)	109.5
C(29')-C(30')-H(30K)	109.5
C(29')-C(30')-H(30L)	109.5
H(30J)-C(30')-H(30K)	109.5
H(30J)-C(30')-H(30L)	109.5
H(30K)-C(30')-H(30L)	109.5
C(29')-C(31')-H(31J)	109.5
C(29')-C(31')-H(31K)	109.5
C(29')-C(31')-H(31L)	109.5
H(31J)-C(31')-H(31K)	109.5
H(31J)-C(31')-H(31L)	109.5
H(31K)-C(31')-H(31L)	109.5
C(35)-C(32')-H(32')	105.7
C(35)-C(32')-C(33')	120.4(18)
C(33')-C(32')-H(32')	105.7
O(6')-C(32')-C(35)	105.4(18)
O(6')-C(32')-H(32')	105.7
O(6')-C(32')-C(33')	112.7(17)
C(28')-C(33')-C(32')	109.3(15)
C(28')-C(33')-H(33')	106.8
C(28')-C(33')-C(34')	116.8(14)
C(32')-C(33')-H(33')	106.8
C(32')-C(33')-C(34')	109.8(15)
C(34')-C(33')-H(33')	106.8
C(33')-C(34')-H(34J)	109.5
C(33')-C(34')-H(34K)	109.5
C(33')-C(34')-H(34L)	109.5
H(34J)-C(34')-H(34K)	109.5
H(34J)-C(34')-H(34L)	109.5
H(34K)-C(34')-H(34L)	109.5
C(29')-O(5')-C(28')	117.0(11)
C(29')-O(6')-C(32')	115.1(16)

Symmetry transformations used to generate equivalent atoms:

Table S8. Anisotropic displacement parameters ($Å^2$x 10^3) for **1a**. The anisotropic displacement factor exponent takes the form: $-2\Pi^2$[h^2 $a^{*2}U^{11}$ + ... + 2 h k a* b* U^{12}]

	U^{11}	U^{22}	U^{33}	U^{23}	U^{13}	U^{12}
C(1A)	120(4)	50(2)	122(4)	6(2)	92(4)	-10(2)
C(2A)	45(2)	36(2)	52(2)	5(1)	11(1)	-4(1)
C(3A)	47(2)	37(2)	44(2)	-3(1)	15(1)	-7(1)
C(4A)	134(4)	41(2)	106(3)	-13(2)	84(3)	-13(2)
C(5A)	40(2)	39(2)	46(2)	-2(1)	19(1)	-6(1)
C(6A)	39(2)	36(2)	42(2)	-7(1)	18(1)	-9(1)
C(7A)	58(2)	36(2)	67(2)	-9(1)	37(2)	-8(2)
C(8A)	35(1)	39(2)	36(1)	-3(1)	16(1)	-5(1)
C(9A)	32(1)	36(1)	30(1)	-1(1)	12(1)	-6(1)
C(10A)	36(1)	48(2)	39(2)	7(1)	3(1)	-7(1)
C(11A)	26(1)	32(1)	29(1)	6(1)	12(1)	1(1)
C(12A)	28(1)	42(2)	31(1)	1(1)	12(1)	2(1)
C(13A)	30(1)	52(2)	28(1)	0(1)	10(1)	1(1)
C(14A)	44(2)	44(2)	33(1)	-2(1)	7(1)	-8(1)
C(15A)	47(2)	36(1)	34(1)	-4(1)	11(1)	3(1)
C(16A)	46(2)	38(2)	27(1)	-2(1)	12(1)	4(1)
C(17A)	33(1)	40(2)	22(1)	-4(1)	5(1)	5(1)
C(35A)	27(1)	32(1)	28(1)	-2(1)	3(1)	0(1)
C(36A)	33(1)	45(2)	36(1)	2(1)	-3(1)	-2(1)
C(37A)	23(1)	35(1)	29(1)	-1(1)	4(1)	-3(1)
C(38A)	34(1)	39(2)	35(1)	-4(1)	10(1)	4(1)
C(39A)	38(2)	54(2)	33(1)	-5(1)	13(1)	1(1)
C(40A)	31(1)	61(2)	26(1)	3(1)	10(1)	-2(1)
C(41A)	24(1)	43(2)	31(1)	6(1)	8(1)	-6(1)
C(42A)	47(2)	49(2)	99(3)	6(2)	0(2)	-18(2)
C(43A)	37(2)	76(2)	55(2)	33(2)	17(2)	10(2)
C(44A)	31(1)	42(2)	37(2)	10(1)	11(1)	-1(1)

C(45A)	29(1)	35(1)	28(1)	5(1)	5(1)	-2(1)
C(46A)	26(1)	31(1)	26(1)	5(1)	6(1)	-1(1)
C(47A)	45(2)	31(1)	42(2)	3(1)	15(1)	-6(1)
C(48A)	26(1)	32(1)	26(1)	0(1)	8(1)	-1(1)
O(1A)	58(1)	38(1)	68(2)	8(1)	21(1)	-1(1)
O(2A)	32(1)	49(1)	28(1)	-3(1)	8(1)	9(1)
O(7A)	26(1)	30(1)	25(1)	2(1)	6(1)	-1(1)
O(8A)	28(1)	53(1)	57(1)	17(1)	9(1)	-12(1)
O(9A)	38(1)	31(1)	55(1)	-9(1)	25(1)	-5(1)
O(10A)	28(1)	28(1)	29(1)	1(1)	12(1)	0(1)
C(18A)	29(4)	36(2)	25(2)	-6(1)	3(3)	7(3)
C(19A)	37(4)	44(2)	37(3)	-19(2)	-7(3)	18(4)
C(20A)	31(2)	46(3)	38(4)	-7(3)	6(3)	7(2)
C(21A)	36(2)	99(6)	41(2)	-8(4)	10(2)	19(4)
C(22A)	50(2)	45(2)	49(3)	12(2)	21(2)	14(2)
C(23A)	22(3)	46(3)	36(4)	-14(3)	2(2)	3(2)
C(24A)	31(2)	39(2)	28(3)	-5(2)	5(2)	6(2)
C(25A)	48(4)	40(2)	51(4)	-17(2)	14(2)	13(3)
C(26A)	31(2)	67(4)	24(2)	1(2)	4(2)	16(2)
C(27A)	33(2)	37(2)	23(2)	-3(2)	7(1)	0(2)
C(28A)	30(2)	28(3)	22(2)	0(2)	2(1)	4(2)
C(29A)	40(3)	28(3)	17(2)	-7(2)	6(2)	-3(2)
C(30A)	53(3)	31(2)	27(2)	-1(2)	15(2)	-4(2)
C(31A)	40(3)	45(3)	18(2)	-8(2)	10(2)	-9(2)
C(32A)	28(3)	21(4)	20(2)	-1(3)	1(1)	4(3)
C(33A)	32(2)	34(4)	20(3)	-3(3)	2(2)	0(3)
C(34A)	46(2)	43(4)	43(3)	-5(4)	20(2)	2(4)
O(3A)	45(3)	32(2)	25(1)	-2(1)	18(2)	10(2)
O(4A)	31(1)	36(1)	39(1)	-5(1)	14(1)	-2(1)
O(5A)	37(2)	30(2)	18(1)	-4(1)	10(1)	0(2)
O(6A)	27(3)	25(2)	25(2)	-2(1)	4(2)	5(2)
C(18B)	29(4)	36(2)	25(2)	-6(1)	3(3)	7(3)
C(19B)	37(4)	44(2)	37(3)	-19(2)	-7(3)	18(4)

C(20B)	31(2)	46(3)	38(4)	-7(3)	6(3)	7(2)
C(21B)	36(2)	99(6)	41(2)	-8(4)	10(2)	19(4)
C(22B)	50(2)	45(2)	49(3)	12(2)	21(2)	14(2)
C(23B)	22(3)	46(3)	36(4)	-14(3)	2(2)	3(2)
C(24B)	31(2)	39(2)	28(3)	-5(2)	5(2)	6(2)
C(25B)	48(4)	40(2)	51(4)	-17(2)	14(2)	13(3)
C(26B)	31(2)	67(4)	24(2)	1(2)	4(2)	16(2)
C(27B)	33(2)	37(2)	23(2)	-3(2)	7(1)	0(2)
C(28B)	30(2)	28(3)	22(2)	0(2)	2(1)	4(2)
C(29B)	40(3)	28(3)	17(2)	-7(2)	6(2)	-3(2)
C(30B)	53(3)	31(2)	27(2)	-1(2)	15(2)	-4(2)
C(31B)	40(3)	45(3)	18(2)	-8(2)	10(2)	-9(2)
C(32B)	28(3)	21(4)	20(2)	-1(3)	1(1)	4(3)
C(33B)	32(2)	34(4)	20(3)	-3(3)	2(2)	0(3)
C(34B)	46(2)	43(4)	43(3)	-5(4)	20(2)	2(4)
O(3B)	45(3)	32(2)	25(1)	-2(1)	18(2)	10(2)
O(4B)	31(1)	36(1)	39(1)	-5(1)	14(1)	-2(1)
O(5B)	37(2)	30(2)	18(1)	-4(1)	10(1)	0(2)
O(6B)	27(3)	25(2)	25(2)	-2(1)	4(2)	5(2)
C(1)	42(2)	22(1)	88(3)	1(2)	-22(2)	5(1)
C(2)	32(1)	23(1)	30(1)	-2(1)	10(1)	4(1)
C(3)	27(1)	24(1)	34(1)	-5(1)	5(1)	-1(1)
C(4)	54(2)	23(1)	114(3)	-1(2)	-32(2)	-5(1)
C(5)	21(1)	24(1)	24(1)	-3(1)	5(1)	0(1)
C(6)	21(1)	26(1)	21(1)	-3(1)	7(1)	2(1)
C(7)	25(1)	23(1)	32(1)	-3(1)	3(1)	1(1)
C(8)	20(1)	25(1)	24(1)	-2(1)	4(1)	2(1)
C(9)	21(1)	22(1)	29(1)	2(1)	5(1)	3(1)
C(10)	32(1)	29(1)	46(2)	7(1)	18(1)	3(1)
C(11)	17(1)	16(1)	31(1)	3(1)	4(1)	1(1)
C(12)	19(1)	20(1)	33(1)	2(1)	2(1)	1(1)
C(13)	20(1)	24(1)	31(1)	-1(1)	2(1)	3(1)
C(14)	33(1)	21(1)	38(1)	-2(1)	6(1)	8(1)

C(15)	33(1)	19(1)	37(1)	-8(1)	3(1)	-2(1)
C(16)	27(1)	22(1)	32(1)	-7(1)	2(1)	2(1)
C(17)	20(1)	23(1)	25(1)	-5(1)	4(1)	-1(1)
C(18)	21(1)	25(1)	22(1)	-3(1)	4(1)	-2(1)
C(19)	38(1)	31(1)	32(1)	-8(1)	16(1)	-6(1)
C(20)	25(1)	23(1)	25(1)	-3(1)	1(1)	-2(1)
C(21)	24(1)	38(1)	27(1)	1(1)	2(1)	1(1)
C(22)	36(1)	30(1)	32(1)	6(1)	-3(1)	-5(1)
C(23)	22(1)	22(1)	22(1)	0(1)	5(1)	0(1)
C(24)	25(1)	23(1)	24(1)	-2(1)	5(1)	-4(1)
C(25)	30(1)	25(1)	44(2)	-7(1)	3(1)	-7(1)
C(26)	25(1)	30(1)	29(1)	2(1)	2(1)	-4(1)
C(35)	22(1)	22(1)	25(1)	2(1)	2(1)	-2(1)
C(36)	27(1)	34(1)	37(1)	12(1)	6(1)	0(1)
C(37)	18(1)	23(1)	27(1)	3(1)	6(1)	1(1)
C(38)	26(1)	30(1)	31(1)	0(1)	2(1)	-6(1)
C(39)	26(1)	41(2)	24(1)	-2(1)	-1(1)	-8(1)
C(40)	24(1)	36(1)	23(1)	0(1)	4(1)	-1(1)
C(41)	19(1)	27(1)	21(1)	4(1)	3(1)	6(1)
C(42)	38(2)	30(1)	40(2)	6(1)	18(1)	13(1)
C(43)	24(1)	21(1)	22(1)	0(1)	5(1)	4(1)
C(44)	28(1)	29(1)	31(1)	5(1)	9(1)	5(1)
C(45)	25(1)	18(1)	24(1)	1(1)	7(1)	2(1)
C(46)	25(1)	19(1)	40(1)	0(1)	0(1)	2(1)
C(47)	34(2)	34(2)	133(4)	-33(2)	-29(2)	14(1)
C(48)	20(1)	23(1)	30(1)	1(1)	4(1)	1(1)
O(1)	36(1)	20(1)	61(1)	6(1)	9(1)	2(1)
O(2)	20(1)	24(1)	28(1)	-4(1)	4(1)	-1(1)
O(3)	28(1)	23(1)	21(1)	-3(1)	1(1)	-1(1)
O(4)	26(1)	21(1)	29(1)	-1(1)	-2(1)	1(1)
O(7)	20(1)	18(1)	21(1)	2(1)	2(1)	0(1)
O(8)	22(1)	33(1)	30(1)	7(1)	7(1)	10(1)
O(9)	23(1)	22(1)	50(1)	-8(1)	-1(1)	2(1)

O(10)	18(1)	19(1)	33(1)	1(1)	2(1)	1(1)
C(27)	25(2)	28(2)	41(2)	11(1)	-1(1)	-6(1)
C(28)	21(1)	25(2)	33(2)	4(1)	0(1)	-2(2)
C(29)	29(2)	22(1)	46(2)	-8(2)	-3(2)	4(1)
C(30)	44(2)	24(2)	83(3)	-18(2)	-15(2)	5(2)
C(31)	35(2)	42(2)	38(2)	-15(2)	3(2)	11(2)
C(32)	24(1)	16(1)	22(3)	2(2)	4(2)	-2(1)
C(33)	23(1)	22(2)	24(2)	2(1)	1(1)	-6(2)
C(34)	28(2)	34(2)	33(2)	-10(2)	-1(1)	3(2)
O(5)	29(1)	19(1)	52(2)	-3(1)	-9(1)	3(1)
O(6)	23(1)	15(1)	38(2)	-3(1)	-1(1)	0(1)
C(27')	25(2)	28(2)	41(2)	11(1)	-1(1)	-6(1)
C(28')	21(1)	25(2)	33(2)	4(1)	0(1)	-2(2)
C(29')	29(2)	22(1)	46(2)	-8(2)	-3(2)	4(1)
C(30')	44(2)	24(2)	83(3)	-18(2)	-15(2)	5(2)
C(31')	35(2)	42(2)	38(2)	-15(2)	3(2)	11(2)
C(32')	24(1)	16(1)	22(3)	2(2)	4(2)	-2(1)
C(33')	23(1)	22(2)	24(2)	2(1)	1(1)	-6(2)
C(34')	28(2)	34(2)	33(2)	-10(2)	-1(1)	3(2)
O(5')	29(1)	19(1)	52(2)	-3(1)	-9(1)	3(1)
O(6')	23(1)	15(1)	38(2)	-3(1)	-1(1)	0(1)

Table S9. Hydrogen coordinates (x 10^4) and isotropic displacement parameters ($Å^2$x 10^3) for **1a**.

	x	y	z	U(eq)
H(1AA)	5343	2207	4313	133
H(1AB)	4560	1109	4310	133
H(1AC)	4333	2158	3978	133
H(4AA)	7236	633	3496	129
H(4AB)	6572	1152	3089	129
H(4AC)	6181	3	3308	129
H(5A)	5564	3195	3746	48
H(7AA)	7804	2649	3358	76
H(7AB)	7894	3985	3182	76
H(7AC)	7196	2992	2919	76
H(8A)	5677	5050	3487	43
H(9A)	7081	5601	2977	38
H(10A)	5534	6561	2784	62
H(10B)	6360	7588	2934	62
H(10C)	5597	7156	3221	62
H(11A)	8012	5722	3659	34
H(12A)	6659	6663	3950	39
H(12B)	6917	7905	3745	39
H(13A)	8690	7490	4113	44
H(14A)	7725	9221	4241	48
H(14B)	8534	8778	4622	48
H(15A)	6991	9177	4840	46
H(15B)	6391	8292	4501	46
H(16A)	6716	7233	5118	43
H(16B)	7904	7527	5169	43
H(17A)	6785	6038	4547	38
H(17B)	6784	5964	4561	38
H(35A)	12704	6409	3950	35
H(35B)	12616	6365	3953	35

H(36A)	13970	4352	3940	58
H(36B)	13617	4860	4337	58
H(36C)	14320	5673	4107	58
H(37A)	13686	6434	3431	35
H(38A)	12417	4478	3075	43
H(38B)	13631	4428	3194	43
H(39A)	13108	4813	2487	49
H(39B)	13793	5903	2701	49
H(40A)	12362	6756	2291	47
H(40B)	11615	5922	2494	47
H(42A)	12955	9702	3063	99
H(42B)	14056	9127	3211	99
H(42C)	13114	8679	3405	99
H(43A)	11651	9042	2295	82
H(43B)	10490	9252	2332	82
H(43C)	10832	7996	2160	82
H(44A)	11364	8829	2974	43
H(45A)	10282	6629	2760	37
H(47A)	9841	8871	3588	58
H(47B)	8968	9296	3226	58
H(47C)	10135	9519	3200	58
H(19A)	9237	5318	5114	62
H(19B)	8860	4520	5455	62
H(19C)	8706	5960	5448	62
H(21A)	4996	3314	4942	87
H(21B)	5472	4312	4686	87
H(21C)	4995	4714	5068	87
H(22A)	5865	3966	5758	69
H(22B)	6981	3402	5804	69
H(22C)	6017	2614	5606	69
H(23A)	7044	3949	4524	42
H(24A)	8941	3072	4971	39
H(25A)	7887	1363	4856	69

H(25B)	8706	1273	4565	69
H(25C)	7570	1693	4388	69
H(26A)	8955	2933	4177	49
H(26B)	8466	4223	4236	49
H(27A)	10279	3598	4693	37
H(27B)	9817	4915	4578	37
H(28A)	9960	4339	3873	32
H(30A)	11356	925	3814	54
H(30B)	12346	1548	4069	54
H(30C)	12236	1410	3591	54
H(31A)	10986	2675	3136	51
H(31B)	10317	3654	3321	51
H(31C)	10082	2238	3354	51
H(32A)	11366	5121	3552	28
H(33A)	11812	4889	4420	35
H(34A)	10517	6571	3916	64
H(34B)	11491	6922	4245	64
H(34C)	10490	6417	4386	64
H(19D)	9441	5342	5060	62
H(19E)	9122	4446	5389	62
H(19F)	8980	5879	5430	62
H(21D)	4861	4124	4891	87
H(21E)	5397	5387	4825	87
H(21F)	5202	5019	5264	87
H(22D)	6294	3384	5650	69
H(22E)	7124	2708	5443	69
H(22F)	5942	2459	5286	69
H(23B)	7686	4298	4427	42
H(24B)	8885	3000	5031	39
H(25D)	7423	1708	4925	69
H(25E)	8345	1053	4769	69
H(25F)	7450	1659	4453	69
H(26C)	9224	2086	4382	49

H(26D)	8707	3296	4200	49
H(27C)	10570	2949	4627	37
H(27D)	10078	4255	4571	37
H(28B)	10066	3815	3842	32
H(30D)	11826	679	3703	54
H(30E)	12699	1382	4002	54
H(30F)	12694	1386	3527	54
H(31D)	11304	2547	3066	51
H(31E)	10515	3325	3265	51
H(31F)	10447	1875	3259	51
H(32B)	11454	4837	3516	28
H(33B)	11849	4631	4372	35
H(34D)	10222	6008	3934	64
H(34E)	11297	6616	4109	64
H(34F)	10633	6033	4410	64
H(1A)	627	7895	713	83
H(1B)	28	9093	801	83
H(1C)	231	8037	1131	83
H(4A)	3733	9072	1379	104
H(4B)	3494	8728	1813	104
H(4C)	2994	9911	1584	104
H(5)	1584	6826	1233	27
H(7A)	4320	6934	1577	41
H(7B)	4358	5703	1831	41
H(7C)	3957	6926	2004	41
H(8)	1877	4925	1387	28
H(9)	3648	4082	1907	29
H(10D)	2167	3182	2025	51
H(10E)	2814	2148	1854	51
H(10F)	1866	2754	1566	51
H(11)	4063	4137	1237	26
H(12C)	2432	3338	885	29
H(12D)	2810	2033	1059	29

H(13)	4345	2570	742	31
H(14C)	3809	1335	205	37
H(14D)	3313	807	567	37
H(15C)	1741	1693	300	36
H(15D)	2098	900	-48	36
H(16C)	2683	2661	-336	33
H(16D)	1520	2872	-295	33
H(17)	2033	3956	311	27
H(19G)	3981	4894	-244	49
H(19H)	3295	5860	-525	49
H(19I)	3102	4438	-598	49
H(21G)	-9	6500	164	45
H(21H)	810	5487	336	45
H(21I)	-77	5178	-35	45
H(22G)	-109	6410	-683	51
H(22H)	904	7094	-747	51
H(22I)	204	7668	-457	51
H(23)	2470	5929	448	26
H(24)	3957	6908	27	29
H(25G)	2988	8607	152	50
H(25H)	4091	8674	428	50
H(25I)	3136	8256	619	50
H(26E)	4101	6393	871	34
H(26F)	4410	5430	561	34
H(26G)	4448	6833	861	34
H(26H)	4082	5528	687	34
H(35)	8481	3772	1016	28
H(35C)	8546	3748	1017	28
H(36D)	9736	5855	1206	49
H(36E)	9088	5564	770	49
H(36F)	10015	4704	958	49
H(37)	9917	3378	1526	27
H(38C)	9052	5096	2008	35

H(38D)	10173	5163	1906	35
H(39C)	10655	3399	2295	37
H(39D)	10211	4371	2571	37
H(40C)	9485	2406	2631	33
H(40D)	8625	3380	2464	33
H(42D)	9225	1143	1422	52
H(42E)	9568	-65	1672	52
H(42F)	10406	836	1555	52
H(43)	7879	786	1806	26
H(44B)	8696	163	2462	43
H(44C)	7488	44	2405	43
H(44D)	8055	1156	2655	43
H(45)	7065	2911	2107	27
H(47D)	6452	206	1548	109
H(47E)	5576	886	1244	109
H(47F)	5329	280	1646	109
H(27E)	5421	7694	738	38
H(27F)	5746	6683	447	38
H(28)	5790	5816	1228	32
H(30G)	7508	8756	1818	80
H(30H)	8215	8529	1491	80
H(30I)	8552	8028	1940	80
H(31G)	7511	6327	2182	58
H(31H)	6625	5708	1865	58
H(31I)	6478	7048	2028	58
H(32)	7474	4642	1502	25
H(33)	7323	5802	727	28
H(34G)	6180	3785	953	49
H(34H)	6982	3704	655	49
H(34I)	5939	4425	521	49
H(27G)	5534	6798	393	38
H(27H)	5285	5379	357	38
H(28')	5756	5496	1127	32

H(30J)	7239	8892	1491	80
H(30K)	7902	8543	1158	80
H(30L)	8346	8318	1625	80
H(31J)	7458	6670	1996	58
H(31K)	6547	5918	1736	58
H(31L)	6402	7326	1827	58
H(32')	7520	4692	1446	25
H(33')	7226	5329	618	28
H(34J)	6396	3311	980	49
H(34K)	7236	3229	698	49
H(34L)	6106	3653	513	49

The crystal structure data for derivatized **1a** of marinolide A is available in the CCDC/FIZ Karlsruhe depository under deposition number 2033847.

Supplemental References

1. (a) Haydock, S. F.; Aparicio, J. F.; Molnar, I.; Schwecke, T.; Khaw, L. E.; Konig, A.; Marsden, A. F.; Galloway, I. S.; Staunton, J.; Leadlay, P. F., Divergent sequence motifs correlated with the substrate specificity of (methyl)malonyl-CoA:acyl carrier protein transacylase domains in modular polyketide synthases. *FEBS Lett* **1995,** *374* (2), 246-8; (b) Dunn, B. J.; Cane, D. E.; Khosla, C., Mechanism and specificity of an acyltransferase domain from a modular polyketide synthase. *Biochemistry* **2013,** *52* (11), 1839-41; (c) Dunn, B. J.; Khosla, C., Engineering the acyltransferase substrate specificity of assembly line polyketide synthases. *J R Soc Interface* **2013,** *10* (85), 20130297.

2. (a) Keatinge-Clay, A. T., A tylosin ketoreductase reveals how chirality is determined in polyketides. *Chem Biol* **2007,** *14* (8), 898-908; (b) Zheng, J.; Taylor, C. A.; Piasecki, S. K.; Keatinge-Clay, A. T., Structural and functional analysis of A-type ketoreductases from the amphotericin modular polyketide synthase. *Structure* **2010,** *18* (8), 913-22; (c) Bailey, C. B.; Pasman, M. E.; Keatinge-Clay, A. T., Substrate structure-activity relationships guide rational engineering of modular polyketide synthase ketoreductases. *Chem Commun (Camb)* **2016,** *52* (4), 792-5; (d) Xie, X.; Garg, A.; Keatinge-Clay, A. T.; Khosla, C.; Cane, D. E., Epimerase and reductase activities of polyketide synthase ketoreductase domains utilize the same conserved tyrosine and serine residues. *Biochemistry* **2016,** *55* (8), 1179-86; (e) Caffrey, P., Conserved amino acid residues correlating with ketoreductase stereospecificity in modular polyketide synthases. *Chembiochem* **2003,** *4* (7), 654-7; (f) Valenzano, C. R.; Lawson, R. J.; Chen, A. Y.; Khosla, C.; Cane, D. E., The biochemical basis for stereochemical control in polyketide biosynthesis. *J Am Chem Soc* **2009,** *131* (51), 18501-11.

3. Keatinge-Clay, A. Crystal structure of the erythromycin polyketide synthase dehydratase. *J Mol Biol* **2008,** *384*(4) 941-53.

4. Kwan, D. H.; Sun, Y.; Schulz, F.; Hong, H.; Popovic, B.; Sim-Stark, J. C. C.; Haydock, S. F.; Leadlay, P., Prediction and manipulation of the stereochemistry of enoylreduction in modular polyketide synthases. *Chem Biol* **2008,** *15*(11) 1231-40.

5. Hirsch, M.; Fitzgerald, B. J.; Keatinge-Clay, A. T., How *cis*-acyltransferase assembly-line ketosynthases gatekeep for processed polyketide intermediates. *ACS Chem Biol* **2021,** *16*(11) 2515-26.
